# High-Throughput Screening of Potent Drug-like Molecules Targeting 17β-HSD10 for the Treatment of Alzheimer’s Disease and Cancer

**DOI:** 10.1021/acschembio.5c00110

**Published:** 2025-06-18

**Authors:** Laura Aitken, Gemma Baillie, Andrew Pannifer, Angus Morrison, Louise L. Major, Magnus S. Alphey, Ritika Sethi, Martin Timmerman, John Robinson, Jennifer Riley, Yoko Shishikura, Lizbe Koekemoer, Frank Von Delft, Helma Rutjes, Kevin D. Read, Philip S. Jones, Stuart P. McElroy, Terry K. Smith, Frank J. Gunn-Moore

**Affiliations:** † 7486 University of St. Andrews School of Chemistry, Biomolecular Sciences Building, North Haugh, St. Andrews KY16 9TF, U.K.; ‡ 523043 BioAscent Discovery Ltd, Bo’Ness Road, Newhouse, Lanarkshire ML1 5UH, U.K.; § Excientia, Oxford Science Park, The Schrödinger Building, Oxford OX4 4GE, U.K.; ∥ Biomedical Science Research Complex, 98461 University of St. Andrews School of Biology, North Haugh, St. Andrews KY16 9ST, U.K.; ⊥ Glaxo Smith Kline Biologicals, rue de l’Institut 89, 1330 Rixensart, Belgium; # European Lead Factory, Pivot Park−Banting Building (RE600), Kloosterstraat 9, 5349 AB Oss, The Netherlands; ∇ Wellcome Centre of Anti-infectives Research, Drug Discovery Unit, 98264 University of Dundee School of Life Sciences, Dow Street, Dundee DD1 5EH, U.K.; ○ 6396 University of Oxford Centre for Medicines Discovery, Old Road Campus Research Building, Oxford OX3 7FZ, U.K.

## Abstract

In this study, the first industrial-scale high-throughput screening of nearly 350,000 drug-like molecules targeting the enzyme 17β-HSD10, a promising therapeutic target for Alzheimer’s disease and cancers, is presented. Two novel series of potent 17β-HSD10 inhibitors that demonstrate low nanomolar potency against both the enzyme and *in vivo* cellular assays with minimal cytotoxicity were identified. These inhibitors were characterized further through a series of assays demonstrating ligand–protein interactions and co-crystallography, revealing un-/non-competitive inhibition with respect to the cofactor NADH, unlike previously published inhibitors. This work significantly advances the development of 17β-HSD10-targeting therapeutics, offering new potential leads for treating Alzheimer’s disease and cancers.

## Graphical abstract

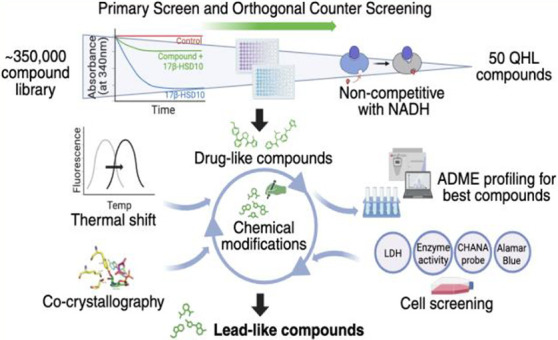

## Introduction

17β-Hydroxysteroid type 10 (17β-HSD10) is an enzyme that has been reported to have a wide range of different substrates and functions.^1^ A link to Alzheimer’s disease progression has come from two separate studies showing 17β-HSD10 to have increased expression in the brains of Alzheimer’s disease patients^2,3^ but also its ability to bind to the amyloid beta-peptide (Aβ) binding protein, initially via a yeast two-hybrid system, which has subsequently been confirmed using a number of other techniques.^2,4,5^ 17β-HSD10 interacts with both major plaque forming isoforms of Aβ, namely, Aβ(1–40) and Aβ(1–42), with a reported change of the enzyme structure and subsequently modification of its normal function.^2,4,5^ *In vitro* and *in vivo* experiments have shown that the interaction between 17β-HSD10 and Aβ is cytotoxic and the function of 17β-HSD10 is diminished, with a resulting buildup of reactive oxygen species (ROS) and toxins leading to mitochondrial dysfunction.^4^ Using site-directed mutagenesis and surface plasmon resonance, Lustbader et al.^4^ identified the L_D_ loop of the 17β-HSD10 protein as the binding site for Aβ and subsequently synthesized a 28-amino-acid peptide encompassing this region (amino acids 92–120), which was termed the 17β-HSD10-decoy peptide (17β-HSD10-DP). Again, using surface plasmon resonance, it was shown that this 17β-HSD10-DP could inhibit the binding of 17β-HSD10 to Aβ(1–40) and Aβ(1–42).^6^ Significantly, the inhibition of the interaction between 17β-HSD10 and Aβ has been shown to translate into a cytoprotective effect in cell culture experiments.^4,6,7^ In addition, work with transgenic animals showed that the 17β-HSD10-DP could reverse both activated proteins from the 17β-HSD10–Aβ interaction and also improve memory function.^6^ This collective work demonstrates that inhibition of the 17β-HSD10–Aβ interaction may offer a novel therapeutic avenue for the treatment of Alzheimer’s disease.^7–11^

Other than the disruption of the 17β-HSD10–Aβ interaction, there is a second approach that may hold merit in treating Alzheimer’s disease: the direct modulation of 17β-HSD10 enzyme activity. In vitro experiments with SH-SY5Y cells administered with the 17β-HSD10 inhibitor, AG18051, show a reduction in mitochondrial dysfunction and oxidative stress associated with the interaction between 17β-HSD10 and Aβ and protect cultured SH-SY5Y cells from Aβ-mediated cytotoxicity,^7,8^ proving that inhibiting 17β-HSD10 activity in an amyloid-rich environment may also be a viable therapeutic approach in the treatment of Alzheimer’s disease. In addition, as a substrate of 17β-HSD10 is the neuro-protective hormone estradiol, the partial inhibition of this enzyme, which has elevated expression and activity in Alzheimer’s disease patients, would maintain physiological levels of this steroid and thus be a viable therapeutic approach.^1^ Besides Alzheimer’s disease, 17β-HSD10 activity has also been shown to be a potential target in specific cancers; specifically, the overexpression of 17β-HSD10 in some cancers like prostate cancer, bone cancer, colorectal cancer, and osteosarcomas is considered to be part of the mechanism of action and/or act as an outcome predictor (reviewed by Vinklarova et al.^1^). Indeed, the repurposed antipsychotic risperidone, which has some capabilities of inhibiting 17β-HSD10 activity, is currently in clinical trials for pancreatic ductal adenocarcinoma.

Therefore, there have been several approaches to develop inhibitors against 17β-HSD10 activity ranging from compounds designed at preventing the 17β-HSD10–Aβ interaction, such as frentizole and derivatives^8,9,11–16^ to utilizing a fused pyrazole compound AG18051, which acts as a suicide inhibitor,^17^ and repurposing of other compounds such as risperidone,^18,19^ methylene blue,^20^ or other FDA-approved compounds.^21^ In all cases, none of these compounds have been developed with industrial input and so lack the desired potency/efficacy, besides having various other disadvantages (reviewed by Vinklarova et al.^1^).

In this study, a highly productive collaboration with the European Lead Factory allowed the first industrial screening of nearly 350,000 drug-like molecules against 17β-HSD10. From this high-throughput screen, we identified a series of clustered molecules and a singleton analogue exhibiting low nM/μM potency in our recombinant 17β-HSD10 enzyme activity assays and cellular assays, respectively, with very little cytotoxicity in cells. Of the two distinct clusters, one features an imidazole benzamide as the key structural motif and the other series can be separated to produce one active selective enantiomer. With further characterization, we demonstrated ligand–protein interactions through co-crystallography for both series, which then aided in further structure-based design. Furthermore, we showed that our drug-like molecules are non-/uncompetitive with respect to the cofactor (NADH), offering significant enzyme specificity over other previously published direct 17β-HSD10 inhibitors, which to date have been less potent and/or are competitive for NADH. Hence, we have produced two novel series of drug-like compounds that can selectively and potently inhibit the activity of 17β-HSD10, both *in vitro* and *in vivo*, placing them as real potential leads in the treatment of either Alzheimer’s disease and/or specific cancers.

## Materials and Methods

All aqueous solutions were prepared with deionized water (Millipore, UK), and all chemicals were purchased from Sigma-Aldrich, UK, unless otherwise stated.

### Compound Library

The European Lead Factory (ELF) was a consortium of 30 partners from industry and academia that facilitated the large-scale sharing of screening compound collections.^22–24^ The ELF created a unique core screening library of nearly 350,000 compounds originating from participating pharmaceutical companies through sharing proprietary compounds securely from their screening decks.^25^ These compounds were assessed for novelty, drug likeness, and tractability in a peer-reviewed process.

### Protein Purification for Enzyme Activity Assays

Recombinant 17β-HSD10 was expressed and purified as previously described in Aitken et al.^26^

### Compound Screening

17β-HSD10 screening was carried out as described in Aitken et al.^21^ with the exception that experiments were also conducted in clear-bottom 1536-well low-volume microplates (Corning) with a final assay volume of 20 μL. For primary screening, an endpoint assay format was adopted, in which an Echo acoustic liquid dispenser (Labcyte) was used to transfer 20 nL of either DMSO control (0.5%) or test compounds (10 μM) to the assay plate. 10 μL of the 17β-HSD10 enzyme in an assay buffer (2.5 nM) was then added to the plates using the Preddator liquid handling robot (Redd and Whyte), and plates were incubated at RT for 15 min. 10 μL of the substrate mixture (100 μM acetoacetyl-CoA and 100 μM NADH) was added with the Preddator. To start the reaction, the plates were left to incubate for 35 min at RT before the absorbance at 340 nm was read on the EnVision plate reader (PerkinElmer). For the kinetic assay format and dose-response follow-up experiments, the same protocol was adopted, except that the absorbance at 340 nm was monitored constantly for 45 min from immediately after the reaction was started.

### Orthogonal Assays

Previous studies during assay development^21^ have shown that the primary screen may be susceptible to identifying redox cycling compounds and small-molecule aggregating compounds as false positives. Thus, as described in Aitken et al.^21^ Triton X-100 aggregation assays were performed to screen for compounds that could be causing inhibition via aggregation and resazurin assays to identify potential redox cycling compounds.

### Acetoacetyl-CoA and NADH Enzyme Kinetics

To determine the mode of inhibition, a matrix titration was used to calculate the kinetic parameters for acetoacetyl-CoA and NADH and the effect of 17β-HSD10 concentration upon reaction rate. Previously determined conditions as described in Aitken et al.^21^ were used. Data collected from the experiment were analyzed by plotting reaction progress curves to calculate the initial velocities, Vmax, and Km values using the Michaelis–Menten equation (XLFit, ID Business Solutions). Hanes Woolf plots were used to determine the mechanism of inhibition.

### Physiochemical Predictions

Physicochemical predictions were made using the ChemAxon Marvin suite (http://www.chemaxon.com).

### Protein Expression and Purification for Crystallography

Human 17β-HSD10 (residues 1–261) was cloned into the pNIC-CTHF vector with a TEV-cleavable C-terminal hexa-histidine tag and a Flag tag. After transformation into (BL21­(DE3) pRARE2), expression was performed in a Terrific Broth autoinduction medium (FroMedium), supplemented with 20 g/L glycerol, antifoam, 50 μg/mL kanamycin, and 34 μg/mL chloramphenicol. Cultures were grown for 4 h at 37 °C, following which the temperature was dropped to 18 °C, and the cultures were grown for another 42 h. Cells were spun at 4000 *g* for 20 min, and after discarding the supernatant, they were frozen for 2 h. After thawing, the cell pellets were resuspended in a buffer (10 mM HEPES, 5% glycerol, 500 mM NaCl, 0.5 mM TCEP ((tris­(2-carboxyethyl)­phosphine), pH 7.5) supplemented with 0.5 mg mL^–1^ lysozyme and 1 μg/mL benzonase, vortexed, and incubated at RT for 30 min. 2% Triton X-100 was added, and the cell suspensions were frozen overnight at −80 °C. On thawing, the cells were supplemented with 10 mM imidazole and stirred for 1 h at RT. Cells were then centrifuged for 1 h at 4000 × *g*, and the supernatant was applied to the Hi GraviTrap column (GE Healthcare) equilibrated with a binding buffer (10 mM HEPES, 5% glycerol, 500 mM NaCl, 0.5 mM TCEP, pH 7.5). The column was washed twice with the binding buffer supplemented with 10 mM imidazole. 17β-HSD10 was eluted with the binding buffer supplemented with 300 mM imidazole. The eluted protein was applied to a PD-10 desalting column (GE Healthcare) and eluted with a binding buffer supplemented with 10 mM imidazole. The C-terminal affinity tag was removed by TEV cleavage overnight, and uncleaved protein was removed by reverse IMAC by applying it again to a His GraviTrap column. The flow-through of 17β-HSD10 was supplemented with 1/10th the volume of 1 M arginine/1 M glutamine mix (pH 7.5) and was concentrated and purified further by size exclusion chromatography using a YARRA SEC-2000 PREP column (Phenomenex), equilibrated with a binding buffer. Fractions containing 17β-HSD10 were pooled, concentrated to approximately 0.45 mM, and stored at −80 °C.

### Crystallization

An aliquot of 0.45 mM 17β-HSD10 was incubated with 5 mM of both NADH and ESC1002033 compound at RT for 10 min. A sitting drop crystallization plate with 150 nL drops was set up with the Hampton Index Screen (Hampton Research) at 20 °C. Cocrystals were obtained in a condition containing 5 mM magnesium chloride, 5 mM cobalt chloride, 5 mM nickel chloride, 5 mM cadmium chloride, 12% PEG3350, and 0.1 M HEPES, pH 7.5. All crystals were harvested with 20% ethylene glycol as the cryo-protectant and flash-cooled in liquid nitrogen. To obtain crystals with the compound ESC1002332, a soaking-out technique was used, whereby the compound ESC1002332 was added to a final concentration of 5 mM into a crystallization drop containing co-crystals with ESC1002033 for 10 min. The crystals were harvested and flash-cooled as before.

The models were built with coot and refined iteratively with phenix and with the CCP4 package to a final Rfactor of below 20% and with reasonable stereochemistry. Ligand complexes were obtained either by co-crystallization with ESC1002033 or by co-crystallizing and then soaking out the first ligand and replacing it with another. There were four molecules in the asymmetric unit, and in some cases, the soaking procedure did not replace the original co-crystallization ligand in all molecules. The relevant ligand was built and refined in each case.

### Lactate Dehydrogenase (LDH) Cytotoxicity Assay

Cell cytotoxicity was assessed via the measurement of LDH leakage into the culture medium using a commercially available kit from Pierce (Thermo Scientific cat no. 88953). This was carried out in accordance with the kit guidelines, with the activity of LDH being calculated from the change in absorbance at 340 nm as NADH is reduced. Human embryonic kidney (HEK293) cells overexpressing 17β-HSD10 were cultured in phenol-red free media (10% FBS, 1 mM sodium pyruvate, 100 units penicillin, 0.1 mg mL^–1^ streptomycin, and 2 mM l-glutamine) and seeded at a density of 10,000 cells per well (100 μL, 96-well plates). Cells were then treated with the compound of interest at two concentrations (25 and 100 μM in DMSO) in triplicate. Treated cells were then incubated at 37 °C and CO_2_ (5%) for 24 h before the LDH assay was performed as per the manufacturer’s instructions. Spontaneous control (water) and maximum control (lysis buffer) were used in accordance with the kit guide. Absorbance was measured at 490 and 680 nm using the SpectraMaxM2e spectrophotometer. The measured LDH activity was used to calculate %cytotoxicity using the following equation (eq 1):

$$\%\;\mathrm{cytotoxicity}=\frac{\left(\mathrm{compound}\;\mathrm{treated}\;\mathrm{LDH}\;\mathrm{activity}-\mathrm{spontaneous}\;\mathrm{LDH}\;\mathrm{activity}\right)}{\left(\mathrm{maximum}\;\mathrm{LDH}\;\mathrm{activity}-\mathrm{spontaneous}\;\mathrm{LDH}\;\mathrm{activity}\right)}\times 100$$ 1

### Alamar Blue Cell Viability Assay

Cell viability was assessed via fluorescence change using the commercially available Alamar Blue from ThermoFisher. HEK293 cells overexpressing 17β-HSD10 were cultured in phenol-red free media (10% FBS, 1 mM sodium pyruvate, 100 units penicillin, 0.1 mg mL^–1^ streptomycin, and 2 mM l-glutamine) and seeded at a density of 10,000 cells per well (100 μL, 96-well plates). Cells were then treated with the compound of interest at two concentrations (25 and 100 μM in DMSO) in triplicate. Treated cells were then incubated at 37 °C and CO_2_ (5%) for 24 h before the assay was performed as per the manufacturer’s instructions. Fluorescence was measured at 530 nm excitation and 590 nm emission using the SpectraMaxM2e spectrophotometer.

### CHANA Assay: In Vitro Dose Response and EC_50_ Determination

(−)–CHANA ((−)-cyclohexenyl amino naphthalene alcohol) is a fluorogenic probe that can be used to detect 17β-HSD10 activity in living cells.^27^ HEK293 17β-HSD10 cells were seeded at a density of 10,000 cells per well (100 μL, 96-well black plates) in phenol-red free media (10% FBS, 1 mM sodium pyruvate, 100 units penicillin, 0.1 mg mL^–1^ streptomycin, and 2 mM l-glutamine). The cells were incubated at 37 °C and CO_2_ (5%) for 24 h before the media was removed from the cells and replaced with fresh media containing varying concentrations of the compound (100 μM–0.098 μM). The fluorogenic probe (−)–CHANA was then added to each well to give a final assay concentration of 20 μM. Fluorescence was immediately measured using the FLUOstar Optima microplate reader (excitation = 380 nm, emission = 520 nm, orbital averaging = 3 mm), and the initial reaction was monitored for 3–4 h. EC_50_ was calculated from the control-subtracted triplicates using nonlinear regression (four parameters) of GraphPad Prism 5 software. The Final EC_50_ and SEM values were obtained as a mean of at least three independent measurements.

### ADME Profiling

Hepatocyte isolation, hepatocyte incubations for metabolic stability, plasma protein binding (PPB) experiments, and LC/MS sample analysis were carried out as previously described in Brandon et al.^28^ and MacLeod et al.^29^

#### Intrinsic Clearance Data Analysis

XLfit (IDBS, UK) was used to calculate the exponential decay and the rate constant (*k*) from the ratio of the peak area of the test compound to the internal standard at each time point. The rate of intrinsic clearance (CLi) of each test compound was then calculated using the following calculation:

CLint(mL/min/gliver)=k/V×hepatocellularityscalingfactor

where *V* is million cells per mL, and the hepatocellularity scaling factor of 120 × 10^6^ cells/g of liver for both human and mouse was applied.^30^ Verapamil, 7-ethoxycoumarin, 7-hydroxycoumarin, and phthalazine were used as positive controls to confirm acceptable assay performance. For all intrinsic clearance measurements, lower and upper limits of quantitation were 0.5 and 50 mL/min/g liver, respectively. Values were transferred to RStudio v1.3.1093 (Posit, Boston, MA, USA) for generation of scatter plots and heatmaps using the ggplot2 package (Wickham 2016).

### Chemical Preparation and Synthesis

Standard experimental procedures were followed for synthesis; chemicals and solvents were from commonly used suppliers and were used without further purification. Silica gel 60 F254 analytical thin-layer chromatography plates were from Merck (Darmstadt, Germany), and they were visualized under UV light and/or with potassium permanganate stain. Chromatographic purifications were performed using Merck Geduran 60 silica (40–63 μm) or prepacked SNAP columns using a Biotage SP1 Purification system (Uppsala, Sweden). Microwave-assisted reactions were performed using a Biotage Initiator microwave synthesizer in sealed vials. Deuterated solvents were obtained from Cambridge Isotopes, Sigma-Aldrich, Goss Scientific Instruments Ltd., and Apollo Scientific Ltd. All ^1^H and ^13^C NMR spectra were recorded using a Bruker Avance 400 MHz spectrometer. All chemical shifts are given in ppm relative to the solvent peak, and coupling constants (*J*) are reported in Hz. Low-resolution (LR) mass spectrometry data (*m*/*z*) were obtained from an Agilent 6140 series quadrupole mass spectrometer with a multimode source attached to an Agilent 1200 series HPLC. Further synthetic routes and chemical characterization can be found in Supplementary A.

### Preparative High-Performance Liquid Chromatography (HPLC) Method

Preparative HPLC was carried out on a Waters HPLC comprising a Waters 2767 Sample Manager, a Waters 2545 Binary Gradient Module, a Waters Systems Fluidics Organizer, a Waters 515 ACD pump, and a Waters 2998 photodiode array detector, using a Waters XBridge Prep OBD C18, 5 μm, a 19 mm × 50 mm i.d. column, and a flow rate of 20 mL/min. The general method that may be used to purify compounds is acidic reverse-phase HPLC (water/acetonitrile/0.1% trifluoroacetic acid) using a standard gradient of 5% acetonitrile/95% water to 100% acetonitrile or basic reverse-phase HPLC (water/acetonitrile/0.01 M ammonia solution) using a standard gradient of 10% acetonitrile/90% water to 100% acetonitrile. UV detection, e.g., 254 nM, is used for the collection of fractions from HPLC. This description gives general methods, and variations in types of equipment, columns, mobile phase, detection wavelength, solvent gradient, and run time may also be used to purify compounds.

### Separation of Enantiomer

#### Preparation of (S)-(3-Chlorothiophen-2-yl)­(2-(3′-hydroxy-[1,1′-biphenyl]-2-yl)­pyrrolidin-1-yl)­methanone (ESC1002082)

This compound was obtained by separation of a racemic mixture using a Waters Investigator SFC. Separation was carried out on a Chiracel AD-H column 10 × 250 mm, using a flow rate of 10 mL/min, an injection volume of 100 μL (concentration 10 mg mL^–1^), and 13% methanol as a cosolvent. After repeated injections, the appropriate fractions were collected, and methanol was evaporated to provide the title compound as a solid.

^1^H NMR (100 °C, 400 MHz, DMSO-d6) ppm: 9.05 (1H,OH, s); 7.62 (d, *J* = 4.4 Hz, 1H); 7.37–7.27 (m, 2H); 7.26–7.15 (m, 2H); 7.06 (d, *J* = 7.3 Hz, 1H); 6.96 (d, *J* = 5.2 Hz, 1H); 6.78 (dd, *J* = 7.8, 1.8 Hz, 1H); 6.60 (br, 2H); 5.18–5.09 (m, 1H); 3.87–3.76 (m, 1H); 3.68–3.57 (m, 1H); 2.23–2.14 (m, 1H); 2.02–1.92 (m, 1H); 1.88–1.69 (m, 2H); LC-MS analytical method B, RT = 2.56 min, *m*/*z* 384.2 [M + H]^+^; chiral SFC (AD-H column, 5 mL/min, 13% MeOH), RT = 6.64 min, 99% ee.

## Results and Discussion

The primary screen of ∼350,000 drug-like compounds was carried out using the methods developed in Aitken et al.^21^ Those compounds exhibiting an inhibitory effect of ≥ 25% in the initial 10 μM enzyme inhibition assay were rescreened in the confirmatory assay with a further 458 compounds identified with a *Z*-score≥ 4. These assays were supplemented with an additional three potential false negatives, identified using a Bayesian model built from the primary screen and then tested for potency. A cross-site confirmation was performed comparing two institutes' results, and the resulting 458 hits were retested at 20 μM in duplicate using the primary assay. There were 115 compounds that showed activity across all assays. A review of the structures with the data was conducted, which led to a selection of 100 compounds for confirmatory LC-MS analysis. High priority was given to compounds and their structural analogues, where cross-program information indicated that they were not promiscuous. A qualified hit list (QHL) comprising 50 compounds was produced, which were split into structural related clusters. Overall, 26 structural clusters were identified, of which 16 were singleton clusters. The compounds were ranked within each cluster by their potency in the primary dose-response assay, resulting in cluster 6 and a singleton from cluster 11 appearing to be the most promising.

Cluster 6 compounds all contain a core imidazole benzamide as the key structural feature and was one of the largest clusters on the QHL. After further validation using our orthogonal assays, dose-response assays, and assessing physicochemical properties, a cluster of five molecules featuring this core imidazole benzamide and a singleton molecule was identified as good starting points for obtaining lead-like molecules, and the physicochemical properties of the hit compounds are shown in Table 1. Overall, these compounds are lead-like although most have lipophilicities that are higher than optimal for hits (e.g., log *D* > 4). In addition, as this target is within the central nervous system, the physicochemical properties lie within the idealized limits required for brain targeting.^31^

**Table 1:**
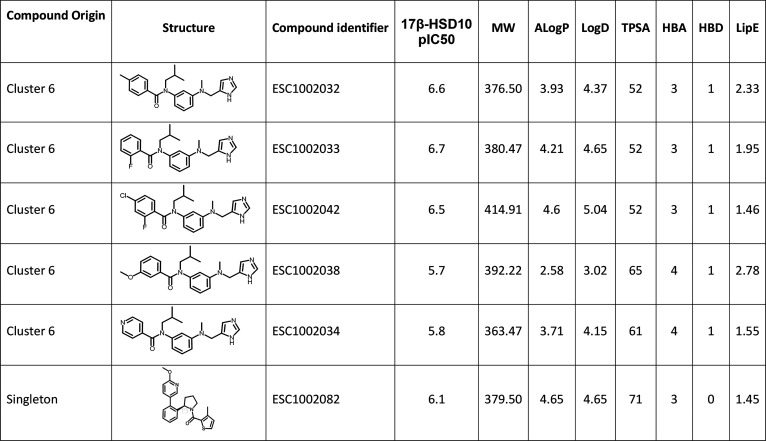
Primary Screening pIC_50_ Values for the Resynthesized HTS Hit Compounds with Physicochemical Properties (Calculated Using the ChemAxon Marvin Suite (http://www.chemaxon.com))^a^

a Here, MW, molecular weight (Da); ALogP, atom additive partition coefficient; LogD, distribution coefficient; TPSA, topological polar surface area; HBA, hydrogen-bond acceptor; HBD, hydrogen-bond donor; and LipE, lipophilic efficiency.

With regard to the singleton hit ESC1002082, this compound was identified as a moderately potent hit from the primary screen (Table 1), but as above, its physicochemical properties were consistent with a molecule capable of entering the central nervous system, although its lipophilicity was higher than optimally required and, consequently, its LipE was relatively low (Table 1). Details of the chiral synthetic route and preparation are detailed in Supplementary B.

### Mechanism of Action

Two compounds, ESC1002033 and ESC1002082, representative of the two unique clusters, were investigated for their mechanism of action against the substrates, NADH (Supplementary C, Figure S2) and AcetoAcetyl-CoA (AcAc) (Supplementary C, Figure S3). Both ESC1002033 and ESC1002082 inhibitors were tested as a 12-point serial dilution series with a maximal concentration of 2.5 μM. When investigating the effect of NADH concentration upon inhibition of 17β-HSD10 by the inhibitors, AcAc was kept in excess at 800 μM and NADH was diluted down from 150 μM. When investigating the effect of AcAc, it was diluted from 1000 μM with an excess of 100 μM NADH. Both inhibitors appear to have a similar mechanism of action. For NADH, with increasing concentration of the inhibitor, there is a decrease in Vmax (Figure SC1B) with no change in Km (Figure SC1C), which is characteristic of non-competitive (mixed) inhibition. This is also reflected by the convergence of the data to the left of the *y* axis on the Lineweaver–Burke plot (Figure SC1D). In contrast, with AcAc present, there was an increase in Km_app_ with increasing inhibitor concentrations (Figure SC2C), which is characteristic of competitive inhibition. However, these data are complicated by AcAc causing substrate inhibition at higher concentrations, thus not allowing it to be possible to accurately determine the Vmax (SC2B) as this effect is reflected by a decrease in the apparent Vmax upon increasing the inhibitor concentration. However, the data in Figure SC2D have been fitted for substrate inhibition (*Y* = *V*_max_ × *X*/(Km + *X* × (1 + *X*/*K*_i_))), and the diagnostic Lineweaver–Burke plot also supports the conclusion that inhibition is competitive with AcAc (Figure SC2D). In conclusion, the data suggest that both ESC1002033 and ESC1002082 bind non-competitively with the cofactor NADH but competitively with the substate AcAc.

### Cluster 6 Chemical Modifications

The cluster 6 analogues identified by the primary screen were subsequently altered on the amide side of the molecule, and initial structure–activity relationships (SARs) within the hits suggested that both *ortho-* and/or *para-* substitution with an electron-withdrawing group was favorable for potency. Initial analogues focused on exploring this region further with a small number of additional aryl amides. In addition to these changes, initial SAR exploration around the other parts of the hit was also performed to establish some initial SAR around these regions. Full data and structures with predicted physicochemical properties are detailed in Table 2.

**Table 2:**
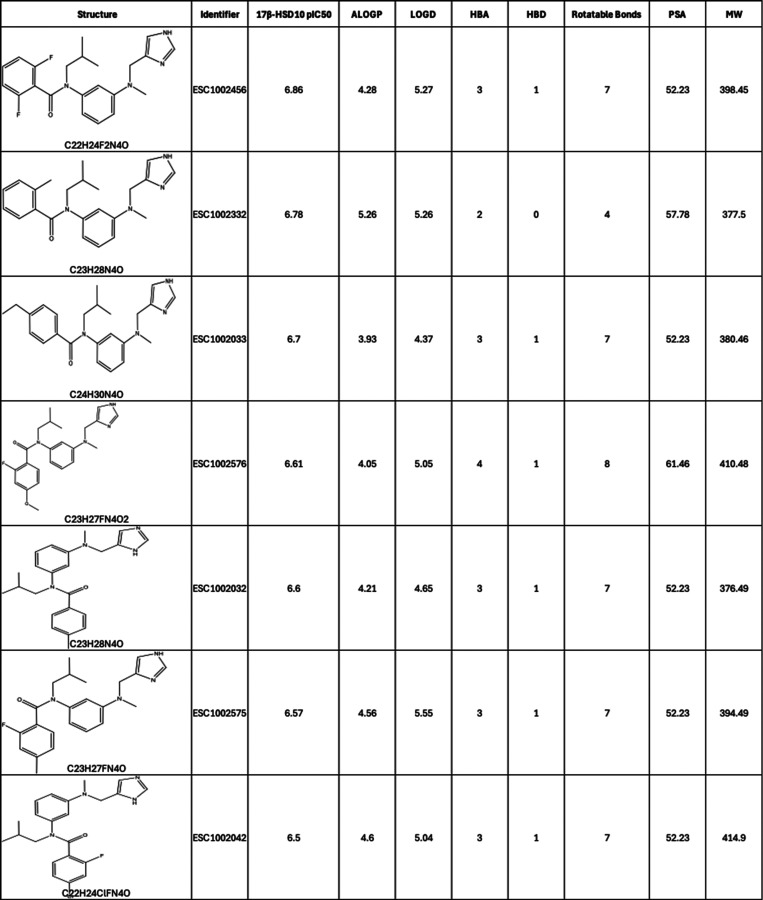

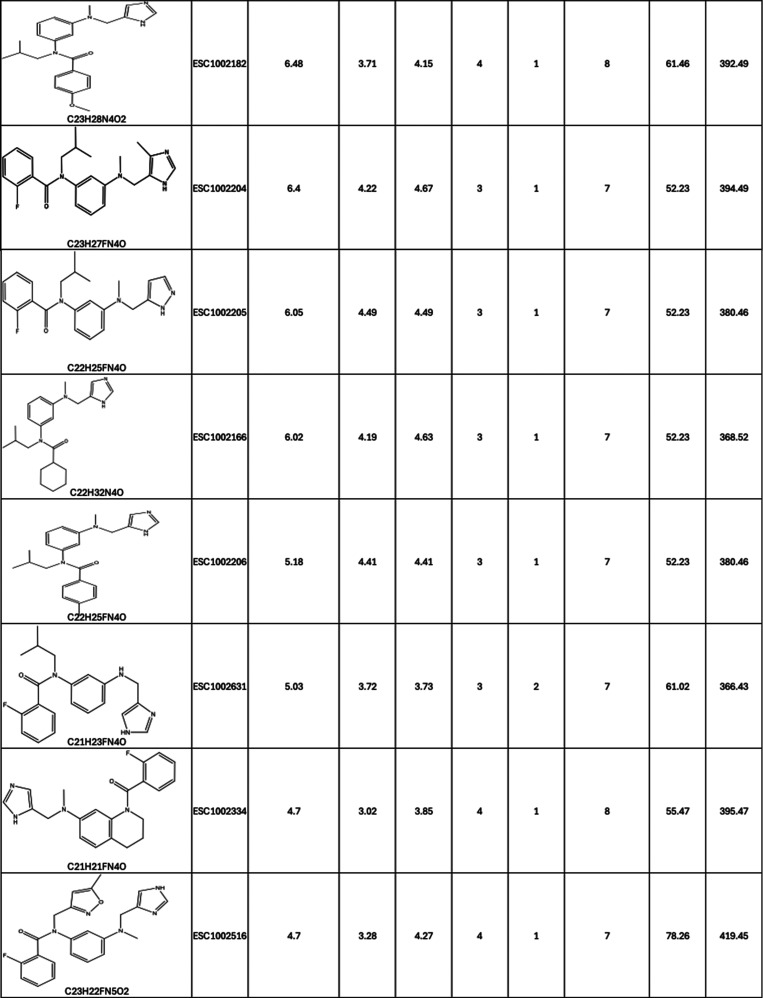

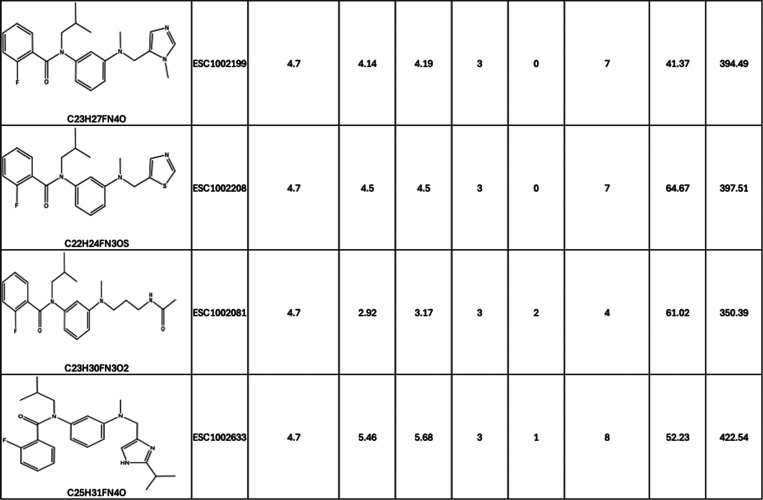
Primary Screening pIC_50_ Values for the Chemically Modified Cluster Hit Compounds with Physicochemical Properties (Calculated Using the ChemAxon Marvin Suite (http://www.chemaxon.com)) for the Resynthesized HTS Compounds^a^

a Here, MW, molecular weight (Da); ALogP, atom additive partition coefficient; LogD, distribution coefficient; HBA, hydrogen-bond acceptor; HBD, hydrogen-bond donor; and PSA, polar surface area.

Specifically, utilizing the primary screen and dose-response assays, results indicated that the replacement of the *iso*-propylmethyl results in some loss of potency with relatively subtle changes (Table 2). In addition to these subtle changes, SAR would suggest that heteroatoms (e.g., ESC1002334) and heteroaryl rings reduce potency (e.g., ESC1002516). Expansion of the SAR around the initial hits indicates that *ortho-* and *para-* substitution of the terminal phenyl ring with both electron-donating and -withdrawing groups is well tolerated. Disubstitution was also well tolerated and may give rise to a slightly improved inhibition of 17β-HSD10. Replacement of the terminal phenyl ring with an alkyl was also investigated, and although replacement with cyclohexyl (e.g., ESC1002166) resulted in a less potent compound, this compound may have improved physicochemical, drug metabolism, and pharmacokinetic properties. Extending the terminal phenyl ring outward with insertion of methylenes gave rise to losses in potency.

It was also established that the appropriate positioning of a H-bond donor affects 17β-HSD10 activity, shown by comparing ESC1002199 and ESC1002033, and ESC1002205 and ESC1002206 (Table 2). Furthermore, comparing the imidazole/pyrazole pair, ESC1002033 and ESC1002205, suggests that a H-bond acceptor (likely to be protonatable) also affects activity. When comparing ESC1002033 and ESC1002208, the thiophene and pyrazole will be un-ionized at physiological pH. Expansion of the terminal imidazole to a six-membered ring is also tolerated. It was also noted that when conformational constraints were implemented to lock the molecule into its bioactive conformation (ESC1002081 of Table 2), that potency was abolished.

Following the solution of the co-crystal structure of ESC1002033 complexed with17β-HSD10 (full details in Supplementary D), key interactions between 17β-HSD10 and ESC1002033 were found to include a H-bond from the imidazole to Gln162 and possibly also a H-bond to a well-ordered water molecule (Figure 1A). In addition, an edge-to-face interaction between the central aryl ring and Tyr168 of the catalytic triad is evident. With the crystal structure available, new opportunities presented themselves to develop further cluster analogues with increased potency. Specifically, a further set of analogues was prepared that maintained a H-bond donor in an equivalent position or increased the acidity of the donor itself. Specifically, the crystal structure revealed a small hydrophobic pocket close to the imidazole with a well-ordered water molecule present (Figure 1B). Thus, there was an opportunity to fill the pocket and potentially displace the water molecule to gain potency. In the initial heterocyclic replacement set, the 2-methylsubstituted imidazole, ESC1002203, was approximately 3-fold less potent than ESC1002033 (Table 2). Increasing the size of the 2-substituent to an *iso*-propyl, ESC1002633, resulted in further losses in potency. The slightly smaller and more *sp* ^2^-like *cyclo*-propyl was better tolerated than its *iso*-propyl congener (Table 2).

**Figure 1:**
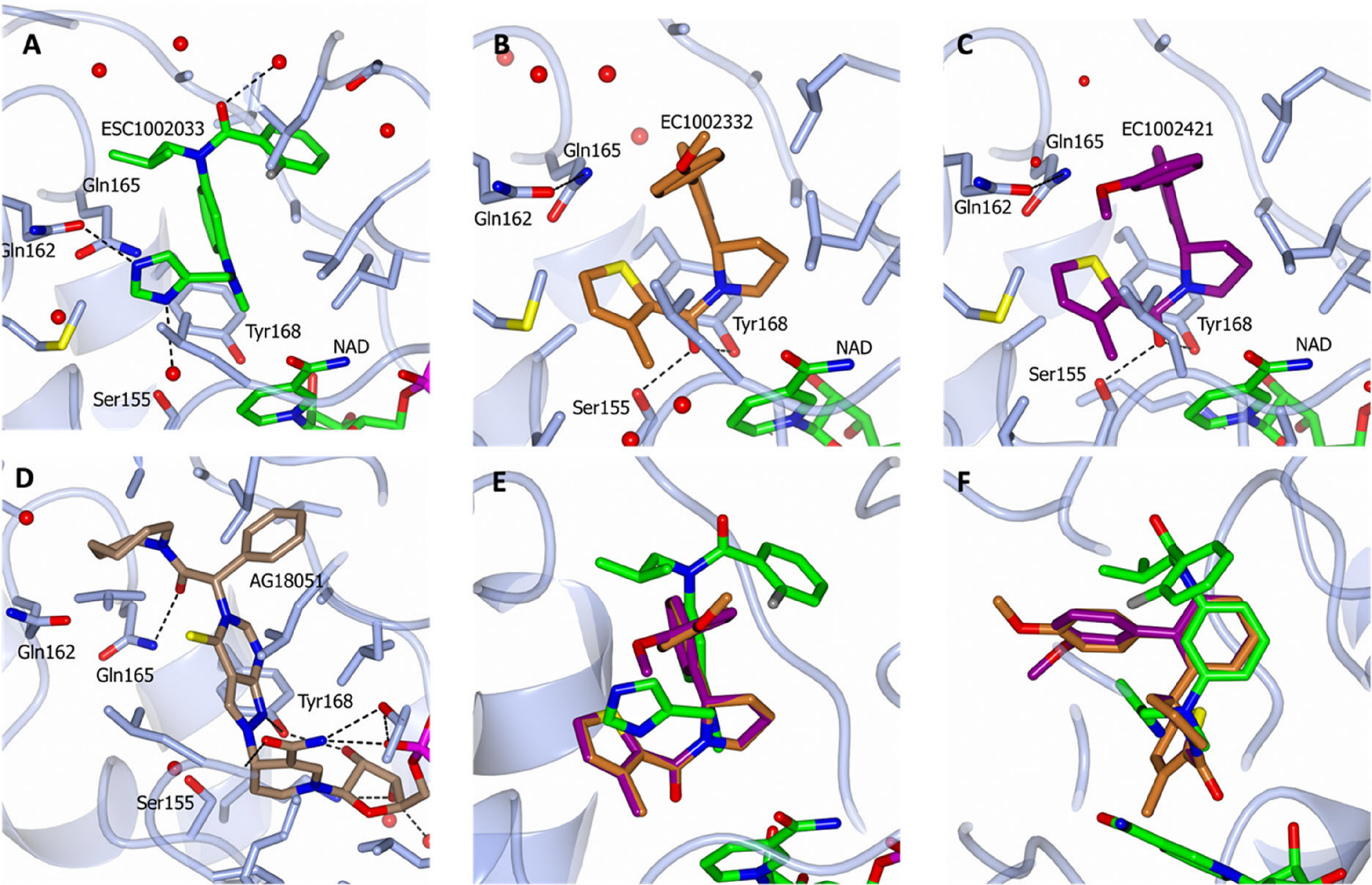
Key interactions between 17β-HSD10 and (A) ESC1002033, (B) ESC1002332, (C) ESC1002421, and (D) AG18051; (E and F) superimposition of our three bound ESC compounds demonstrating binding similarities and differences. The 17β-HSD10 protein is shown in light gray, and within the compound structures, nitrogen atoms are highlighted in blue, oxygen atoms are highlighted in red, sulfur atoms are highlighted in yellow, phosphorus atoms are highlighted in purple, water residues are indicated by red spheres, and dotted lines indicate hydrogen bonding between the protein/water/compounds.

The co-crystal structures of ESC1002033 and 17β-HSD10 (Figure 1A) also indicate that the fluorophenyl ring on ESC1002033 is almost perpendicular to the plane of the amide bond. Breaking the conjugation between these two systems is energetically unfavorable and is likely to be caused by steric effects from the central phenyl ring and the *ortho*-fluorine. The imidazole ring is at the center of a hydrogen-bond network. This side of the molecule is deeply buried and will have a low relative permittivity, and the effects of electrostatic interactions will be increased consequently. Another feature of this pocket is a well-ordered water molecule at the base of a subpocket, which is accessible through a vector from the imidazole ring.

Interaction of the ligand with the cofactor is mediated through the *N*-methyl group. Investigation of the small-molecule database showed that this was a common interaction and likely relies on the delta-positive hydrogen atoms of the methyl interacting with the delocalized electrons of the nicotinamide ring.

The crystal structure of ESC1002033 was superimposed with the only other reported crystal structure of a ligand bound to 17β-HSD10/AG18051 ^17^ (PDB 1U7T, Figure 1D). This ligand forms a covalent adduct with NADH, catalyzed by 17β-HSD10, resulting in suicide inhibition of the protein. Key interactions formed by the protein–ligand complex are a H-bond donor to Tyr168 and a H-bond to Gly162. Superposition of ESC1002033 onto the AG18051 crystal structure indicated that the Tyr168 H-bond was not engaged by the ESC1002033 ligand. Attempts to target the H-bond of Tyr168 resulted in losses in potency. Interestingly, the unsubstituted imidazole analogue ESC1002631, which was prepared as an intermediate, illustrates the importance of methyl substitution on the imidazole (Table 2).

Refinement statistics for the co-crystal complexes can be found in Supplementary D. Overall, both 2-substitution and 2,6-disubstitution resulted in improved potencies, resulting in the original hit ESC1002033 and ESC1002456 being identified as the most potent inhibitors within the series.

### Singleton Chemical Modifications

As indicated previously, the singleton hit ESC1002082 was identified as a moderately potent hit from the primary screen whose physicochemical properties were consistent with a molecule capable of entering the central nervous system, although its lipophilicity was higher than the optimum and its LipE was low (Table 1). The ESC1002082 compound contains a single chiral center and was registered as a racemate. Chiral separation was therefore required and is detailed in Supplementary B. The initial modifications were focused on ascertaining whether the stereochemical configuration of the chiral center is important for activity and if any SAR trends emerge with simple deletion analogues and amide changes.

The results of these initial changes indicate that the deletion of the nitrogen from the pyridone of ESC1002082 to the 4-methoxybenzene, ESC1002089, resulted in an approximate 3-fold improvement in activity, which was boosted further upon separation and testing of the single enantiomer, ESC1002332 (Table 3). Deletion of the methoxy moiety was not significantly detrimental to activity, ESC1002090. In contrast to the positive SAR observed with these modifications, changes to the amide side of the molecule were less well-tolerated. For example, replacement of the 2-methyl thiophene of ESC1002082 with 2-fluorobenzene resulted in a drop in potency and substitution with 4-methylbenzene resulted in complete ablation of activity. These changes suggested that the presence of a thiophene and *ortho*-substitution are potency drivers in this series. The potency enhancements observed with the deletion analogues focused initial SAR expansion efforts to aryl ring replacements, with the aim of further potency gains.

**Table 3:**
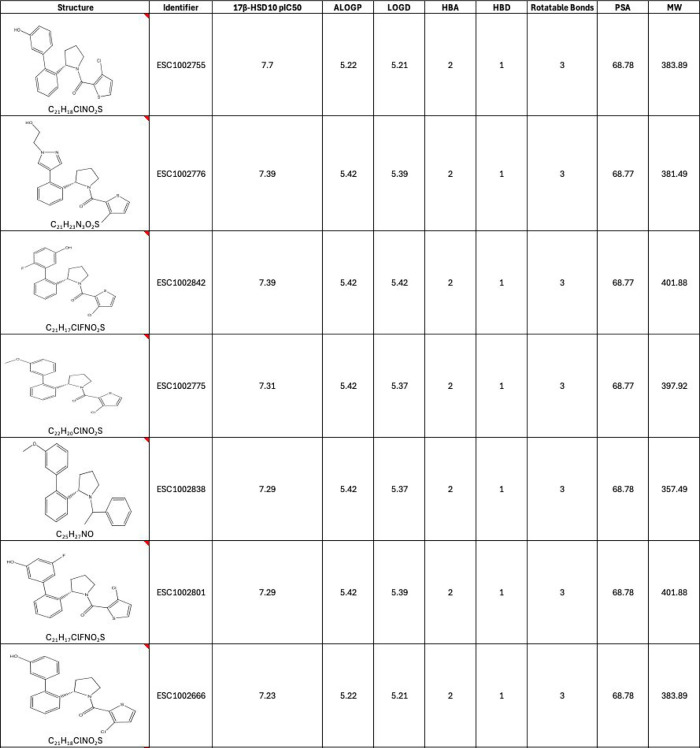

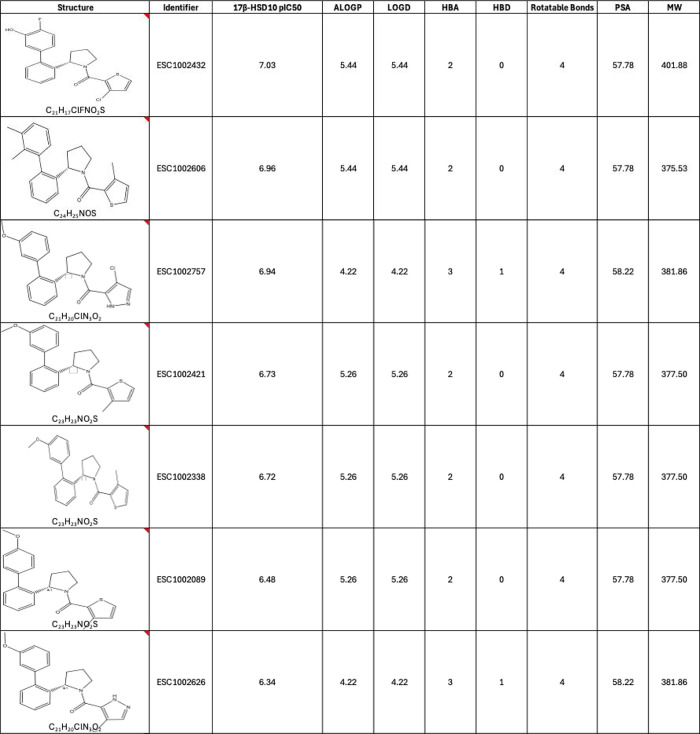

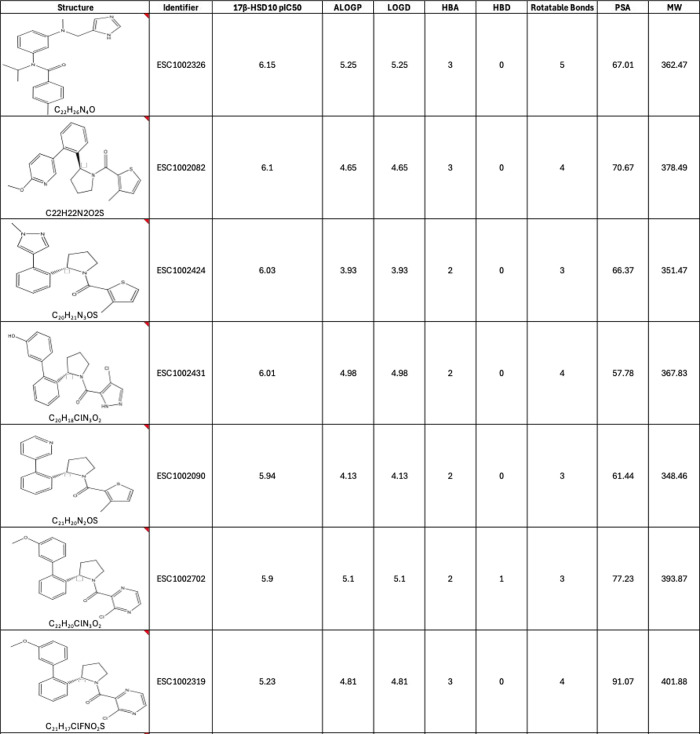

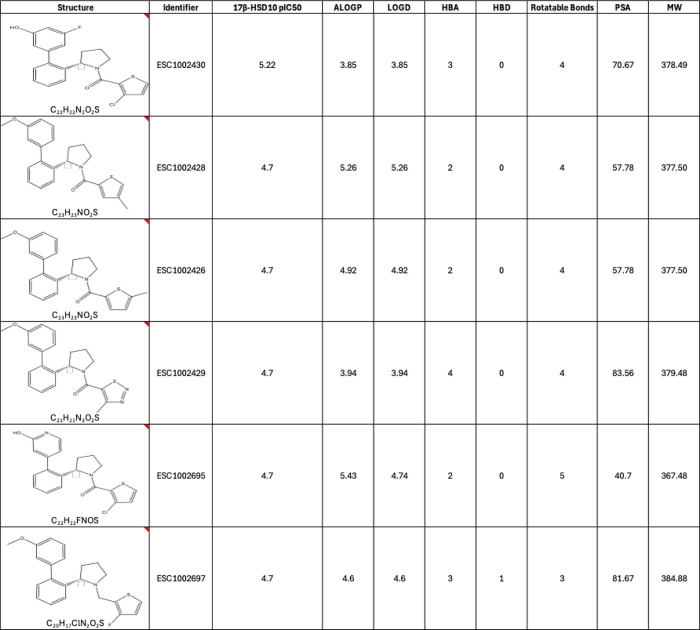
Primary Screening pIC_50_ Values for the Chemically Modified Singleton Hit Compound (ESC1002082) with Physicochemical Properties (Calculated Using the ChemAxon Marvin Suite (http://www.chemaxon.com)) for the Resynthesized HTS Compounds^a^

a Here, MW, molecular weight (Da); ALogP, atom additive partition coefficient; LogD, distribution coefficient; HBA, hydrogen-bond acceptor; HBD, hydrogen-bond donor; and PSA, polar surface area.

In addition, incorporation of more polar heterocycles and substituents was explored to reduce lipophilicity. A variety of substituents and substitution patterns were well tolerated with most compound modifications retaining activity levels within a log unit of the original hit, ESC1002082. Less favored groups are polar electron-withdrawing groups, e.g., the sulfones ESC1002322 and ESC1002319 (Table 3). On changing the position of the methoxy group of ESC1002089 around the benzene ring, it was found that the potency of the *ortho*-methoxy analogue was similar to ESC1002089 (*para*-methoxy), and the potency of the *meta*-methoxy analogue (ESC1002338) was greater than ESC1002089, exhibiting an IC_50_ of 191 nM. An analogue comprising both *ortho-*methoxy and *meta*-methoxy (ESC1002326) did not lead to additive effects (Table 3). Heterocyclic changes were also reasonably well tolerated. The pyrazole derivative ESC1002424 had comparable affinity to the initial hit (ESC1002082) but had improved physicochemical properties (ESC1002082 LipE 1.45 vs ESC1002424 LipE 2.1).

Having expanded the aryl ring SAR and identified ESC1002338 as a more potent 17β-HSD10 inhibitor, the next area of analogue expansion was around the aryl amide. SAR around the amide was found to be limited. Altering the position of the methyl substituent on the thiophene group resulted in a loss of activity, *c.f.* ESC1002338 vs ESC1002428 and ESC1002426 (Table 3). In addition, the type of substituent also influenced potency, with greater potencies achieved with small apolar substituents. Of note is the 3-chloro substituted thiophene, ESC1002432 (racemic) and ESC1002606 (chiral), which gave over 3-fold improvement in activity with an IC_50_ of approximately 100 nM.

Some simple heterocyclic changes reduced the potency of the resultant singleton: the bioisosteric equivalents of the thiophene, ESC1002429 and ESC1002430, were significantly less active (Table 3). Replacing the thiophenyl with 4-chloropyrazolyl (ESC1002626 (racemic) and ESC1002757 (chiral)) resulted in similar activity levels to ESC1002606, but it also had improved lipophilicity with a 1-unit improvement in LipE (ESC1002606 LipE 1.52 vs ESC1002757 LipE 2.76). Direct replacement of the five-membered heterocycle with its benzene isostere was not tolerated (ESC1002434), nor were cycloalkyl replacements. Finally, the importance of the carbonyl amide was confirmed with the synthesis of ESC1002695, which lost all activity in comparison to ESC1002431. Having identified some improved affinity and LipE, the distal aryl ring was revisited with these newly identified higher potency substituents with the aim of improving both affinity and physicochemical properties further.

It was shown that increasing the electron richness of the aromatic ring (by introduction of the methoxy groups) resulted in a small decrease in potency, as did further substitution in the *para* position. Lower lipophilicity heterocycles were tolerated but resulted in lower activity. More successful was demethylation to the corresponding phenol, which gave a significant improvement in potency in comparison to its methoxy counterpart and resulted in the identification of ESC1002755 with an IC_50_ of 19 nM—the most potent compound identified in the enzyme screening assay (Table 3). Interestingly, both bioisosteric equivalents of this compound—the pyridone (ESC1002697) and indazole (ESC1002702)—were less active. Fluorination of the aromatic ring was well tolerated; however, it resulted in no further potency improvement, but it may serve to protect the electron-rich aromatic ring from further oxidative metabolism via cytochrome P450s.

### X-ray Crystal Structure of 17β-HSD10 with Inhibitors

The binding site of these inhibitors, both the series and the singleton, is similar, being flanked by potentially interacting residues Gln 162, Gln 165, and Ser 155, with the base of this binding site formed by Tyr 168. It is worth noting that this inhibitor binding site does not overlap but is proximal to the cofactor NAD binding site. Additionally, the nicotinamide moiety points away from the inhibitor binding site, presumably toward the substrate binding site. Hence, as supported by the kinetics and biophysical analysis, these inhibitors bind to a novel allosteric site.

### X-ray Crystal Structure of ESC1002332

By co-crystallizing the singleton series with 17β-HSD10, the binding pocket can be utilized in targeted structural modifications to further improve potency and efficacy. The ligand ESC1002332 bound in the same site as occupied by ESC1002033 (Figure 1B,E,F), sharing some interactions, but others vary significantly (Figure 1B,E,F). A key shared feature is the edge/face interaction of the central aryl ring of the ligand with Tyr168 at the base of the active site pocket. The thiophene ring occupies a more buried space compared with the imidazole ring of ESC1002033, and both molecules have aromatic groups extending toward the solvent (Figure 1E,F). ESC1002332 also forms a direct hydrogen bond to Tyr168 (Figure 1B).

### X-ray Crystal Structure of ESC1002421

In addition to the singleton compound ESC1002332, compound ESC1002421 was also co-crystallized with 17β-HSD10, with the ligand-bound crystal structure of ESC1002421 illustrated in Figure 1C. Key interactions include an edge-to-face interaction with Tyr168, as in ESC1002033 and ESC1002332 (Figure 1E,F), but there is also an additional H-bond interaction from the amide carbonyl to the catalytic triad phenol of Tyr168 as found in ESC1002332 (Figure 1E,F). A notable difference in the two complexes is the course of the ligands as they emerge from the binding site toward the solvent. Both chemotypes superimpose well at the core phenyl but diverge as they extend toward the solvent (Figure 1C,E,F). Furthermore, the bound structure is consistent with the active enantiomer being in the *S-*configuration (Supplementary B). Refinement statistics for the co-crystal complexes can be found in Supplementary D.

### Cell-Based Assays

To test our inhibitors in a biological setting, we used three different assays.

#### Cell Viability Testing and Cytotoxicity Assays

LDH toxicity assays and Alamar blue toxicity assays were profiled in a stable HEK293 cell line overexpressing 17β-HSD10. There were no significant cytotoxicity side effects observed with the vast majority of compounds, with 5–10% cytotoxicity observed when dosed with a large concentration of 50 μM; data are shown in Table 4.

**Table 4:** Cytotoxicity Viability and Potency of Compounds in 17β-HSD10 HEK293 Cells

compound ID	LDH % toxicity (50 μM dose)^a^	Alamar Blue % viability (50 μM dose)^b^	cellular 17β-HSD10 activity mean pEC50^c^
ESC1002089	5.40 ± 0.05	82.00 ± 2.23	1.786
ESC1002162	6.84 ± 0.02	78.79 ± 0.91	1.128
ESC1002166	4.55 ± 0.02	78.89 ± 0.90	0.976
ESC1002169	4.55 ± 0.02	96.19 ± 0.82	2.960
ESC1002183	5.08 ± 0.003	78.96 ± 1.38	1.224
ESC1002182	4.73 ± 0.01	85.38 ± 3.61	1.673
ESC1002203	6.64 ± 0.03	82.98 ± 1.25	2.456
ESC1002204	5.05 ± 0.01	96.72 ± 3.61	1.362
ESC1002205	5.84 ± 0.01	82.52 ± 0.57	3.135
ESC1002206	5.24 ± 0.02	85.13 ± 0.67	2.261
ESC1002265	4.52 ± 0.03	80.52 ± 0.83	5.549
ESC1002321	5.98 ± 0.02	84.52 ± 1.94	1.531
ESC1002323	5.03 ± 0.02	96.19 ± 0.82	1.677
ESC1002324	5.04 ± 0.03	85.64 ± 1.11	8.643
ESC1002325	4.36 ± 0.04	89.12 ± 0.19	5.736
ESC1002326	5.05 ± 0.01	88.92 ± 1.41	5.605
ESC1002332	3.52 ± 0.01	87.68 ± 1.67	7.221
ESC1002340	5.33 ± 0.02	94.26 ± 1.07	1.688
ESC1002338	4.64 ± 0.01	82.98 ± 1.25	1.147
ESC1002339	4.39 ± 0.01	82.52 ± 0.57	1.853
ESC1002337	4.10 ± 0.02	85.13 ± 0.67	1.745
ESC1002342	4.76 ± 0.01	85.04 ± 0.42	6.84
ESC1002421	5.16 ± 0.01	102.63 ± 1.32	0.671
ESC1002423	5.40 ± 0.02	84.92 ± 0.61	1.824
ESC1002432	4.24 ± 0.02	80.61 ± 0.78	1.156
ESC1002456	4.78 ± 0.01	79.18 ± 1.21	0.141
ESC1002462	4.61 ± 0.03	81.74 ± 0.92	0.860
ESC1002575	4.78 ± 0.02	92.57 ± 0.47	0.367
ESC1002576	5.19 ± 0.01	79.14 ± 0.99	0.599
ESC1002597	4.98 ± 0.04	78.78 ± 1.72	0.511
ESC1002606	5.09 ± 0.02	77.95 ± 1.09	0.477
ESC1002664	4.07 ± 0.02	88.29 ± 2.32	2.560
ESC1002666	4.49 ± 0.02	79.27 ± 1.03	0.138
ESC1002699	4.29 ± 0.01	82.65 ± 0.80	1.152
ESC1002700	5.93 ± 0.01	86.84 ± 0.67	1.881
ESC1002755	5.47 ± 0.04	85.34 ± 2.01	0.028
ESC1002757	5.03 ± 0.02	89.52 ± 0.84	0.266
ESC1002767	5.14 ± 0.02	79.22 ± 0.69	0.873
ESC1002769	5.84 ± 0.03	80.48 ± 1.12	0.713
ESC1002775	2.54 ± 0.004	81.53 ± 0.56	0.449
ESC1002776	20.00 ± 0.11	65.58 ± 1.67	0.228
ESC1002788	5.70 ± 0.02	80.51 ± 1.38	0.351
ESC1002799	5.51 ± 0.03	93.20 ± 0.43	0.170
ESC1002801	5.50 ± 0.01	82.55 ± 0.62	0.050
ESC1002838	5.58 ± 0.03	94.28 ± 1.31	0.232
ESC1002840	8.01 ± 0.02	81.86 ± 0.39	0.879
ESC1002842	5.90 ± 0.004	82.44 ± 0.32	0.040

a Percentage cytotoxicity of HEK293 cells overexpressing 17β-HSD10 treated with 50 μM compound as measured by an LDH assay (*n* = 3 ± SD).

b Percentage of viable 17β-HSD10 HEK293 cells treated with a 50 μM compound as measured by the Alamar Blue assay (*n* = 3 ± SD).

c Cellular 17β-HSD10 pEC50 measured in 17β-HSD10 overexpressing HEK293 cells via the CHANA fluorescence assay.

### Enzyme Activity Cell-Based Assay

A selection of compounds from the two series were profiled in a stable HEK 293 cell line overexpressing 17β-HSD10. Utilizing our fluorogenic probe–(−)­CHANA,^27^ a substrate of 17β-HSD10, allows the oxidative activity of 17β-HSD10 to be monitored in living cells. Cellular profiling of the compounds indicated a good correlation between the biochemical and cellular assays, with most compounds falling within 10-fold of the biochemical assay, indicating that cellular drop-off is not an issue in either series. Furthermore, several of the compounds were found to be very potent inhibitors of cellular 17β-HSD10 activity, having an EC50 of <100 nM. ESC1002755, the most potent, had an EC50 of 28 nM. Full results for cell viability, toxicity, and cell-based activity are shown in Table 4.

### *ADME Profiling*

Initial *in vitro* mouse hepatocyte stability and PPB experiments were undertaken on some of the most potent exemplars from the representative series, and their results are summarized in Table 5. All of the compounds were found to be either moderately or highly metabolized in mouse hepatocytes. The cluster exemplars appear more stable in comparison with the singleton exemplars.

**Table 5:** Initial Mouse Hepatocyte Stability and PPB Experiments^a^

compound ID	mean 17β-HSD10 pIC50	cellular 17β-HSD10 activity mean pEC50	*k*	ClmL/min/gliver	*T*_1/2_(mins)	Heps category	mouse PPB (% free)
ESC1002755	7.73	0.028	0.22	>50	3.1	high	
ESC1002842	7.46	0.040	0.17	>50	4.0	high	3.4
ESC1002757	6.98	0.266	0.13	>50	5.3	high	
ESC1002456	6.86	0.141	0.018	8.6	38.5	high	8.9
ESC1002799	6.96	0.170	0.046	21.9	15.2	high	9.9
ESC1002769	6.77	0.713	0.15	>50	4.7	high	
ESC1002033	6.7		0.013	6.2	53.8	mod	
ESC1002575	6.57	0.367	0.012	5.8	57.2	mod	7.6
ESC1002204	6.4	1.362	0.11	>50	6.2	high	
ESC1002597	6.4	0.511	0.02	7.2	46.2	Mod	
AG18051	7.04^b^		0.11	>50	6.6	High	

a These experiments were undertaken on some of the best exemplars from the representative series with AG18051, a previously published 17β-HSD10 inhibitor.^7,17^ Here, *k* is the elimination constant, Cl is the clearance, *T*_1/2_ is the half-life, Heps is the mouse hepatocytes, and PPB is the plasma protein binding.

b From Kissinger et al.^17^

## Conclusions

Almost 350,000 compounds were screened in our high-throughput recombinant enzyme assay to identify inhibitors against 17β-HSD10, a therapeutic target in Alzheimer’s disease and cancer. Using our orthogonal and binding assays, as well as examining compound physicochemical properties, a QHL comprising 50 compounds was identified. These 50 were split into 26 structural clusters, of which 16 were singleton clusters. The compounds were ranked within each cluster by their potency in the primary dose-response assay, with cluster 6 and a singleton from cluster 11 (ESC1002082) appearing to be the most promising. Both sets of compounds were tolerated well in cellular assays, with little toxicity and good on-target potency in the −(−)­CHANA assay. Crucially, these compounds have different modes of action from the previously published competitive inhibitors, which provides greater specificity with regard to the NADH binding site.

An extensive range of analogues for both series was prepared and tested to explore the SAR further. Utilizing the cocrystal structures obtained for both series (ESC1002033, ESC1002421, and ESC1002332) with around 2Å resolution, significant improvements in potency were achieved and several sub-100 nM ligands were identified. The most potent of these is ESC1002755, with an IC_50_ of 19 nM, an overall 40-fold improvement in potency (Table 2). In addition to the significant potency gains, several ligands with improved physicochemical properties (lipophilicity) were also identified, such as ESC1002799 (LipE = 2.96) and ESC1002757 (LipE = 2.76), allowing blood–brain barrier permeability.

Overall, these are the most advanced and most potent drug-like 17β-HSD10 inhibitors published to date. With further investigation and preclinical pharmacokinetic optimization, these key-lead compounds have a real potential to become part of a novel therapeutic approach to treat Alzheimer’s disease. Furthermore, there is emerging evidence showing that increases in 17β-HSD10 activity have an important role in various hormone-dependent cancers, including breast and bone cancers, but especially castrate-resistant prostate cancer, which has a very poor outcome and no cure (reviewed in Vinklarova et al.^1^).

## Supplementary Material



## Notes

Chemical synthesis schemes, preparation methods, and analytical methods for the hit compounds (Supplementary A, pages 2–47); chiral synthetic routes for series and determination (Supplementary B, page 48); graphs from the mechanism of action studies, steady-state kinetic titration curves (Supplementary C, pages 49–50); and data from crystallography and molecular modeling experiments (Supplementary D, pages 51–52) (PDF)
