# Supplementary material for: High-Throughput Screening of Potent Drug-like Molecules Targeting 17β-HSD10 for the Treatment of Alzheimer’s Disease and Cancer
Source: ACS Chem Biol. 2025 Jun 18;20(7):1544–59. doi: 10.1021/acschembio.5c00110 (PMC12281485; doi:10.1021/acschembio.5c00110)
Supplement: Supplementary file 1 [file cb5c00110_si_001.pdf]

Supplementary Information:

High throughput screening of potent drug-like molecules targeting 17β-HSD10 for the treatment of Alzheimer's disease and cancer.

High throughput screening of potent drug-like molecules targeting 17β-HSD10 for the treatment of Alzheimer's disease and cancer.

Aitken, Laura[1]; Baillie, Gemma[2]; Pannifer, Andrew[3]; Morrison, Angus[2]; Major, Louise L.;[4] Alphey, Magnus S.[4]; Sethi, Ritika[5]; Timmerman, Martin[6]; Robinson, John[2]; Riley, Jennifer[7]; Shishikura, Yoko[7]; Koekemoer, Lizbe[8]; Von Delft, Frank[8]; Rutjes, Helma[6]; Read, Kevin D.[7]; Jones, Philip S.[2]; McElroy, Stuart P.[2]; Smith, Terry K. [1,4]; Gunn-Moore, Frank J.[4*]

[1] University of St. Andrews, School of Chemistry, Biomolecular Sciences Building, North Haugh, St. Andrews KY16 9TF, United Kingdom

[2] BioAscent Discovery Ltd, Bo'Ness Road, Newhouse, Lanarkshire, ML1 5UH, United Kingdom

[3] Excientia, Oxford Science Park, The Schrödinger Building, Oxford OX4 4GE, United Kingdom

[4] Biomedical Science Research Complex, School of Biology, University of St. Andrews, North Haugh, St. Andrews KY16 9ST, United Kingdom

[5] Glaxo Smith Kline Biologicals, rue de l'Institut 89, 1330 Rixensart, Belgium

[6] European Lead Factory, Pivot Park – Banting Building (RE600), Kloosterstraat 9, 5349 AB, Oss, The Netherlands

[7] Wellcome Centre of Anti-infectives research, Drug Discovery Unit, School of Life Sciences, University of Dundee, Dow Street, Dundee DD1 5EH, United Kingdom

[8] University of Oxford, Centre for Medicines Discovery, Old Road Campus Research Building, United Kingdom

*Corresponding author; fjg1@st-andrews.ac.uk

INDEX

Supplementary A: Chemical synthesis, Pages 2-47

Supplementary B: Chiral synthetic route and determination, Page 48

Supplementary C: Mechanism of action, Pages 49-50

Supplementary D: Crystallography and Molecular Modelling, Pages 51-52

Supplementary A:

Synthesis of selected compounds

The chemical synthesis of an exemplified compound of formula (I) is outlined in Schemes S1 and S2, and the synthesis of an exemplified compound of formula (II) is outlined in Scheme S3.

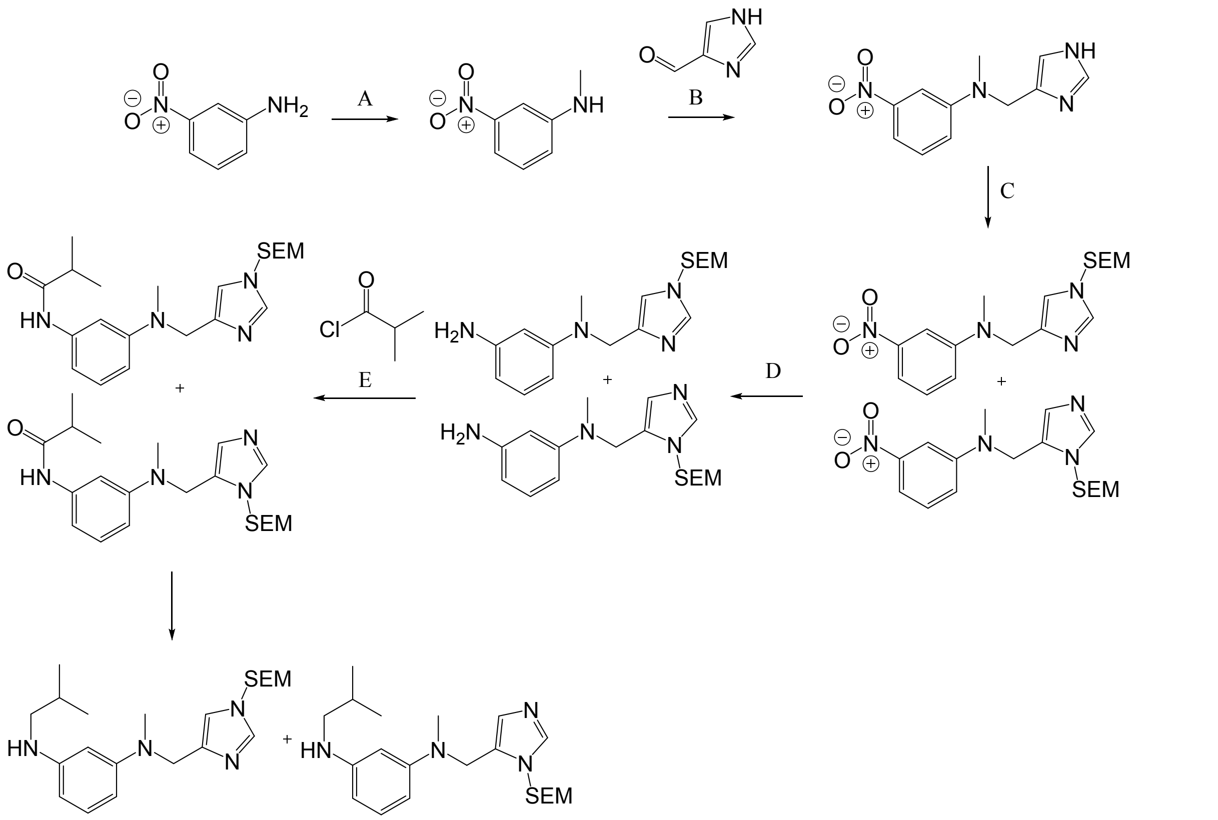

Scheme S1: Route to N1-isobutyl-N3-methyl-N3-((1-((2-(trimethylsilyl)ethoxy)methyl)-1H-imidazol-4-yl)methyl)benzene-1,3-diamine and N1-isobutyl-N3-methyl-N3-((1-((2-(trimethylsilyl)ethoxy)methyl)-1H-imidazol-5-yl)methyl)benzene-1,3-diamine 1:1 regioisomeric mixture. A: i) NaOMe, paraformaldehyde, MeOH, RT, 24h, ii) $NaBH_4$, 80°C, 2h; B: AcOH, $Na(OAc)_3BH$, DCM, RT, 72h; C: NaH, SEM-Cl, THF, 0°C - RT, 2h; D: H_2, Pd/C, MeOH, 30°C, 20bar; E: DIEA, DCM, 0°C - RT, 1h; F: i) BH_3.THF, THF, RT - 70°C, 92h, ii) MeOH, 70°C, 24h.

Synthesis of N-methyl-3-nitroaniline

3-nitroaniline (5 g, 36.2 mmol) was added to a suspension of NaOMe (95%, 10.29 g, 181 mmol) in MeOH (30 mL) and the resulting suspension stirred and then added to a stirring suspension of paraformaldehyde (1.52 g, 50.68 mmol) in methanol (40 mL). The reaction was stirred at room temperature for 18 hours then $NaBH_4$ (1.37 g, 36.2 mmol) was added and the reaction heated to reflux for 2 hours. The reaction mixture was allowed to cool to room

temperature then concentrated *in vacuo*. The mixture was partitioned between water (100 mL) and EtOAc (150 mL). The aqueous layer was separated and extracted with additional EtOAc (100ml). Organics were combined, washed with brine, dried over sodium sulphate, filtered and solvent was evaporated *in vacuo.* The crude product was purified by flash column chromatography (silica column, 0% to 100% DCM in *n*-heptane gradient) followed by concentration of the appropriate fractions *in vacuo* to afford N-methyl-3-nitro-aniline as an orange solid (4.84 g, 88 %).

^{1}H NMR (400 MHz, $CDCl_3$) δ 7.54 (dd, *J* = 1.51, 8.03 Hz, 1H), 7.41 (t, *J* = 2.26 Hz, 1H), 7.26 - 7.33 (m, 1H), 6.89 (dd, *J* = 2.01, 8.03 Hz, 1H), 4.10 (br. s., 1H), 2.93 (br. s., 3H).

Synthesis of N-((1H-imidazol-4-yl)methyl)-N-methyl-3-nitroaniline

N-methyl-3-nitro-aniline (4 g, 26.29 mmol) and 1H-imidazole-5-carbaldehyde (3.28 g, 34.18 mmol) were combined in DCM (75 mL) and acetic acid (2.26 mL, 39.43 mmol) was added. The suspension was stirred for 30 minutes then sodium triacetoxyborohydride (11.14 g, 52.58 mmol) was added. Stirring was continued at room temperature for 72 hours. The reaction mixture was diluted with DCM (75 mL) and washed with NaOH (2M aq., 100 mL). The aqueous wash was extracted with DCM (2 x 100 mL) then organics were combined, washed with brine, dried over sodium sulphate, filtered and solvent was evaporated *in vacuo* to afford crude product as an orange oil. Purification by flash column chromatography (silica column, DCM 0 % to 10 % gradient of a solution of 10 % NH_4OH in MeOH) followed by concentration of the appropriate fractions *in vacuo* afforded N-((1H-imidazol-4-yl)methyl)-N-methyl-3-nitroaniline as an orange gum (4.19 g, 69 %).

^{1}H NMR (400 MHz, $CDCl_3$) δ 7.64 (d, *J* = 0.75 Hz, 1H), 7.57 (t, *J* = 2.38 Hz, 1H), 7.51 (dd, *J* = 1.51, 8.03 Hz, 1H), 7.30 (t, *J* = 8.16 Hz, 1H), 7.05 (dd, *J* = 2.38, 8.41 Hz, 1H), 6.85 (s, 1H), 4.57 (s, 2H), 3.11 (s, 3H).

Synthesis of N-methyl-3-nitro-N-((1-((2-(trimethylsilyl)ethoxy)methyl)-1H imidazol-4-yl)methyl)aniline and N-methyl-3-nitro-N-((1-((2-(trimethylsilyl)ethoxy)methyl)-1H-imidazol-5-yl)methyl)aniline 1:1 regioisomeric mixture

NaH (60 %, 1.26 g, 31.39 mmol) was suspended in THF (20 mL) placed under argon and cooled to 0°C. A solution of N-(1H-imidazol-5-ylmethyl)-N-methyl-3-nitro-aniline (6.08 g, 26.16 mmol) in THF (80 mL) was added slowly and the mixture stirred at 0°C for 45 minutes. 2-(Trimethylsilyl)ethoxymethyl chloride (6.95 mL, 39.24 mmol) was added and the reaction stirred at 0°C for 30 minutes and at room temperature for 2 hours. The reaction mixture was concentrated *in vacuo* to remove THF and the resulting suspension partitioned between EtOAc (150 mL) and NaOH (1 M, 100 mL). The aqueous layer was extracted with EtOAc (50 mL) then organics were combined, washed with brine, dried over sodium sulphate,

filtered and concentrated *in vacuo* to afford crude product as an orange solid. Purification by flash column chromatography (silica column, DCM 0 % to 10 % gradient of a solution of 10 % NH_4OH in MeOH) followed by evaporation of solvent from the appropriate fractions afforded a 1:1 regioisomeric mixture of the title compounds as an orange oil (6.02 g, 64 %).
LC-MS Analytical Method A: rt = 1.52, 1.58 min, *m/z* 363.2 $[M+H]^+$.

Synthesis of N^1-methyl-N^1-((1-((2-(trimethylsilyl)ethoxy)methyl)-1H-imidazol-4-yl)methyl)benzene-1,3-diamine and N^1-methyl-N^1-((1-((2-(trimethylsilyl)ethoxy)methyl)-1H-imidazol-5-yl)methyl)benzene-1,3-diamine 1:1 regioisomeric mixture

A solution of N-methyl-3-nitro-N-((1-((2-(trimethylsilyl)ethoxy)methyl)-1H-imidazol-4-yl)methyl)aniline and N-methyl-3-nitro-N-((1-((2-(trimethylsilyl)ethoxy)methyl)-1H-imidazol-5-yl)methyl)aniline 1:1 regioisomeric mixture (1.13 g, 3.12 mmol) in MeOH (100ml) was passed through a H-Cube hydrogenation system equipped with a 10% Pd/C cartridge at 1mL/min flow rate, 30°C and a hydrogen pressure of 20 bar. The eluant was concentrated *in vacuo* to afford a brown oil. Purification by flash column chromatography (silica column, DCM 0 % to 10 % gradient of a solution of 10 % NH_4OH in MeOH) followed by evaporation of solvent from the appropriate fractions afforded the title compound regioisomeric mixture as a yellow oil (0.86 g, 83 %).
LC-MS Analytical Method A: rt = 1.29 min, *m/z* 333.2 $[M+H]^+$.

Synthesis of N-(3-(methyl((1-((2-(trimethylsilyl)ethoxy)methyl)-1H-imidazol-4-yl)methyl)amino)phenyl)isobutyramide and N-(3-(methyl((1-((2-(trimethylsilyl)ethoxy)methyl)-1H-imidazol-5-yl)methyl)amino)phenyl)isobutyramide 1:1 regioisomeric mixture

N^1-methyl-N^1-((1-((2-(trimethylsilyl)ethoxy)methyl)-1H-imidazol-4-yl)methyl)benzene-1,3-diamine and N^1-methyl-N^1-((1-((2-(trimethylsilyl)ethoxy)methyl)-1H-imidazol-5-yl)methyl)benzene-1,3-diamine 1:1 regioisomeric mixture (1.36 g, 4.08 mmol) was dissolved in DCM (20 mL) and DIEA (2.13 mL, 12.23 mmol) was added. The solution was cooled to 0°C, 2-methylpropanoyl chloride (0.56 mL, 5.3 mmol) was added and the reaction was stirred at 0°C for 1 hour. The reaction mixture was diluted with DCM (20 mL) and washed with water (20ml) saturated sodium bicarbonate solution (20 mL) and brine. Organics were dried over sodium sulphate, filtered and solvent was evaporated *in vacuo* to afford a brown oil. Purification by flash column chromatography (silica column, DCM 0 % to 10 % gradient of a solution of 10 % NH_4OH in MeOH) followed by evaporation of solvent from the appropriate fractions afforded the title compound regioisomeric mixture as a pale brown oil (1.1 g, 67 %).
LCMS Analytical Method A: rt = 1.49 min, *m/z* 403.2 $[M+H]^+$.

Synthesis of N[1]-isobutyl-N[3]-methyl-N[3]-((1-((2-(trimethylsilyl)ethoxy)methyl)-1H-imidazol-4-yl)methyl)benzene-1,3-diamine and N[1]-isobutyl-N[3]-methyl-N[3]-((1-((2-(trimethylsilyl)ethoxy)methyl)-1H-imidazol-5-yl)methyl)benzene-1,3-diamine 1:1 regioisomeric mixture.

N-(3-(methyl((1-((2-(trimethylsilyl)ethoxy)methyl)-1H-imidazol-4-yl)methyl)amino)phenyl)isobutyramide and N-(3-(methyl((1-((2-(trimethylsilyl)ethoxy)methyl)-1H-imidazol-5-yl)methyl)amino)phenyl)isobutyramide 1:1 regioisomeric mixture (1.1 g, 2.73 mmol) was dissolved in 2-MeTHF (7.5 mL) and borane THF complex solution (1 M, 7.5 mL, 7.50 mmol) was added slowly at room temperature. The reaction was heated to 70°C for 20 hours then additional borane THF complex solution (1 M, 7.5 mL, 7.50 mmol) was added and heating continued for 72 hours. MeOH (20 mL) was added and heating continued at 70°C for 24 hours. Solvent was evaporated *in vacuo* and the resulting brown oil loaded onto silica and purified by flash column chromatography (silica column, DCM 0 % to 10 % gradient of a solution of 10 % NH_4OH in MeOH). Evaporation of solvent from the appropriate fractions afforded the title compound regioisomeric mixture as a pale brown gum (0.68 g, 64 %).
LC-MS Analytical Method A: rt = 1.54 min, *m/z* 389.2 $[M+H]^+$.

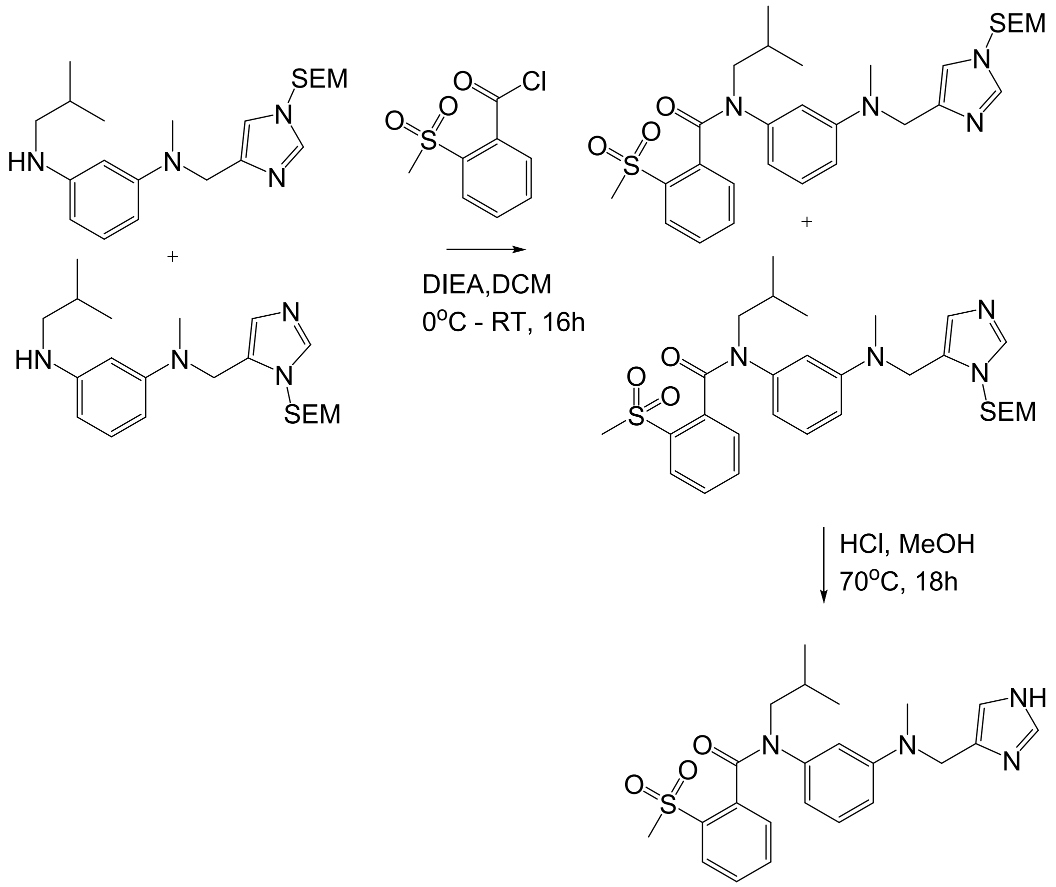

Scheme S2: General Procedure for amide formation with subsequent HCl deprotection.

Preparation of N-(3-(((1H-imidazol-4-yl)methyl)(methyl)amino)phenyl)-N-isobutyl-2-(methylsulfonyl)benzamide.

N-isobutyl-N-(3-(methyl((1-((2-(trimethylsilyl)ethoxy)methyl)-1H-imidazol-4-yl)methyl)amino)phenyl)-2-(methylsulfonyl)benzamide and N-isobutyl-N-(3-(methyl((1-((2-(trimethylsilyl)ethoxy)methyl)-1H-imidazol-5-yl)methyl)amino)phenyl)-2-(methylsulfonyl)benzamide 1:1 regioisomeric mixture.

N^1-isobutyl-N^3-methyl-N^3-((1-((2-(trimethylsilyl)ethoxy)methyl)-1H-imidazol-4-yl)methyl)benzene-1,3-diamine and N^1-isobutyl-N^3-methyl-N^3-((1-((2-(trimethylsilyl)ethoxy)methyl)-1H-imidazol-5-yl)methyl)benzene-1,3-diamine 1:1 regioisomeric mixture (47 mg, 0.12 mmol) was dissolved in DCM (1 mL) and DIEA (0.21 mL, 1.21 mmol. The solution was cooled to 0°C and 2-methylsulfonylbenzoyl chloride (52.89 mg, 0.24 mmol) was added. The reaction was stirred at 0°C for 30 minutes then 2-methylsulfonylbenzoyl chloride (52.89 mg, 0.24 mmol) was added and the reaction allowed to warm to room temperature and stirred for 16 hours. The reaction mixture was diluted with DCM (3 mL) and washed with HCl (0.5 M, 2 mL). Organics were filtered through a hydrophobic frit and solvent was evaporated *in vacuo* to afford crude product. Purification by flash column chromatography (silica column, DCM 0 % to 2.5 % gradient of a solution of 10 % NH_4OH in MeOH) followed by evaporation of solvent from the appropriate fractions afforded the title compound regioisomeric mixture (60 mg, 87 %).
LC-MS Analytical Method A: short method, rt = 1.56 min, *m/z* 571.0 $[M+H]^+$.

N-(3-(((1H-imidazol-4-yl)methyl)(methyl)amino)phenyl)-N-isobutyl-2-(methylsulfonyl)benzamide

N-isobutyl-N-(3-(methyl((1-((2-(trimethylsilyl)ethoxy)methyl)-1H-imidazol-4-yl)methyl)amino)phenyl)-2-(methylsulfonyl)benzamide and N-isobutyl-N-(3-(methyl((1-((2-(trimethylsilyl)ethoxy)methyl)-1H-imidazol-5-yl)methyl)amino)phenyl)-2-(methylsulfonyl)benzamide 1:1 regioisomeric mixture (60 mg, 0.11 mmol) was dissolved in MeOH (1 mL) and HCl (5 M, 0.5 mL) added. The reaction was heated to 70°C for 18 hours then concentrated *in vacuo*. The afforded residue was dissolved in DCM (2ml) and washed with saturated sodium bicarbonate solution (2ml). The aqueous wash was re-extracted with DCM (2 mL) then organics were combined, filtered through a hydrophobic frit and solvent was evaporated *in vacuo* to afford crude product, 54 mg.

Purification by preparative HPLC (basic) followed by evaporation of solvent and drying afforded N-(3-(((1H-imidazol-4-yl)methyl)(methyl)amino)phenyl)-N-isobutyl-2-(methylsulfonyl)benzamide (29 mg, 63 %).

^{1}H NMR @298K (400 MHz, DMSO-d_6) δ 11.81 (br. S., 1H), 7.85 (d, *J* = 7.78 Hz, 1H), 7.52 (s, 1H), 7.37 – 7.49 (m, 2H), 7.14 (d, *J* = 6.53 Hz, 1H), 6.94 (t, *J* = 8.03 Hz, 1H), 6.83 (br. S., 1H), 6.68 (s, 1H), 6.60 (d, *J* = 7.53 Hz, 1H), 6.44 – 6.53 (m, 1H), 4.23 (br. S., 2H), 3.95 (br. S., 1H), 3.41 – 3.56 (br. S., 1H), 3.37 (s, 3H), 2.70 – 2.85 (m, 3H), 1.68 – 1.83 (m, 1H), 0.92 (br. S., 6H).
High T NMR obtained at 373K to confirm structure:
^{1}H NMR @373K (400 MHz, DMSO-d_6) δ 11.52 (br. S., 1H), 7.87 (d, *J* = 7.28 Hz, 1H), 7.35 – 7.54 (m, 3H), 7.16 (br. S., 1H), 6.95 (br. S., 1H), 6.84 (s, 1H), 6.46 – 6.74 (m, 3H), 4.25 (br. S., 2H), 3.73 (br. S., 2H), 3.30 (s, 3H), 2.81 (br. S., 3H), 1.91 (br. S., 1H), 0.80 – 1.08 (m, 6H).
LC-MS Analytical Method B: rt = 1.69 min, *m/z* 441.2 $[M+H]^+$.

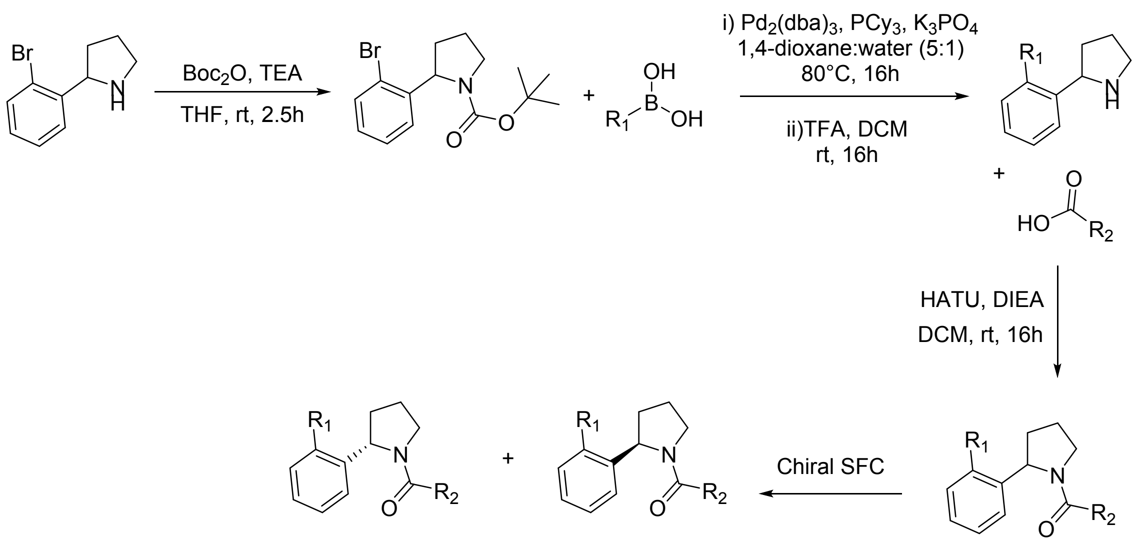

Scheme S3: General route towards pyrrolidine compounds

<u>Preparation of tert-butyl 2-(2-bromophenyl)pyrrolidine-1-carboxylate</u>

To a solution of 2-(2-bromophenyl)pyrrolidine (1500 mg, 6.63 mmol) in tetrahydrofuran (20 ml) was added di-tert-butyl dicarbonate (1737.37 mg, 7.96 mmol) and TEA (1.11 mL, 7.96 mmol) and the mixture was stirred at room temperature for 2.5h. The mixture was reduced and charged on a 25 g silica gel cartridge. The compound was purified by flash column chromatography using heptane:EtOAc (0-100%) gradient elution. The desired fractions were reduced and collected to afford an oil 2.16 g (100 %) of tert-butyl 2-(2-bromophenyl)pyrrolidine-1-carboxylate.
^{1}H NMR (273K, 400 MHz, $CDCl_3$) δ: 7.52 (dd, *J*= 8.0, 1.2 Hz, 1H), 7.29-7.22 (m, 1H), 7.18-7.04 (m, 2H), 5.25-5.20 (m, 0.3H), 5.12 (dd, *J*= 7.9, 4.1 Hz, 0.7H), 3.73-3.59 (m, 1.8H), 3.57-3.48 (m, 0.2H), 2.46-2.29 (m, 1H), 1.94-1.74 (m, 3H), 1.53 (s, 3H), 1.18 (s, 6H).
LC-MS Analytical Method B, RT = 3.00 min, m/z 270.0, 272.0 $[M+H]^+$

Preparation of 2-(3'-methoxy-[1,1'-biphenyl]-2-yl)pyrrolidine

To a microwave vial were added tert-butyl 2-(2-bromophenyl)pyrrolidine-1-carboxylate (2440 mg, 7.48 mmol), (3-methoxyphenyl)boronic acid (1704.79 mg, 11.22 mmol), 1,4-dioxane (5 ml) and water (1 ml) then the mixture was bubbled with argon for ~5 min. A mixture of $Pd_2(dba)_3$ (172.6 mg, 0.18 mmol), PCy_3(126.8 mg, 0.45 mmol), and K_3PO_4 (1600 mg, 7.5 mmol), was added to the reaction mixture and the vessel was sealed before heating at 80°C overnight. The mixture was partitioned between water and EtOAc and extracted with EtOAc (3 x 25ml). The organic phases were dried over Na_2SO_4 and filtered through a plug of Celite before reducing it to an oil. The oil was diluted with dichloromethane (5 ml), and TFA (5 ml, 65.34 mmol) was added to the reaction mixture before stirring it at room temperature for 2h. The mixture was washed with water and reduced after drying it over Na_2SO_4. The crude mixture was charged on a 25g silica cartridge and purified using DCM:MeOH (0-10%) on an Isolera. The desired fraction were reduced to obtain a yellow oil, 2560 mg (93.2%) of 2-[2-(3-methoxyphenyl)phenyl]pyrrolidin-1-ium with a trace of residual trifluoroacetic acid.

^{1}H NMR (273K, 400 MHz, $CDCl_3$) δ: 10.25 (br, 1H); 8.95 (br, 1H); 7.70 (m, 1H); 7.31-7.27 (m, 4H); 7.26-7.22 (m, 1H); 6.93 (dd, *J*= 8.4, 2.6 Hz, 1H); 6.79-6.73 (m, 1H); 4.60-4.48 (m, 1H); 3.83 (s, 3H); 3.40 (m, 1H); 3.16-3.04 (m, 1H); 2.27-2.12 (m, 2H); 2.10-2.02 (m, 1H); 1.99-1.88 (m, 1H).

LC-MS Analytical Method B, RT = 1.58 min, m/z 254.0 $[M+H]^+$

Preparation of (3-chlorothiophen-2-yl)(2-(3'-methoxy-[1,1'-biphenyl]-2-yl)pyrrolidin-1-yl)methanone

To a vial were added 2-[2-(3-methoxyphenyl)phenyl]pyrrolidine (50.67 mg, 0.2 mmol), 3-chlorothiophene-2-carboxylic acid (42mg, 0.26 mmol), dichloromethane (4 ml), DIEA (0.09 ml, 0.5 mmol) and O-(7-azabenzotriazol-1-yl)-N,N,N',N'-tetramethyluronium hexafluorophosphate (114.07 mg, 0.3 mmol) then the mixture was stirred at room temperature overnight. The solvents were removed by centrifugal evaporation, then purified on preparative HPLC (acidic conditions). The desired fractions were collected and evaporated to give the title compound, 34.5 mg (9%).

^{1}H NMR (273K, 400 MHz, DMSO-d_6) δ: 7.64 (d, *J*= 4.8Hz, 1H); 7.40-7.30 (m, 3H); 7.27 (td, *J*= 7.3, 1.8Hz, 1H); 7.1 (d, *J*= 7.3Hz, 1H); 6.99-6.92 (m, 2H); 6.75 (br, 1H); 5.13 (dd, *J*= 7.5, 5.4Hz, 1H); 3.89-3.79 (m, 4H); 3.67-3.57 (m, 1H); 2.23-2.11 (m, 1H); 2.04-1.93 (m, 1H); 1.90-1.72 (m, 2H);

LC-MS Analytical Method B, RT = 2.71 min, m/z 398.2 $[M+H]^+$

Preparation of (3-chlorothiophen-2-yl)(2-(3'-hydroxy-[1,1'-biphenyl]-2-yl)pyrrolidin-1-yl)methanone

To a solution of (3-chlorothiophen-2-yl)(2-(3'-methoxy-[1,1'-biphenyl]-2-yl)pyrrolidin-1-yl)methanone (50 mg, 0.126 mmol) in DCM at -78°C was added 1 M BBr_3 in dichloromethane (0.09 ml). The mixture was left to slowly warm to room temperature, a further portion of 1 M BBr_3, 0.1 ml, was then added to the reaction mixture and stirred overnight. After 16h a further portion of 1 M BBr_3 (0.1 ml) was added and the mixture left to stir for a further 5h at room temperature. The product was quenched with 10 ml of 1M HCl and extracted with DCM (3x 20 ml). The organic layers were dried over a phase separator and reduced. The crude material was charged on a 10 g silica cartridge and purified flash column chromatography Heptane:EtOAc as a gradient elution. The desired fractions were reduced and dried to obtain the title compound as a white powder, 42 mg (85% yield).

^{1}H NMR (373K, 400 MHz, DMSO-d_6) δ: 9.05 (1H,OH, s); 7.62 (d, *J*= 4.4Hz, 1H); 7.37-7.27 (m, 2H); 7.26-7.15 (m, 2H); 7.06 (d, *J*= 7.3Hz, 1H); 6.96 (d, *J*= 5.2Hz, 1H); 6.78 (dd, *J*= 7.8, 1.8Hz, 1H); 6.60 (br, 2H); 5.18-5.09 (m, 1H); 3.87-3.76 (m, 1H); 3.68-3.57 (m, 1H); 2.23-2.14 (m, 1H); 2.02-1.92 (m, 1H); 1.88-1.69 (m, 2H);

LC-MS Analytical Method B, RT = 2.56 min, m/z 384.2 $[M+H]^+$

Separation of Enantiomer

Preparation of (S) -(3-chlorothiophen-2-yl)(2-(3'-hydroxy-[1,1'-biphenyl]-2-yl)pyrrolidin-1-yl)methanone

The compound was obtained by separation of a racemic mixture using a Waters Investigator supercritical fluid (SFC). Separation was carried out on a Chiracel AD-H column 10x250mm, using a flow rate of 10 ml/min, an injection volume 100 microlitres (concentration 10 mg/mL) and 13% methanol as a co-solvent. After repeated injections the appropriate fractions were collected, methanol was evaporated to provide the title compound as a solid.

^{1}H NMR (373K, 400 MHz, DMSO-d_6) δ: 9.05 (1H,OH, s); 7.62 (d, *J*= 4.4Hz, 1H); 7.37-7.27 (m, 2H); 7.26-7.15 (m, 2H); 7.06 (d, *J*= 7.3Hz, 1H); 6.96 (d, *J*= 5.2Hz, 1H); 6.78 (dd, *J*= 7.8, 1.8Hz, 1H); 6.60 (br, 2H); 5.18-5.09 (m, 1H); 3.87-3.76 (m, 1H); 3.68-3.57 (m, 1H); 2.23-2.14 (m, 1H); 2.02-1.92 (m, 1H); 1.88-1.69 (m, 2H);

LC-MS Analytical Method B, RT = 2.56 min, m/z 384.2 $[M+H]^+$

Chiral SFC (AD-H column, 5 ml/min, 13% MeOH), RT = 6.64 min, 99% ee

Synthetic procedures for compounds referred to in Table 6

Procedure 1 (ESC1002019)

HCl (4M in dioxane, 1 mL) was added to tert-butyl 4-(2-carbamoyl-1-propyl-indol-5-yl)pyrazole-1-carboxylate (34 mg, 0.09 mmol) and the resulting suspension stirred for 18 hours (LCMS). Solvent was evaporated under reduced pressure and the resulting residue partitioned

between EtOAc (20ml) and saturated sodium bicarbonate solution (20ml). Organics were washed with brine and solvent was evaporated under reduced pressure to afford a pale brown solid (35mg). The solid was combined with product (12mg) for purification. Purification by prep. HPLC (basic) followed by evaporation of solvent from the appropriate fractions afforded product 5916A, 24 mg. 1H NMR (400 MHz, DMSO-d6) d 12.83 (br. s., 1H), 7.88 - 8.21 (m, 3H), 7.81 (s, 1H), 7.51 (s, 2H), 7.30 (br. s., 1H), 7.09 (s, 1H), 4.53 (t, J = 7.15 Hz, 2H), 1.62 - 1.78 (m, 2H), 0.80 (t, J = 7.40 Hz, 3H).
COSY NMR and 1H NMR in MeOD solvent both consistent with desired product.
LCMS long method, rt = 1.91 min, m/z [M+H]+ 269.0.

Procedure 2 (ESC1002024)
tert-butyl 4-(4,4,5,5-tetramethyl-1,3,2-dioxaborolan-2-yl)pyrazole-1-carboxylate (49.59 mg, 0.17 mmol) was charged to a microwave vial with CatKit ('dtbpf' 78mg comprising 0.05 equiv. $Pd(OAc)_2$, 0.1 equiv. dtbpf, 3 equiv. K_3PO_4). A solution of 2-bromo-5-(2,3,4-trimethoxyphenyl)thiophene (37 mg, 0.11 mmol) in dioxane (1.0 mL) was added followed by water (0.2 ml). The vial was sealed, placed under argon and the reaction mixture de-gassed with a flow of argon for 10 minutes. The reaction was heated to 80°C for 16 hours (LCMS). The reaction mixture was allowed to cool and diluted with EtOAc (10 mL) and water (10 mL). Organics were washed with water (2 x 10 mL) and brine, dried over sodium sulphate, filtered and solvent was evaporated under reduced pressure to afford crude product (40 mg). The crude product was dissolved in HCl (4M in dioxane, 2 mL) and stirred at room temperature for 5 hours. The solvent was evaporated under reduced pressure and the resulting residue dissolved in EtOAc (10 mL) and washed with saturated sodium bicarbonate solution (10ml). Organics were dried over sodium sulphate, filtered and solvent was evaporated under reduced pressure to afford crude product (38 mg). Purification by prep. HPLC (basic) followed by evaporation of solvent from the appropriate fractions afforded product as a white solid, 16 mg (45%) of 4-[5-(2,3,4-trimethoxyphenyl)-2-thienyl]-1H-pyrazole. 1H NMR (400 MHz, DMSO-d6) d 12.99 (br. s., 1H), 7.93 (br. s., 2H), 7.32 - 7.50 (m, 2H), 7.15 (d, J = 3.76 Hz, 1H), 6.88 (d, J = 9.04 Hz, 1H), 3.73 - 3.91 (m, 9H). COSY NMR consistent with product.
LCMS long method, rt = 2.36 min, m/z [M+H]+ 317.2.

Procedure 3 (ESC1002032)
tert-butyl 5-[[3-(diisobutylamino)-N-methyl-anilino]methyl]imidazole-1-carboxylate (37 mg, 0.09 mmol) was dissolved in HCl (4M in dioxane, 2 mL) and the reaction stirred at room temperature for 4 hours (LCMS). The reaction mixture was concentrated under reduced pressure then the residue dissolved in EtOAc (10 mL) and washed with saturated sodium bicarbonate solution (10 mL). Organics were dried over sodium sulphate, filtered and solvent

was evaporated under reduced pressure to afford crude product (28 mg). Purification by prep. HPLC (basic) followed by evaporation of solvent from the appropriate fractions afforded product as an off-white solid, 12 mg (42.8%) of N3-(1H-imidazol-5-ylmethyl)-N1,N1-diisobutyl-N3-methyl-benzene-1,3-diamine. NMR @ 298K: 1H NMR (400 MHz, DMSO-d6) d 11.84 (br. s., 1H), 7.54 (s, 1H), 7.11 (d, J = 8.03 Hz, 2H), 6.87 - 7.04 (m, 3H), 6.73 (s, 1H), 6.48 - 6.62 (m, 2H), 6.24 (d, J = 7.53 Hz, 1H), 4.20 - 4.39 (m, 2H), 3.65 (d, J = 7.28 Hz, 2H), 2.74 - 2.96 (m, 3H), 2.21 (s, 3H), 1.63 - 1.84 (m, 1H), 0.86 (d, J = 6.53 Hz, 6H). COSY NMR consistent with product. NMR @ 373K: 1H NMR (400 MHz, DMSO-d6) d 11.38 - 11.82 (br. s., 1H), 7.48 (s, 1H), 7.13 (d, J = 8.03 Hz, 2H), 6.89 - 7.03 (m, 3H), 6.62 - 6.78 (m, 1H), 6.54 (m, 2H), 6.33 (d, J = 6.78 Hz, 1H), 4.28 (s, 2H), 3.66 (d, J = 7.28 Hz, 2H), 2.84 (s, 3H), 2.23 (s, 3H), 1.88 (s, 1H), 0.90 (d, J = 6.78 Hz, 6H). LCMS long method, rt = 1.81 min, m/z [M+H]+ 377.2.

<u>Procedure 4 (ESC1002033)</u>

tert-butyl 5-[[3-(isobutylamino)-N-methyl-anilino]methyl]imidazole-1-carboxylate (85%, 65 mg, 0.15 mmol), 2-fluorobenzoic acid (32.39 mg, 0.23 mmol) and O-(7-Azabenzotriazol-1-yl)-N,N,N',N'-tetramethyluronium hexafluorophosphate (87.9 mg, 0.23 mmol) were charged to a reaction tube and dichloromethane (2 mL) added followed by DIEA (0.07 ml, 0.39 mmol). The reaction was stirred at room temperature for 16 hours. The reaction mixture was diluted with DCM (5 mL) and washed with water (2 mL). Organics were filtered through a hydrophobic frit and solvent was evaporated under reduced pressure to afford crude product (134 mg). Purification by flash chromatography (silica column heptane 10% to 60% EtOAc gradient) followed by evaporation of solvent from the appropriate fractions afforded product 5923A (26 mg, Boc-protected). Further eluting the column with a solution of DCM:MeOH:NH_4OH (180:10:1) followed by evaporation of solvent from the appropriate fractions afforded product 5923C (6 mg, de-protected, LCMS). Product 5923A was dissolved in HCl (4M diox., 1 mL) and stirred at room temperature for 4 hours, when no protected material remained. Solvent was evaporated under reduced pressure then the resulting residue dissolved in EtOAc (10 mL) and washed with saturated sodium bicarbonate solution (10ml). Organics were dried over sodium sulphate, filtered and solvent was evaporated under reduced pressure to afford crude product 5923D (22 mg). Products 5923C and 5923D were combined for purification by prep. HPLC (basic). Evaporation of solvent from the appropriate fractions and drying under vacuum afforded product as a colourless glass, 20 mg (34.1%) of 2-fluoro-N-[3-[1H-imidazol-5-ylmethyl(methyl)amino]phenyl]-N-isobutyl-benzamide. 1H NMR @ 298K: 1H NMR (400 MHz, DMSO-d6) d 11.71 - 12.03 (m, 1H), 7.54 (s, 1H), 7.10 - 7.34 (m, 2H), 6.86 - 7.08 (m, 3H), 6.68 (s, 1H), 6.53 (br. s., 2H), 6.35 (d, J = 7.53 Hz, 1H), 4.17 - 4.39 (m, 2H), 3.66 (d, J = 7.28

Hz, 2H), 2.74 - 2.93 (m, 3H), 1.62 - 1.83 (m, 1H), 0.90 (d, J = 6.53 Hz, 6H). 19F NMR consistent with product. COSY NMR consistent with product. LCMS long method, rt = 1.63, 1.71 min (split peak due to pka of compound), m/z [M+H]+ 381.1.

1H NMR @ 373K: 1H NMR (400 MHz, DMSO-d6) d 11.29 - 11.81 (br. s., 1H), 7.48 (s, 1H), 7.13 - 7.30 (m, 2H), 6.92 - 7.06 (m, 3H), 6.68 (br. s., 1H), 6.53 - 6.62 (m, 2H), 6.41 (br. s., 1H), 4.27 (br. s., 2H), 3.66 (d, J = 7.03 Hz, 2H), 2.84 (br. s., 3H), 1.75 - 1.91 (m, 1H), 0.92 (d, J = 6.53 Hz, 6H). LCMS long method, rt = 1.63, 1.71 min (split peak due to pka) m/z [M+H]+ 381.0.

<u>ESC1002032</u>

Procedure 4 with 4-methylbenzoic acid (31.48 mg, 0.23 mmol) in place of 2-fluorobenzoic acid and additional 4-methylbenzoic acid (15 mg) O-(7-Azabenzotriazol-1-yl)-N,N,N',N'-tetramethyluronium hexafluorophosphate (44 mg) and DIEA (40 µl) added after 16 hours, with stirring continued for 5 days. The reaction mixture was diluted with DCM (5 mL) and washed with 0.5M HCl (aq., 5 mL). Crude product (258 mg). Flash chromatography (silica column, heptane 0% to 100% EtOAc gradient) afforded product 5922A (42 mg, boc-protected product, contains tetramethylurea impurity). 5922A was dissolved in HCl (4M in dioxane, 1 mL), stirred at room temperature for one hour and allowed to stand for 16 hours. Crude product (29 mg) was extracted as in procedure D. Purification by prep. HPLC (basic method) followed by evaporation of solvent from the appropriate fractions under reduced pressure afforded product as a white solid, 23 mg (39.6%) of N-[3-[1H-imidazol-5-ylmethyl(methyl)amino]phenyl]-N-isobutyl-4-methyl-benzamide. NMR @ 298K: 1H NMR (400 MHz, DMSO-d6) d 11.84 (br. s., 1H), 7.54 (s, 1H), 7.11 (d, J = 8.03 Hz, 2H), 6.87 - 7.04 (m, 3H), 6.73 (s, 1H), 6.48 - 6.62 (m, 2H), 6.24 (d, J = 7.53 Hz, 1H), 4.20 - 4.39 (m, 2H), 3.65 (d, J = 7.28 Hz, 2H), 2.74 - 2.96 (m, 3H), 2.21 (s, 3H), 1.63 - 1.84 (m, 1H), 0.86 (d, J = 6.53 Hz, 6H). COSY NMR consistent with product. NMR @ 373K: 1H NMR (400 MHz, DMSO-d6) d 11.38 - 11.82 (br. s., 1H), 7.48 (s, 1H), 7.13 (d, J = 8.03 Hz, 2H), 6.89 - 7.03 (m, 3H), 6.62 - 6.78 (m, 1H), 6.54 (m, 2H), 6.33 (d, J = 6.78 Hz, 1H), 4.28 (s, 2H), 3.66 (d, J = 7.28 Hz, 2H), 2.84 (s, 3H), 2.23 (s, 3H), 1.88 (s, 1H), 0.90 (d, J = 6.78 Hz, 6H). LCMS long method, rt = 1.81 min, m/z [M+H]+ 377.2

<u>Procedure 5 (ESC1002038)</u>

tert-butyl 5-[[3-(isobutylamino)-N-methyl-anilino]methyl]imidazole-1-carboxylate (65 mg, 0.18 mmol) was dissolved in DCM (1 mL) and DIEA (0.08 ml, 0.45 mmol) added. The solution was cooled to 0°C then a solution of 3-methoxybenzoyl chloride (46.4 mg, 0.27 mmol) in DCM (0.5 mL) was added. The reaction was allowed to warm to room temperature and stirred for 16 hours (LCMS). The reaction mixture was partitioned between DCM (5 mL) and HCl (aq. 0.5M, 5 mL) then organics were filtered through a hydrophobic frit and solvent was evaporated to afford crude intermediate product (121 mg). Purification by flash chromatography (silica

column, heptane 0% to 100% EtOAc gradient) followed by evaporation of solvent afforded two products, 5933A and 5933B. Product 5933A consisting of mainly a side product but also some desired boc-protected product present, product 5933B being mainly desired boc-protected product. Each intermediate product was separately dissolved in HCl (4M in dioxane, 2 mL) stirred at room temperature for one hour and allowed to stand for 16 hours. Solvent was evaporated from each under reduced pressure then each residue was dissolved in EtOAc (5 mL) and washed with saturated sodium bicarbonate solution (2 mL). Organics were dried over sodium sulphate, filtered and solvent was evaporated under reduced pressure to afford a yellow oil in each case. 5933A afforded 38 mg (LCMS) and 5933B afforded 26mg (LCMS). Both residues were purified by prep. HPLC (basic). Product fractions from each were combined and solvent was evaporated under reduced pressure to afford product as a pale yellow solid, 20 mg (28.1%) of N-[3-[1H-imidazol-5-ylmethyl(methyl)amino]phenyl]-N-isobutyl-3-methoxy-benzamide. 1H NMR (400 MHz, DMSO-d6) d 11.65 - 12.25 (m, 1H), 7.58 (s, 1H), 7.09 (t, J = 7.78 Hz, 1H), 6.97 (t, J = 7.91 Hz, 1H), 6.74 - 6.82 (m, 3H), 6.70 (s, 1H), 6.50 - 6.60 (m, 2H), 6.31 (d, J = 7.53 Hz, 1H), 4.29 (s, 2H), 3.67 (d, J = 7.28 Hz, 2H), 3.57 (s, 3H), 2.84 (s, 3H), 1.74 (td, J = 6.78, 13.55 Hz, 1H), 0.87 (d, J = 6.78 Hz, 6H). COSY NMR consistent with product.
LCMS long method, rt = 1.73 min, m/z [M+H]+ 393.2.

(ESC1002034)
Procedure 5 with pyridine-4-carbonyl chloride hydrochloride (48.42 mg, 0.27 mmol) in place of a solution of 3-methoxybenzoyl chloride. Crude intermediate product (64 mg, LCMS), mainly boc-protected desired product). The aqueous wash was basified with addition of NaOH then extracted with DCM (2 x 3 mL). Organics were combined, filtered through a hydrophobic frit and solvent was evaporated under reduced pressure to afford a second crude product (26mg, LCMS, mainly de-protected desired product). The first (boc-protected) crude product was isolated as a yellow oil (LCMS) as described in procedure E for the intermediate products. The two crude residues were purified by prep. HPLC (basic) to afford product as a pale yellow solid, 17 mg (25.8%) of N-[3-[1H-imidazol-5-ylmethyl(methyl)amino]phenyl]-N-isobutyl-pyridine-4-carboxamide. 1H NMR @ 298K: 1H NMR (400 MHz, DMSO-d6) d 11.86 (br. s., 1H), 8.39 (d, J = 4.27 Hz, 2H), 7.55 (s, 1H), 7.14 (d, J = 4.02 Hz, 2H), 6.96 (t, J = 7.65 Hz, 1H), 6.75 (br. s., 1H), 6.62 (br. s., 1H), 6.55 (d, J = 7.78 Hz, 1H), 6.31 (d, J = 6.53 Hz, 1H), 4.30 (br. s., 2H), 3.67 (d, J = 6.78 Hz, 2H), 2.88 (s, 3H), 1.61 - 1.84 (m, 1H), 0.89 (d, J = 6.78 Hz, 6H). COSY NMR consistent with product.
1H NMR @ 373K:
1H NMR (400 MHz, DMSO-d6) d 11.29 - 11.86 (br. s., 1H), 8.32 - 8.46 (m, 2H), 7.49 (s, 1H), 7.07 - 7.20 (m, 2H), 7.00 (t, J = 7.53 Hz, 1H), 6.72 (br. s., 1H), 6.55 - 6.64 (m, 2H), 6.37 (br.

s., 1H), 4.29 (br. s., 2H), 3.68 (d, J = 7.03 Hz, 2H), 2.87 (br. s., 3H), 1.79 - 1.97 (m, 1H), 0.92 (d, J = 6.78 Hz, 6H). LCMS long method, rt = 1.41, 1.51 (split peak due to compounds pka), m/z [M+H]+ 364.2.

ESC1002042

Procedure 5 with 4-chloro-2-fluoro-benzoyl chloride (52.49 mg, 0.27 mmol) in place of 3-methoxybenzoyl chloride. Crude intermediate product (114 mg). Purification by flash chromatography (silica column, heptane 0% to 50% EtOAc gradient) afforded two products, 5932A and 5932B. Product 5932A consisting of mainly a side product but also some desired boc-protected product present, product 5932B (LCMS) being mainly desired boc-protected product. Each intermediate product was isolated as a yellow oil following the methods described in procedure E. 5932A afforded 26 mg (LCMS) and 5932B afforded 33 mg (LCMS). Both residues were purified by prep. HPLC to afford product as a pale yellow solid, 28 mg (37.2%) of 4-chloro-2-fluoro-N-[3-[1H-imidazol-5-ylmethyl(methyl)amino]phenyl]-N-isobutyl-benzamide. 1H NMR (400 MHz, DMSO-d6) d 11.89 (br. s., 1H), 7.55 (d, J = 0.75 Hz, 1H), 7.19 - 7.32 (m, 2H), 7.10 (d, J = 8.28 Hz, 1H), 6.97 (t, J = 8.28 Hz, 1H), 6.74 (s, 1H), 6.57 (br. s., 2H), 6.34 (d, J = 7.53 Hz, 1H), 4.30 (s, 2H), 3.65 (d, J = 7.28 Hz, 2H), 2.87 (s, 3H), 1.61 - 1.82 (m, 1H), 0.89 (d, J = 6.53 Hz, 6H). 19F / COSY NMR consistent with product.
LCMS long method, rt = 1.86 min, m/z [M+H]+ 415.0.

Procedure 6 (ESC1002054)

Procedure 4 with tert-butyl 5-[(3-amino-N-methyl-anilino)methyl]imidazole-1-carboxylate (89%, 60 mg, 0.18 mmol), O-(7-Azabenzotriazol-1-yl)-N,N,N',N'-tetramethyluronium hexafluorophosphate (100.72 mg, 0.26 mmol), and 4-methylbenzoic acid (36.07 mg, 0.26 mmol). The reaction mixture was diluted with DCM (5ml) and washed with 0.5M HCl (aq., 2.5 mL) instead of water. Crude intermediate was isolated as a brown oil. The oil was dissolved in HCl (4M in dioxane, 1.5 mL) and stirred at room temperature for 2 hours (LCMS). Solvent was evaporated under reduced pressure and the resulting residue dissolved in DCM (5 mL) and washed with saturated sodium bicarbonate solution (5ml). Organics were filtered through a hydrophobic frit and solvent was evaporated under reduced pressure to afford crude product. Purification by prep. HPLC (basic) followed by evaporation of solvent from the appropriate fractions afforded product as a pale yellow solid, 18 mg (31.8%) of N-[3-[1H-imidazol-5-ylmethyl(methyl)amino]phenyl]-4-methyl-benzamide. 1H NMR (400 MHz, DMSO-d6) d 11.31 - 12.28 (br. s., 1H), 9.92 (s, 1H), 7.86 (d, J = 8.28 Hz, 2H), 7.54 (d, J = 0.75 Hz, 1H), 7.32 (d, J = 7.78 Hz, 2H), 7.26 (s, 1H), 7.04 - 7.16 (m, 2H), 6.87 (s, 1H), 6.54 (d, J = 7.78 Hz, 1H), 4.39 (s, 2H), 2.94 (s, 3H), 2.38 (s, 3H). LCMS long method, rt = 1.49, 1.59 min (split peak due to compound pka), m/z [M+H]+ 321.2.

ESC1002056

Procedure F with 2-fluorobenzoic acid (37.12 mg, 0.26 mmol) in place of 4-methylbenzoic acid. Product isolated as a pale yellow solid, 23 mg (40.2%) of 2-fluoro-N-[3-[1H-imidazol-5-ylmethyl(methyl)amino]phenyl]benzamide. 1H NMR (400 MHz, DMSO-d6) d 11.48 - 12.50 (br. s., 1H), 10.17 (s, 1H), 7.60 - 7.67 (m, 1H), 7.52 - 7.60 (m, 2H), 7.27 - 7.37 (m, 2H), 7.19 (s, 1H), 7.01 - 7.13 (m, 2H), 6.88 (s, 1H), 6.55 (dd, J = 1.25, 8.03 Hz, 1H), 4.38 (s, 2H), 2.94 (s, 3H). COSY NMR / 19F NMR consistent with product. LCMS long method, rt = 1.37 / 1.52 min, m/z [M+H]+ 325.2 (split peaks due to compound pka).

Procedure 7 (ESC1002065

N-benzyl-2-chloro-5-(morpholinomethyl)pyrimidin-4-amine (178 mg, 0.56 mmol), 4-amino-2-chloro-phenol (96.19 mg, 0.67 mmol), $NaHCO_3$ (234.52 mg, 2.79 mmol) and tetrahydrofuran (10 mL), were added to a flask and the mixture was stirred at 60°C overnight. The mixture was reduced, and partitioned between DCM and Water and further extracted with DCM (3 x 15 mL). The organic phases were dried over Na_2SO_4. The solvents were removed and further 4-amino-2-chloro-phenol (96.19 mg, 0.67 mmol) was added to the reaction mixture followed by dioxane (40 mL) and the mixture was heated to 100°C for 3h. The mixture was reduced and loaded on a 25 g silica gel cartridge. The compound was purified on an Isolera using Hep:EtOAc (0-100%). The desired fractions were collected, reduced and the oil was further purified by Prep HPLC (basic). The collected fractions were dried, diluted in EtOAc and washed with 2M NaOH solution (3x 25 mL). The organic layer was dried over Na_2SO_4 and the solvents were removed. 1H NMR (DMSO-d6): 8.80 (1H, OH, s), 7.80 (1H, Ar-H2, d, J=2.6Hz), 7.68 (1H, Ar-H3, s), 7.54 (1H, N-H4, t, J=6.1Hz), 7.39-7.27 (7H, Ar-H5, m), 6.81 (1H, Ar-H6, d, J=8.8Hz), 4.65 (2H, CH27, d,J= 6.1Hz), 3.57-3.47 (4H, CH28, m), 3.32 (2H, CH29, s), 2.38-2.27 (4H, CH210, m)

Procedure 8 (ESC1002078)

tert-butyl 5-[[3-(isopropylamino)-N-methyl-anilino]methyl]imidazole-1-carboxylate (45 mg, 0.13 mmol) was dissolved in DCM (2 mL) and DIEA (0.07 ml, 0.39 mmol) was added. The solution was cooled to 0°C then a solution of 2-fluorobenzoyl chloride (31.07 mg, 0.2 mmol) in DCM (0.5 mL) was added dropwise. The reaction was allowed to warm to room temperature and stirred for one hour, then allowed to stand for 16 hours. The reaction mixture was diluted with DCM (5 mL) and washed with saturated sodium bicarbonate solution (5 mL). Organics were filtered through a hydrophobic frit and solvent was evaporated under reduced pressure to afford crude intermediate as a yellow gum. The intermediate was dissolved in HCl (4M in dioxane, 1.5 mL) and stirred for 3 hours (LCMS). Solvent was evaporated under reduced

pressure to afford a yellow gum. Purification by prep. HPLC (basic) followed by evaporation of solvent from the appropriate fractions and drying under vacuum afforded product, 18 mg (37.6%) of 2-fluoro-N-[3-[1H-imidazol-5-ylmethyl(methyl)amino]phenyl]-N-isopropyl-benzamide. 1H NMR (400 MHz, DMSO-d6) d 11.68 - 12.48 (m, 1H), 7.63 (s, 1H), 7.08 - 7.25 (m, 2H), 6.88 - 7.02 (m, 3H), 6.66 (s, 1H), 6.57 (d, J = 8.28 Hz, 1H), 6.48 (br. s., 1H), 6.34 (d, J = 7.53 Hz, 1H), 4.77 - 4.96 (m, 1H), 4.31 (s, 2H), 2.86 (s, 3H), 1.09 (d, J = 6.78 Hz, 6H). 19F / COSY NMR consistent with product. LCMS long method, rt = 1.61 min, m/z [M+H]+ 367.2.

ESC1002080

Procedure 8 with 4-methylbenzoyl chloride (30.29 mg, 0.2 mmol) in place of 2-fluorobenzoyl chloride. Product isolated as 28 mg (59.1%) of N-[3-[1H-imidazol-5-ylmethyl(methyl)amino]phenyl]-N-isopropyl-4-methyl-benzamide. 1H NMR (400 MHz, DMSO-d6) d 11.85 - 12.81 (br. s., 1H), 7.70 (s, 1H), 7.10 (d, J = 7.78 Hz, 2H), 6.90 - 7.02 (m, 3H), 6.76 (s, 1H), 6.59 (dd, J = 2.01, 8.28 Hz, 1H), 6.50 (s, 1H), 6.28 (d, J = 7.78 Hz, 1H), 4.69 - 4.92 (m, 1H), 4.34 (s, 2H), 2.88 (s, 3H), 2.19 (s, 3H), 1.08 (d, J = 6.78 Hz, 6H). COSY NMR consistent with product. LCMS long method, rt = 1.68 min, m/z [M+H]+ 363.2.

Procedure 9 (ESC1002081)

(7-amino-3,4-dihydro-2H-quinolin-1-yl)-(2-fluorophenyl)methanone (35 mg, 0.13 mmol) and 1H-imidazole-5-carbaldehyde (14.93 mg, 0.16 mmol) were suspended in DCM (1.5 mL) and acetic acid (0.01 ml, 0.19 mmol) was added. The mixture was stirred for 15 minutes then sodium triacetoxyborohydride (54.89 mg, 0.26 mmol) was added. Stirring was continued at room temperature for 16 hours (LCMS). The reaction mixture was diluted with DCM (20 mL) and washed with NaOH (1M aq., 10 mL) and brine (10 mL). Organics were dried over sodium sulphate, filtered and solvent was evaporated under reduced pressure to afford crude product as an off-white gum (74 mg). Purification by prep. HPLC (basic) followed by evaporation of solvent from the appropriate fractions and drying under vacuum afforded product, 20 mg (44.1%) of (2-fluorophenyl)-[7-(1H-imidazol-5-ylmethylamino)-3,4-dihydro-2H-quinolin-1-yl]methanone. NMR at 298K very broad so high T NMR obtained at 373K. 1H NMR (373K, 400 MHz, DMSO-d6) d 7.78 (s, 1H), 7.34 - 7.47 (m, 2H), 7.17 - 7.24 (m, 1H), 7.11 (t, J = 9.16 Hz, 1H), 6.84 - 6.91 (m, 2H), 6.40 (dd, J = 2.38, 8.16 Hz, 1H), 6.32 (br. s., 1H), 3.87 (s, 2H), 3.70 (t, J = 6.27 Hz, 2H), 2.66 (t, J = 6.78 Hz, 2H), 1.84 - 1.98 (m, 2H). 19F and COSY NMR consistent with product. LCMS long method, rt = 1.55 min, m/z [M+H]+ 351.2.

Procedure 10 (ESC1002082)

[2-(2-bromophenyl)pyrrolidin-1-yl]-(3-methyl-2-thienyl)methanone (110 mg, 0.31 mmol) (6-methoxy-3-pyridyl)boronic acid (57.64 mg, 0.38 mmol) and 'first choice' catkit (158mg

consisting of $Pd_2(dba)_3$ 0.05 eqiuv., $P(cy)_3$ 0.2 equiv., and K_3PO_4 2 equiv.) were charged to a sealable vial. 1,4-Dioxane (1 mL) was added followed by water (0.2 mL). The vial was sealed and placed under argon then the solution was de-gassed with a flow of argon for 10 minutes. The reaction was heated to 80°C for 20 hours (LCMS t = 5 hours, LCMS t = 20 hours). The reaction mixture was diluted with EtOAc (5 mL) and water (2 mL) and filtered through celite. Organics were separated, washed with water (5ml) and brine, dried over sodium sulphate, filtered and solvent was evaporated under reduced pressure to afford crude product, 126mg. Purification by flash chromatography (silica column, heptane 0% to 100% gradient) followed by evaporation of solvent from the appropriate fractions under reduced pressure and drying under vacuum afforded product, 80 mg (67.3%) of [2-[2-(6-methoxy-3-pyridyl)phenyl]pyrrolidin-1-yl]-(3-methyl-2-thienyl)methanone. NMR at 298K very broad so high T NMR obtained: 1H NMR (373K, 400 MHz, DMSO-d6) d 8.01 (br. s., 1H), 7.52 (d, J = 7.03 Hz, 1H), 7.35 - 7.43 (m, 2H), 7.24 - 7.35 (m, 2H), 7.10 (d, J = 6.78 Hz, 1H), 6.80 - 6.88 (m, 2H), 5.02 (dd, J = 5.77, 7.53 Hz, 1H), 3.94 (s, 3H), 3.80 (td, J = 7.03, 11.04 Hz, 1H), 3.60 - 3.71 (m, 1H), 2.20 (dd, J = 7.65, 12.17 Hz, 1H), 2.10 (s, 3H), 1.98 (td, J = 6.05, 12.74 Hz, 1H), 1.85 (s, 1H), 1.69 - 1.79 (m, 1H). Spectra too broad at 298K to obtain COSY NMR.
LCMS long method, rt = 2.64 min, m/z [M+H]+ 379.2.

ESC1002161 and ESC1002162 were obtained by SFC chiral separation of ESC1002082.
ESC1002161, T = 373K: 1H NMR (400 MHz, DMSO-d6) d 8.01 (br. s., 1H), 7.53 (br. s., 1H), 7.36 - 7.44 (m, 2H), 7.24 - 7.35 (m, 2H), 7.10 (d, J = 7.53 Hz, 1H), 6.80 - 6.91 (m, 2H), 4.97 - 5.07 (m, 1H), 3.94 (s, 3H), 3.80 (td, J = 7.15, 11.04 Hz, 1H), 3.61 - 3.71 (m, 1H), 2.15 - 2.26 (m, 1H), 2.10 (s, 3H), 1.93 - 2.04 (m, 1H), 1.85 (s, 1H), 1.74 (d, J = 6.78 Hz, 1H).
ESC1002162, T = 373K: 1H NMR (400 MHz, DMSO-d6) d 8.01 (br. s., 1H), 7.52 (d, J = 6.53 Hz, 1H), 7.35 - 7.44 (m, 2H), 7.23 - 7.34 (m, 2H), 7.10 (d, J = 7.53 Hz, 1H), 6.78 - 6.90 (m, 2H), 4.95 - 5.08 (m, 1H), 3.94 (s, 3H), 3.80 (d, J = 11.04 Hz, 1H), 3.59 - 3.72 (m, 1H), 2.15 - 2.25 (m, 1H), 2.10 (s, 3H), 1.98 (dd, J = 6.27, 12.80 Hz, 1H), 1.85 (s, 1H), 1.75 (s, 1H).
ESC1002161 LCMS long method, rt = 2.65 min, m/z [M+H]+ 379.2.
ESC1002162 LCMS long method, rt = 2.65 min, m/z [M+H]+ 379.2.

<u>ESC1002083</u>
Procedure 9 with (2-fluorophenyl)-[7-(methylamino)-3,4-dihydro-2H-quinolin-1-yl]methanone (130 mg, 0.46 mmol), 1H-imidazole-5-carbaldehyde (52.72 mg, 0.55 mmol) and acetic acid (0.04 ml, 0.69 mmol) and sodium triacetoxyborohydride (193.8 mg, 0.91 mmol). Crude product, 190 mg. Product was isolated as 71 mg (42.6%) of (2-fluorophenyl)-[7-[1H-imidazol-5-ylmethyl(methyl)amino]-3,4-dihydro-2H-quinolin-1-yl]methanone. NMR at 298K very broad so high T NMR obtained: 1H NMR (373K, 400 MHz, DMSO-d6) d 7.46 (s, 1H), 7.35 - 7.43 (m,

2H), 7.16 - 7.23 (m, 1H), 7.07 (t, J = 9.29 Hz, 1H), 6.93 (d, J = 8.28 Hz, 1H), 6.63 (s, 1H), 6.53 (dd, J = 2.51, 8.53 Hz, 1H), 6.30 (br. s., 1H), 4.05 (s, 2H), 3.76 (t, J = 6.40 Hz, 2H), 2.68 (t, J = 6.65 Hz, 3H), 2.58 (s, 3H), 1.87 - 2.02 (m, 2H). COSY NMR consistent with product. LCMS long method, rt = 1.61 min, m/z [M+H]+ 365.2.

Procedure 11 (ESC 1002089, ESC1002090, ESC1002091 and ESC1002092)
Suzuki cross coupling: aryl bromide, the desired boronic acid, the solvents (dioxane+water) and the mixture was added to a microwave vial and bubbled with argon for ~5 min. Then the catkit first choice was added to the reaction mixture and the vessel was sealed before heating it to 80°C for the required time. Once completed the mixture was diluted with EtOAc and Water and extracted with EtOAc (3x 15 mL). The org. layers were dried over Na_2SO_4, and reduced before charging the mixture on a 10 g silica cartridge. The mixture was purified on an Isolera and the desired fractions were collected. The solids obtained were further dried in a vac. oven (50°C overnight).
ESC1002331 and ESC1002332 were obtained by SFC chiral separation of ESC1002089.
ESC1002089, 1H NMR (373K, 400 MHz, CDCl3): 7.4 (1H, Ar-H, d, J= 5.1Hz), 7.36-7.30 (1H, Ar-H, m), 7
28-7.20 (2H, Ar-H, m), 7.13-7.03 (3H, Ar-H, m), 7.00-6.95 (2H, Ar-H, m), 6.83 (1H, Ar-H, d, J= 5.0Hz), 5.13-5.06 (1H, CH, m), 3.83 (3H, O-CH3, s), 3.81-3.74 (1H, CH2, m), 3.69-3.60 (1H, CH2, m), 2.94 (3H, CH3, s), 2.19-2.09 (1H, CH2, m), 2.00-1.90 (1H, CH2, m), 1.86-1.75 (1H, CH2, m), 1.75-1.66 (1H, CH2, m) LCMS long method, rt = 2.85 min, m/z [M+H]+ 378.2.

ESC1002090, 1H NMR (373K, 400 MHz, CDCl3): 8.58 (1H, Ar-H, dd, J= 4.9, 1.7Hz), 8.45-4.80 (1H, Ar-H, br), 7.64-7.58 (1H, Ar- H, m), 7.46-7.39 (3H, Ar-H, m), 7.37-7.33 (1H, Ar-H, m), 7.31 (1H, Ar-H, td, J= 7.4, 1.4Hz), 7.13 (1H, Ar-H, dd, J= 7.5, 1.7Hz), 6.83 (1H, A r-H, d, J= 5.0Hz), 4.98 (1H, CH, dd, J= 7.9, 5.7Hz), 3.84-3.76 (1H, CH2, m), 3.69-3.61 (1H, CH2, m), 2.94 (3H, CH3, s), 2.23-2.13 (1H, CH2, m), 2.04-1.93 (1H, CH2, m), 1.89-1.69 (2H, CH2, m) LCMS long method, rt = 1.91 min, m/z [M+H]+ 349.2.

ESC1002091, 1H NMR (373K, 400 MHz, CDCl3): 12.6 (1H, NH, br), 7.74-7.59 (1H, Ar-H, m), 7.46-7.10 (5H, Ar-H, m), 6.82-6.75 (1H, Ar-H, m), 6.41-6.30 (1H Ar-H, m), 5.85-5.60 (1H, CH, m), 3.84-3.76 (1H, CH2, m), 3.74-3.64 (1H, CH2, m), 2.94 (3H, CH3, s), 2.40-2.96 (1H, CH2, m), 1.98-1.72 (3H, CH2, m) LCMS long method, rt = 2.25 min, m/z [M+H]+ 338.2.

ESC1002092, 1H NMR (373K, 400 MHz, CDCl3): 12.66 (1H, NH, br), 7.60-7.52 (2H, Ar-H, m), 7.37 (1H, Ar-H, d, J= 5.2Hz), 7.30-7.17 (4H, Ar-H, m), 6.8 (1H, Ar-H, d, J= 5.0Hz), 5.33-5.27 (1H, CH, m), 3.86-3.77 (1H, CH2, m), 3.72-3.62 (1H, CH2, m), 2.37-2.24 (1H, CH2, m),

2.04-1.83 (2H, CH2, m), 1.79-1.69 (1H, CH2, m). LCMS long method, rt = 2.16 min, m/z [M+H]+ 338.2.

ESC1002331, 1H NMR (373K, 400 MHz, CDCl3): 7.4 (1H, Ar-H, d, J= 5.1Hz), 7.36-7.30 (1H, Ar-H, m), 7 28-7.20 (2H, Ar-H, m), 7.13-7.03 (3H, Ar-H, m), 7.00-6.95 (2H, Ar-H, m), 6.83 (1H, Ar-H, d, J= 5.0Hz), 5.13-5.06 (1H, CH, m), 3.83 (3H, O-CH3, s), 3.81-3.74 (1H, CH2, m), 3.69-3.60 (1H, CH2, m), 2.94 (3H, CH3, s), 2.19-2.09 (1H, CH2, m), 2.00-1.90 (1H, CH2, m), 1.86-1.75 (1H, CH2, m), 1.75-1.66 (1H, CH2, m) LCMS long method, rt = 2.85 min, m/z [M+H]+ 378.2.

ESC1002332, 1H NMR (373K, 400 MHz, DMSO-d6): 7.57-7.49 (1H, Ar-H, m); 7.48-7.24 (7H, Ar-H, m); 7.11-7.02 (1H, Ar-H, m); 6.9 (0.7H, Ar-H, d, J=5.1 Hz); 6.84 (0.3H, Ar-H, d, J=4.9 Hz); 4.97-4.91 (0.3H, CH, m); 4.91-4.85 (0.7H, CH, m); 3.81-3.72 (1H, CH2, m); 3.67-3.56 (1H, CH2, m); 2.18 (0.9H, CH3, s); 2.14 (2.1H, CH3, s); 2.08-1.88 (2H, CH2, m); 1.84-1.66 (2H, CH2, m). LCMS long method, rt = 2.96 min, m/z [M+H]+ 382.2.

ESC1002119

Procedure 10 with [2-(2-bromophenyl)pyrrolidin-1-yl]-(2-fluorophenyl)methanone (85.0 mg, 0.244 mmol), (6-methoxy-3-pyridyl)boronic acid (44.8mg, 0.293mmol) and first choice catkit (127 mg, 0.252 mmol). The desired compound was obtained as a yellow white solid in 49.1 mg (53.5%) of (2-fluorophenyl)-[2-[2-(6-methoxy-3-pyridyl)phenyl]pyrrolidin-1-yl]methanone. 1H NMR (298K, 400 MHz, CDCl3): 8.22 (0.5H, Ar-H, d, J= 2.4Hz); 7.82 (1H, Ar-H, dd, J= 8.5, 2.5Hz); 7.54-7.16 (7H, Ar-H, m); 7.01-6.83 (2H, Ar-H, m); 6.81-6.73 (1H, Ar-H, m), 5.10-5.05 (0.6H, CH, m); 4.66-4.60 (0.4H, CH, m); 3.92 (1.6H, CH3, s); 3.87 (1.4H, CH3, s); 3.77-3.59 (1H, CH2, m); 3.39-3.31 (1H, CH2, m); 2.34-1.60 (5H, CH2, m)

LCMS long method, rt = 2.59 min, m/z [M+H]+ 377.2.

ESC1002120

Procedure 10 with [2-(2-bromophenyl)pyrrolidin-1-yl]-(p-tolyl)methanone (65.0 mg, 0.189 mmol), (6-methoxy-3-pyridyl)boronic acid (34.7 mg, 0.227 mmol) and first choice catkit (98.0 mg, 0.194 mmol). The desired compound was obtained as a yellow-white solid in 40.8 mg (58.0%) of [2-[2-(6-methoxy-3-pyridyl)phenyl]pyrrolidin-1-yl]-(p-tolyl)methanone. 1H NMR (400 MHz, CDCl3): 8.24 (0.7H, Ar-H, d, J= 2.5Hz); 7.87 (0.7H, Ar-H, dd, J= 8.5, 2.4Hz); 7.60 (0.3H, Ar-H, br); 7.55-7.45 (2.2H, Ar-H, m); 7.39 (1H, Ar-H, dd, J= 7.8, 7.4Hz); 7.32-7.21 (2.7H, Ar-H, m); 1.18-7.13 (0.8H, Ar-H, m); 7.00-6.90 (1.7H, Ar-H, m); 6.79-6.72 (0.8H, Ar-H, m), 50.8-5.00 (0.8H, CH, m), 4.75-4.69 (0.3H, CH, m); 3.96-3.78 (4.1H, CH3 + CH2 m); 3.51-3.42

(0.8H, CH2, m); 2.34 (2.2H, CH3, s); 2.29 (0.8H, CH3, s); 2.20-2.09 (1.1H, CH2, m); 1.94-1.83 (1.2H, CH2, m), 1.77-1.57 (0.8H, CH2, m).
2LCMS long method, rt = 2.71 min, m/z [M+H]+ 373.2.

Procedure 12 (ESC1002163 and ESC1002164)

tert-butyl 5-[[3-[(2-fluorobenzoyl)amino]-N-methyl-anilino]methyl]imidazole-1-carboxylate (44 mg, 0.1 mmol) was dissolved in DMF (1 mL) and NaH (60%, 4.98 mg, 0.12 mmol) was added. The reaction was stirred at room temperature for 20 minutes. 1-bromo-2-methoxy-ethane (0.01 mL, 0.1 mmol) was added and stirring continued at room temperature for 4 hours then the reaction was heated to 60°C for 16 hours (LCMS). Additional NaH (60%, 4.98 mg, 0.12 mmol) and 1-bromo-2-methoxy-ethane (0.01 mL, 0.1 mmol) were added and heating to 60°C was continued for 5 hours. The reaction was allowed to cool to room temperature. The reaction mixture was diluted with EtOAc (20 mL) and washed with water (3 x 20 mL) and brine (10 mL). Organics were dried over sodium sulphate, filtered and solvent was evaporated under reduced pressure to afford crude product as a pale-yellow gum, 59mg. Purification by prep. HPLC (basic, 'late' focussed gradient) followed by evaporation of solvent under reduced pressure and drying afforded products EXP-16-FB5962A and EXP-16-FB5962B. EXP-16-FB5962A product of alkylation on imidazole. Regiochemistry of addition confirmed by NMR analysis. Some residual DCM still present so the product was dried under vacuum to afford final product EXP-16-FB5962C, confirmed as ESC1002163. EXP-16-FB5962B product of double alkylation: 4 mg (8.8%) of 2-fluoro-N-(2-methoxyethyl)-N-[3-[[1-(2-methoxyethyl)imidazol-4-yl]methyl-methyl-amino]phenyl]benzamide. Product EXP-16-FB5962B confirmed as ESC1002164-01. EXP-16-FB5962B: 1H NMR (400 MHz, DMSO-d6) d 8.09 (br. s., 1H), 7.23 (d, J = 5.27 Hz, 2H), 6.92 - 7.10 (m, 4H), 6.51 - 6.64 (m, 2H), 6.40 (d, J = 7.28 Hz, 1H), 4.31 (s, 2H), 4.14 (t, J = 5.02 Hz, 2H), 3.94 (t, J = 5.40 Hz, 2H), 3.58 (t, J = 5.02 Hz, 2H), 3.48 (t, J = 5.52 Hz, 2H), 3.15 - 3.30 (m, 6H), 2.84 (s, 3H). LCMS long method, rt = 1.62 min, m/z [M+H]+ 441.2.
EXP-16-FB5962BC 1H NMR (400 MHz, DMSO-d6) d 10.18 (s, 1H), 7.86 (s, 1H), 7.62 (s, 2H), 7.28 - 7.37 (m, 2H), 7.19 (s, 1H), 6.98 - 7.13 (m, 3H), 6.56 (dd, J = 1.51, 8.03 Hz, 1H), 4.39 (s, 2H), 4.09 (t, J = 5.14 Hz, 2H), 3.56 (t, J = 5.14 Hz, 2H), 3.20 (s, 3H), 2.95 (s, 3H). Proton at 10ppm characteristic of amide.
NOESY NMR EXP-16-FB5962A_NOESY confirms regiochemistry of alkylation of imidazole.
LCMS long method, rt = 1.59 min, m/z [M+H]+ 383.2.

Procedure 13 (ESC1002165)

tert-butyl 5-[[3-(isobutylamino)-N-methyl-anilino]methyl]imidazole-1-carboxylate (45 mg, 0.13 mmol) was dissolved in DCM (1 mL) and DIEA (0.11 ml, 0.63 mmol) was added. The solution

was cooled in an ice-bath and 2-methylpropanoyl chloride (0.02 ml, 0.19 mmol) was added. The reaction was allowed to warm to room temperature and stirred for 3 hours (LCMS). The reaction mixture was diluted with DCM (5 mL) and washed with water (2 mL). Organics were filtered through a hydrophobic frit and solvent was evaporated to afford crude intermediate. The intermediate was dissolved in HCl (4M diox., 1 mL) and stirred at room temperature for 3 hours. Solvent was evaporated under reduced pressure. The afforded residue was dissolved in 1:1 DMSO / MeOH and sodium carbonate added. The suspension was stirred for 30 minutes and filtered. Purification by prep. HPLC (basic) followed by evaporation of solvent from the appropriate fractions. The resulting residues were dissolved in DCM and combined in a vial. Solvent was evaporated and the resulting gum dried under vacuum. The afforded gum was difficult to handle so was dissolved in MeOH / water then solvent was evaporated (genevac, HPLC lyophilisation program). The afforded pale yellow gum was dried under vacuum to afford product, 23 mg (55.8%) of N-[3-[1H-imidazol-5-ylmethyl(methyl)amino]phenyl]-N-isobutyl-2-methyl-propanamide. 1H NMR (400 MHz, DMSO-d6) d 11.69 - 12.14 (m, 1H), 7.54 (d, J = 1.00 Hz, 1H), 7.19 (t, J = 8.03 Hz, 1H), 6.84 (s, 1H), 6.75 (dd, J = 2.01, 8.28 Hz, 1H), 6.60 (s, 1H), 6.45 (d, J = 7.53 Hz, 1H), 4.40 (s, 2H), 3.40 (d, J = 7.28 Hz, 2H), 2.99 (s, 3H), 2.41 (m, 1H), 1.56 - 1.69 (m, 1H), 0.87 (d, J = 6.53 Hz, 6H), 0.80 (d, J = 6.53 Hz, 6H).
LCMS long method, rt = 1.67 min, m/z [M+H]+ 329.2.

ESC1002166

Procedure 13 with cyclohexanecarbonyl chloride (0.03 ml, 0.19 mmol) in place of 2-methylpropanoyl chloride. The product was obtained as 27 mg (58.4%) of N-[3-[1H-imidazol-5-ylmethyl(methyl)amino]phenyl]-N-isobutyl-cyclohexanecarboxamide. 1H NMR (400 MHz, DMSO-d6) d 11.87 (br. s., 1H), 7.52 (d, J = 0.75 Hz, 1H), 7.19 (t, J = 8.03 Hz, 1H), 6.70 - 6.87 (m, 2H), 6.59 (s, 1H), 6.44 (d, J = 7.53 Hz, 1H), 4.40 (s, 2H), 3.39 (d, J = 7.28 Hz, 2H), 2.99 (s, 3H), 2.13 (m, 1H), 1.43 - 1.70 (m, 6H), 1.32 (m, 2H), 1.00 - 1.14 (m, 1H), 0.82 - 0.93 (m, 2H), 0.79 (d, J = 6.53 Hz, 6H)
LCMS long method, rt = 1.80 min, m/z [M+H]+ 369.2.

ESC1002167

Procedure 13 with 2-phenylacetyl chloride (0.02 ml, 0.19 mmol) in place of 2-methylpropanoyl chloride. The product was obtained as a pale yellow gum, 25 mg (52.9%) of N-[3-[1H-imidazol-5-ylmethyl(methyl)amino]phenyl]-N-isobutyl-2-phenyl-acetamide. 1H NMR (400 MHz, DMSO-d6) d 11.90 (br. s., 1H), 7.55 (d, J = 0.75 Hz, 1H), 7.13 - 7.28 (m, 4H), 7.01 (d, J = 7.03 Hz, 2H), 6.84 (s, 1H), 6.77 (dd, J = 1.88, 8.41 Hz, 1H), 6.58 (t, J = 1.88 Hz, 1H), 6.46 (d, J = 7.53 Hz, 1H), 4.37 (s, 2H), 3.45 (d, J = 7.28 Hz, 2H), 3.36 (s, 2H), 2.93 (s, 3H), 1.62 (td, J = 6.78, 13.55 Hz, 1H), 0.81 (d, J = 6.53 Hz, 6H).

LCMS long method, rt = 1.74 min, m/z [M+H]+ 377.2.

ESC1002168

Procedure 13 with 3-phenylpropanoyl chloride (0.03 ml, 0.19 mmol) in place of 2-methylpropanoyl chloride. The product was obtained as a pale yellow gum, 39 mg (79.6%) of N-[3-[1H-imidazol-5-ylmethyl(methyl)amino]phenyl]-N-isobutyl-3-phenyl-propanamide. 1H NMR (400 MHz, DMSO-d6) d 11.91 (br. s., 1H), 7.53 (d, J = 0.75 Hz, 1H), 7.20 (d, J = 7.53 Hz, 2H), 7.09 - 7.17 (m, 2H), 7.04 (d, J = 7.28 Hz, 2H), 6.84 (s, 1H), 6.71 (dd, J = 2.01, 8.28 Hz, 1H), 6.57 (s, 1H), 6.36 (d, J = 7.53 Hz, 1H), 4.37 (s, 2H), 3.43 (d, J = 7.28 Hz, 2H), 2.94 (s, 3H), 2.74 (t, J = 7.65 Hz, 2H), 2.28 (t, J = 7.65 Hz, 2H), 1.49 - 1.66 (m, 1H), 0.79 (d, J = 6.78 Hz, 6H).

LCMS long method, rt = 1.82 min, m/z [M+H]+391.2.

ESC1002169

Procedure 13 with benzoyl chloride (0.02 ml, 0.19 mmol) in place of 2-methylpropanoyl chloride. The product was obtained as a yellow gum, 26 mg (57.1%) of N-[3-[1H-imidazol-5-ylmethyl(methyl)amino]phenyl]-N-isobutyl-benzamide. 1H NMR (400 MHz, DMSO-d6) d 11.83 (br. s., 1H), 7.53 (s, 1H), 7.09 - 7.29 (m, 5H), 6.95 (br. s., 1H), 6.68 (br. s., 1H), 6.54 (br. s., 2H), 6.22 - 6.39 (m, 1H), 4.26 (br. s., 2H), 3.67 (d, J = 7.28 Hz, 2H), 2.84 (s, 3H), 1.65 - 1.84 (m, 1H), 0.88 (d, J = 6.78 Hz, 6H). COSY NMR consistent with product.

LCMS long method, rt = 1.68 min, m/z [M+H]+ 363.2.

Procedure 14 (ESC1002170)

1-(2-methylbenzofuran-3-yl)-2-phenyl-ethanone (110 mg, 0.44 mmol) was suspended in ethanol (2 mL) and $NH_2NH_2 \cdot H_2O$ (0.04 mL, 0.88 mmol) was added. The reaction was sealed in a vial and heated to 60°C for 2 hours (LCMS) then allowed to stand at room temperature for 16 hours. Solvent was evaporated under reduced pressure and the resulting residue partitioned between EtOAc (15 mL) and water (10 mL). Organics were washed with brine, dried over sodium sulphate, filtered and solvent was evaporated under reduced pressure to afford crude product, 140 mg. Purification by flash chromatography (silica column, heptane 10% to 100% EtOAc gradient) followed by evaporation of solvent from the appropriate fractions and drying under vacuum afforded product 5978A as a sticky white solid, 34 mg (note - yield sacrificed for purity). NMR analysis (^1H, HSQC, NOE) confirmed desired product formation. Weighing the solid was problematic so the solid was dissolved in MeOH / water, transferred to a vial and the solution concentrated under reduced pressure (genevac, HPLC lyophilisation program) and dried under vacuum to afford product, 21 mg (18.1%) of 2-(5-benzyl-3-methyl-1H-pyrazol-4-yl)phenol. 1H NMR (400 MHz, DMSO-d6) d 12.25 (s, 1H), 9.21

(s, 1H), 7.05 - 7.23 (m, 4H), 7.02 (d, J = 7.03 Hz, 2H), 6.85 - 6.97 (m, 2H), 6.76 (t, J = 7.28 Hz, 1H), 3.80 (br. s., 2H), 2.05 (br. s., 3H). HSCQ / NOESY (EXP-16-FB5978A) both consistent with desired product.
LCMS long method, rt = 2.16 min, m/z [M+H]+ 265.2.

ESC1002171
Procedure 13 with acetyl chloride (0.01 ml, 0.18 mmol) in place of 2-methylpropanoyl chloride. The product was isolated as a yellow gum, 22 mg (62.5%) of N-[3-[1H-imidazol-5-ylmethyl(methyl)amino]phenyl]-N-isobutyl-acetamide. 1H NMR @ 373K (400 MHz, DMSO-d6) d 11.17 - 12.05 (br. s, 1H), 7.48 (s, 1H), 7.18 (t, J = 8.03 Hz, 1H), 6.81 (br. s., 1H), 6.76 (dd, J = 2.13, 8.41 Hz, 1H), 6.65 (t, J = 2.13 Hz, 1H), 6.47 (d, J = 7.28 Hz, 1H), 4.40 (s, 2H), 3.45 (d, J = 7.28 Hz, 2H), 2.92 (s, 3H), 1.76 (s, 3H), 1.71 (m, 1H), 0.84 (d, J = 6.78 Hz, 6H).
LCMS long method, rt = 1.50 min, m/z [M+H]+ 301.2.

ESC1002172
Procedure 13 with naphthalene-1-carbonyl chloride (0.03 ml, 0.19 mmol) in place of 2-methylpropanoyl chloride. The product was isolated as a pale yellow solid, 22 mg (42.5%) of N-[3-[1H-imidazol-5-ylmethyl(methyl)amino]phenyl]-N-isobutyl-naphthalene-1-carboxamide.
1H NMR @298K (400 MHz, DMSO-d6) d 11.63 - 12.01 (m, 1H), 7.98 (d, J = 8.28 Hz, 1H), 7.86 (d, J = 8.03 Hz, 1H), 7.75 (d, J = 8.03 Hz, 1H), 7.54 - 7.61 (m, 1H), 7.52 (s, 2H), 7.23 (d, J = 7.53 Hz, 1H), 7.15 (d, J = 6.78 Hz, 1H), 6.79 (t, J = 8.03 Hz, 1H), 6.53 (br. s., 1H), 6.45 (br. s., 1H), 6.34 - 6.41 (m, 1H), 6.29 (br. s., 1H), 4.07 (br. s., 2H), 3.83 (br. s., 2H), 2.61 (s, 3H), 1.68 - 1.85 (m, 1H), 0.98 (d, J = 6.53 Hz, 6H). COSY NMR consistent with product.
1H NMR @373K (400 MHz, DMSO-d6) d 11.52 (br. s., 1H), 8.00 (d, J = 8.28 Hz, 1H), 7.83 (d, J = 8.03 Hz, 1H), 7.74 (d, J = 8.03 Hz, 1H), 7.51 - 7.59 (m, 1H), 7.43 - 7.51 (m, 2H), 7.23 - 7.31 (m, 1H), 7.17 - 7.23 (m, 1H), 6.84 (br. s., 1H), 6.56 (br. s., 1H), 6.50 (br. s., 1H), 6.44 (d, J = 8.28 Hz, 1H), 6.36 (br. s., 1H), 4.10 (br. s., 2H), 3.79 (d, J = 7.03 Hz, 2H), 2.64 (br. s., 3H), 1.77 - 1.97 (m, 1H), 0.97 (d, J = 6.78 Hz, 6H)
LCMS long method, rt = 1.81 min, m/z [M+H]+ 413.2.

ESC1002182
Procedure 13 with 4-methoxybenzoyl chloride (32.12 mg, 0.19 mmol) in place of 2-methylpropanoyl chloride. ^{1}H NMR / LCMS indicated product was not pure. Purification by prep. HPLC (basic, 'late' focussed gradient method) followed by evaporation of solvent from the appropriate fractions afforded the product as a sticky gum. The gum was dissolved in EtOH and water and solvent evaporated (genevac, lyophilisation program) to afford a solid. Drying the solid under vacuum caused the solid to melt and form a gum - product, 14 mg

(28.4%) of N-[3-[1H-imidazol-5-ylmethyl(methyl)amino]phenyl]-N-isobutyl-4-methoxy-benzamide. 1H NMR (400 MHz, DMSO-d6) d 11.74 - 12.00 (m, 1H), 7.44 - 7.62 (m, 1H), 7.18 (d, J = 8.78 Hz, 2H), 6.95 (t, J = 8.03 Hz, 1H), 6.77 (s, 1H), 6.71 (d, J = 8.78 Hz, 2H), 6.50 - 6.60 (m, 2H), 6.23 (d, J = 7.53 Hz, 1H), 4.29 (s, 2H), 3.69 (s, 3H), 3.65 (d, J = 7.28 Hz, 2H), 2.88 (s, 3H), 1.74 (s, 1H), 0.86 (d, J = 6.78 Hz, 6H).
Rotamers observed in NMR but assigned as correct structure (and not impurities) without high T NMR based on LCMS and precedent with closely related compounds
LCMS long method, rt = 1.74 min, m/z [M+H]+ 393.2.

ESC1002183
Procedure 13 with 4-chlorobenzoyl chloride (0.02 ml, 0.19 mmol) in place of 2-methylpropanoyl chloride. The product was isolated as 9 mg (18.1%) of 4-chloro-N-[3-[1H-imidazol-5-ylmethyl(methyl)amino]phenyl]-N-isobutyl-benzamide. 1H NMR @298K (400 MHz, DMSO-d6) d 11.78 - 11.96 (br. s, 1H), 7.55 (s, 1H), 7.18 - 7.31 (m, 4H), 6.96 (t, J = 8.03 Hz, 1H), 6.75 (br. s., 1H), 6.60 (s, 1H), 6.52 - 6.57 (m, 1H), 6.26 (d, J = 7.53 Hz, 1H), 4.31 (s, 2H), 3.66 (d, J = 7.53 Hz, 2H), 2.87 (s, 3H), 1.66 - 1.81 (m, 1H), 0.87 (d, J = 6.53 Hz, 6H)
LCMS long method, rt = 1.86 min, m/z [M+H]+ 397.2.

Procedure 15 (ESC1002199 to ESC1002214)
2-fluoro-N-isobutyl-N-[3-(methylamino)phenyl]benzamide (198.25 mg, 0.66 mmol) was added to a vial containing the desired aldehyde (24.9 mg, 0.86 mmol) and dissolved in CH_3CO_2H (1 mL, 17.48 mmol). To the mixture was added $(CH_3COO)_3BHNa$ (209.82 mg, 0.99 mmol). The mixture was stirred overnight at rt. The solutions were quenched with 2M NaOH (2ml) and extracted with EtOAc (3 x 10 ml). The organic layers were dried over Na_2SO_4 then reduced. Some compounds were purified on an Isolera (Biotage) whereas other were purified on a basic prep. Most compounds were gums, therefore it was decided to salt them by addition of 2 mL Et_2O and 1.2eq of 4M HCl in Dioxane. Most compounds seems to have salted successfully, although some showed sign of degradation. Therefore, they were re-purified by prep HPLC (basic) and submitted as free base.
ESC1002199, 1H NMR (400 MHz, DMSO-d6): 14.3 (1H, NH+, br); 9.00 (1H, Ar-H, s); 7.29-7.18 (2H, Ar-H, m); 7.08-6.95 (4H, Ar-H, m); 6.62 (1H, Ar-H, d, J= 8.9Hz); 6.57 (1H, Ar-H, s); 6.49 (1H, Ar-H, d, J= 8.0Hz); 4.57 (2H, CH2, s); 3.73 (3H, CH3, s); 3.68 (2H, CH2, d, J= 7.3Hz); 2.83 (3H, CH3, s); 1.81-1.68 (1H, CH, m); 0.91 (6H, CH3, d, J= 6.7Hz); LCMS long method, rt = 1.81 min, m/z [M+H]+ 395.2.

ESC1002200, 1H NMR (400 MHz, DMSO-d6): 14.6 (1H, NH+, br); 8.96 (1H, Ar-H, s); 7.28 (1H, Ar-H, s); 7.24 (1H, Ar-H, d, J= 6.8Hz); 7.22 (1H, Ar-H, d, J= 6.1Hz); 7.08-6.93 (3H, Ar-H,

m); 6.60 (1H, Ar-H, d, J= 8.6Hz); 6.51 (1H, Ar-H, s); 6.47 (1H, Ar-H, d, J= 7.9Hz); 4.47 (2H, CH2, s); 3.79 (3H, CH3, s); 3.68 (2H, CH2, d, J= 7.1Hz); 2.87 (3H, CH3, s); 1.78-1.65 (1H, CH, m); 0.90 (6H, CH3, d, J= 6.6Hz); LCMS long method, rt = 1.74 min, m/z [M+H]+ 395.2.

ESC1002201, 1H NMR (400 MHz, DMSO-d6): 13.9 (2H, NH + NH+, br); 7.50 (2H, Ar-H, s); 7.28-7.13 (2H, Ar-H, m); 7.07-6.94 (3H, Ar-H, m); 6.63-6.55 (1H, Ar-H, m); 6.50-6.43 (2H, Ar-H, m); 4.67 (2H, CH2, s); 3.64 (2H, CH2, d, J= 7.5Hz); 2.96 (3H, CH3, s); 1.73-1.62 (1H, CH, m); 0.88 (6H, CH3, d, J= 6.7Hz); LCMS long method, rt = 1.67 min, m/z [M+H]+ 381.2.

ESC1002202, 1H NMR (400 MHz, DMSO-d6): 14.2 (1H, NH+, br); 7.69 (1H, Ar-H, d, J= 1.9Hz); 7.57 (1H, Ar-H, d, J= 2.0Hz); 7.30-7.17 (2H, Ar-H, m); 7.09-6.95 (3H, Ar-H, m); 6.61 (1H, Ar-H, d, J= 8.7Hz); 6.55 (1H, Ar-H, s); 6.51 (1H, Ar-H, d, J= 8.3Hz); 4.79 (2H, CH2, s); 3.72 (3H, CH3, s); 3.66 (1H,CH2, d, J= 8.0Hz); 2.90 (3H, CH3, s); 1.78-1.67 (1H, CH, m); 0.90 (6H, CH3, d, J= 6.6 Hz) LCMS long method, rt = 1.71 min, m/z [M+H]+ 395.2.

ESC1002203, 1H NMR (400 MHz, DMSO-d6): 13.1 (2H, NH + NH+, br); 7.29-7.15 (2H, Ar-H, s); 7.05-6.94 (2H, Ar-H, m); 6.81 (1H, Ar-H, s); 6.59-6.50 (2H, Ar-H, m); 6.47 (2H, Ar-H, d, J= 7.6Hz); 4.30 (2H, CH2, s); 3.67 (2H, CH2, d, J= 7.3Hz); 2.83 (3H, CH3, s); 2.37 (3H, CH3, s); 1.77-1.66 (1H, CH, m); 0.90 (6H, CH3, d, J= 6.6Hz); LCMS long method, rt = 1.74 min, m/z [M+H]+ 395.2.

ESC1002204, 1H NMR (400 MHz, DMSO-d6): 11.9 (1H, NH + NH+, br); 7.52-7346 (1H, Ar-H, m); 7.26-7.12 (2H, Ar-H, m); 7.04-6.92 (3H, Ar-H, m); 6.57-6.51 (2H, Ar-H, m); 6.39-6.33 (1H, Ar-H, m); 4.20 (2H, CH2, s); 3.66 (2H, CH2, d, J= 7.9Hz); 2.78 (3H, CH3, s); 2.02 (3H, CH3, s); 1.77-1.66 (1H, CH, m); 0.90 (6H, CH3, d, J= 6.8Hz); LCMS long method, rt = 1.76 min, m/z [M+H]+ 395.2.

ESC1002205, 1H NMR (400 MHz, DMSO-d6): 12.5 (1H, NH + NH+, br); 7.54 (1H, Ar-H, s); 7.28-7.11 (2H, Ar-H, m); 7.04-6.89 (3H, Ar-H, m); 6.58-6.48 (2H, Ar-H, m); 6.37 (1H, Ar-H, d, J= 7.7Hz); 5.87-5.82 (1H, Ar-H, m); 4.37(2H, CH2, s); 3.65 (2H, CH2, d, J= 7.6Hz); 2.83 (3H, CH3, s); 1.74 -1.67 (1H, CH, m); 0.90 (6H, CH3, d, J= 6.6Hz); LCMS long method, rt = 1.74 min, m/z [M+H]+ 395.2.

ESC1002206, 1H NMR (400 MHz, DMSO-d6): 7.36-7.14 (4H, Ar-H, m); 7.05-6.91 (3H, Ar-H, m); 6.55-6.48 (2H, Ar-H, m); 6.38 (1H, Ar-H, d, J= 8.0Hz); 4.28 (2H, CH2, s); 3.67 (2H, CH2, d, J= 7.4Hz); 2.76 (3H, CH3, s); 1.76 -1.67 (1H, CH, m); 0.90 (6H, CH3, d, J= 6.5Hz); LCMS long method, rt = 2.38 min, m/z [M+H]+ 381.2.

ESC1002207, 1H NMR (400 MHz, DMSO-d6): 7.57-7.53 (1H, Ar-H, m); 7.28-7.20 (1H, Ar-H, m); 7.19-7.12 (1H, Ar-H, m); 7.05-6.92 (3H, Ar-H, m); 6.57-6.21 (2H, Ar-H, m); 6.38 (1H, Ar-H, d, J= 7.9Hz); 5.88-5.83 (1H, Ar-H, m); 4.38 (2H, CH2, s); 3.65 (2H, CH2, d, J= 7.4Hz); 2.84 (3H, CH3, s); 1.77-1.65 (1H, CH, m); 0.89 (6H, CH3, d, J= 6.6Hz); LCMS long method, rt = 2.45 min, m/z [M+H]+ 381.2

ESC1002208, 1H NMR (400 MHz, DMSO-d6): 8.94 (1H, Ar-H, d, J= 0.7Hz); 7.70 (1H, Ar-H, s); 7.29-7.14 (2H, Ar-H, m); 7.04-6.94 (3H, Ar-H, m); 6.61-6.54 (2H, Ar-H, m); 6.44 (1H, Ar-H, d, J= 7.8Hz); 4.71 (2H, CH2, s); 3.68 (2H, CH2, d, J= 7.0Hz); 2.78 (3H, CH3, s); 1.77-1.66 (1H, CH, m); 0.90 (6H, CH3, d, J= 6.7Hz); LCMS long method, rt = 2.62 min, m/z [M+H]+ 398.2

ESC1002209, 1H NMR (400 MHz, DMSO-d6): 8.63 (1H, Ar-H, d, J= 5.2Hz); 8.00-7.88 (1H, Ar-H, m); 7.51-7.41 (1H, Ar-H, m); 7.29-7.19 (1H, Ar-H, m); 7.16-7.05 (2H, Ar-H, m); 7.03-6.92 (3H, Ar-H, m); 6.51 (1H, Ar-H, d, J= 8.8Hz); 6.45-6.36 (2H, Ar-H, m); 4.62 (2H, CH2, s); 3.61 (2H, CH2, d, J= 7.4Hz); 2.99 (3H, CH3, s); 1.70-1.59 (1H, CH, m); 0.84 (6H, CH3, d, J= 6.6Hz); LCMS long method, rt = 2.34 min, m/z [M+H]+ 392.2

ESC1002210, 1H NMR (400 MHz, DMSO-d6): 8.73 (1H, Ar-H, d, J= 5.2Hz); 8.55 (1H, Ar-H, s); 7.99-7.92 (1H, Ar-H, m); 7.86-7.78 (1H, Ar-H, m); 7.29-7.21 (1H, Ar-H, m); 7.19-7.11 (1H, Ar-H, m); 7.05-6.94 (3H, Ar-H, m); 6.55 (1H, Ar-H, d, J= 7.7Hz); 6.48-6.41 (2H, Ar-H, s); 4.64 (2H, CH2, s); 3.63 (2H, CH2, d, J= 7.5Hz); 2.95 (3H, CH3, s); 1.72-1.61 (1H, CH, m); 0.86 (6H, CH3, d, J= 6.7Hz); LCMS long method, rt = 2.12 min, m/z [M+H]+ 392.2

ESC1002211, 1H NMR (400 MHz, DMSO-d6): 8.95-8.44 (2H, Ar-H, m); 7.31-7.22 (1H, Ar-H, m); 7.12 (1H, Ar-H, t, J= 7.2Hz); 7.05-6.95 (5H, Ar-H, m); 6.45 (1H, Ar-H, dd, J= 17.5, 8.8Hz); 6.35 (1H, Ar-H, s); 4.50 (2H, CH2, s); 3.62 (2H, CH2, d, J= 7.3Hz); 2.93 (3H, CH3, s); 1.72-1.59 (1H, CH, m); 0.84 (6H, CH3, d, J= 6.6Hz); LCMS long method, rt = 1.98 min, m/z [M+H]+ 392.2

ESC1002212, 1H NMR (400 MHz, DMSO-d6): 11.47 (1H, OH, br); 7.18-6.95 (7H, Ar-H, m); 6.51 (1H, Ar-H, dd, J= 8.5, 2.0Hz); 6.47 (1H, Ar-H, s); 6.42 (1H, Ar-H, d, J= 8.1Hz); 6.27 (1H, Ar-H, d, J= 9.4Hz); 4.16 (2H, CH2, s); 3.66 (2H, CH2, d, J= 7.5Hz); 2.79 (3H, CH3, s); 1.77-1.65 (1H, CH, m); 0.89 (6H, CH3, d, J= 6.7Hz); LCMS long method, rt = 2.30 min, m/z [M+H]+ 506.2

ESC1002213, 1H NMR (400 MHz, DMSO-d6): 8.06 (1H, Ar-H, d, J= 5.3Hz); 7.28-7.19 (1H, Ar-H, m); 7.15-7.09 (1H, Ar-H, m); 7.01-6.93 (3H, Ar-H, m); 6.48 (1H, Ar-H, d, J= 5.3Hz); 6.65-6.49 (3H, Ar-H, m); 6.34 (1H, Ar-H, s); 4.45 (2H, CH2, s); 3.82 (3H, CH3, s); 3.61 (2H, CH2, d, J= 7.4Hz); 2.92 (3H, CH3, s); 1.68-1.60 (1H, CH, m); 0.84 (6H, CH3, d, J= 6.7Hz); LCMS long method, rt = 2.84 min, m/z [M+H]+ 422.2

ESC1002214, 1H NMR (400 MHz, DMSO-d6): 8.45 (1H, Ar-H, dt, J= 6.8, 1.2Hz); 7.51 (1H, Ar-H, s); 7.47 (1H, Ar-H, dq, J= 9.1, 0.7Hz); 7.24-7.08 (3H, Ar-H, m); 7.01-6.81 (4H, Ar-H, m); 6.56 (1H, Ar-H, d, J= 8.9Hz); 6.51 (1H, Ar-H, s); 6.37 (1H, Ar-H, d, J= 7.8Hz); 4.51 (2H, CH2, s); 3.61 (2H, CH2, d, J=7.4Hz); 2.98 (3H, CH3, s); 1.68-1.58 (1H, CH, m); 0.80 (6H, CH3, d, J= 6.6Hz) LCMS long method, rt = 1.82 min, m/z [M+H]+ 431.2.

Procedure 16 (ESC1002234)

tert-butyl 5-[[3-[(2-fluorobenzoyl)amino]-N-methyl-anilino]methyl]imidazole-1-carboxylate (114 mg, 0.27 mmol) was dissolved in DMF (1ml) and cooled to 0°C. NaH (60%, 12.89 mg, 0.32 mmol) was added and the mixture stirred for 15 minutes. Ethyliodide (0.03 ml, 0.34 mmol) was added then the reaction mixture placed under argon, stirred at 0°C for 10 minutes and then allowed to warm to room temperature. DMF (1 mL) was added to aid stirring and the reaction stirred for 80 hours (LCMS t = 5 hours, t = 80 hours. The reaction mixture was partitioned between EtOAc (30ml) and water (20 mL). Organics were separated, washed with water (2 x 20 mL) and brine (10 mL) dried over sodium sulphate, filtered and solvent was evaporated under reduced pressure to afford crude product. The crude product was partially deprotected so the residue was dissolved in HCl (4M in dioxane, 2 mL) and stirred at room temperature for 3 hours to afford complete deprotection. Solvent was evaporated under reduced pressure and the resulting residue dissolved in 1:1 DMSO / MeOH. Sodium carbonate was added and the mixture stirred for 30 minutes and then filtered. Purification of the resulting solution by prep. HPLC (basic, late focussed gradient, multiple very small quantity injections required for peak separation) followed by evaporation of solvent from the appropriate fractions afforded product, 25 mg. Analytical data consistent with desired product (^{1}H NMR @298K, ^{19}F NMR and COSY NMR, LCMS; ^{1}H NMR @ 373K but product contains some EtOAc residue. Product was dissolved in EtOH and water and solvent was evaporated (genevac, lyophilisation program). The afforded solid was dried under vacuum to afford product, 20 mg. ^{1}H NMR indicates no EtOAc but other aliphatic impurities are present. Purification by prep. HPLC (basic, late focussed gradient) followed by evaporation of solvent from the appropriate fractions and drying under vacuum afforded product, 14 mg (14.8%) of N-ethyl-2-fluoro-N-[3-[1H-imidazol-5-ylmethyl(methyl)amino]phenyl]benzamide. 1H NMR (400 MHz, DMSO-d6) d

11.87 (br. s., 1H), 7.54 (d, J = 0.75 Hz, 1H), 7.13 - 7.33 (m, 2H), 6.89 - 7.08 (m, 3H), 6.66 (s, 1H), 6.48 - 6.60 (m, 2H), 6.35 (d, J = 7.53 Hz, 1H), 4.28 (s, 2H), 3.81 (d, J = 6.78 Hz, 2H), 2.83 (s, 3H), 1.10 (s, 3H).
LCMS long method, rt = 1.57 min, m/z [M+H]+ 353.2.

Procedure 17 (ESC1002235)

To a solution of tert-butyl 5-[[3-(isobutylamino)-N-methyl-anilino]methyl]imidazole-1-carboxylate (42 mg, 0.12 mmol) in NMP (1 mL) was added DIEA (0.06 ml, 0.35 mmol). The solution was cooled to 0°C and butanoyl chloride (18.73 mg, 0.18 mmol) added. The reaction was allowed to warm to room temperature and stirred for 90 minutes (LCMS). The reaction mixture was diluted with DCM (5ml) and washed with NaOH (2M aq., 4 mL). Organics were filtered through a hydrophobic frit and the mixture concentrated to afford an NMP solution of crude product. The solution was diluted with HCl (4M diox., 2 mL) and the mixture stirred for 2 hours and concentrated to remove dioxane and excess HCl. Sodium carbonate was added to the resulting NMP solution and the mixture stirred for 30 minutes and then filtered. Purification by prep. HPLC (basic) followed by evaporation of solvent from the appropriate fractions and drying afforded product as a gum, 18mg. ^{1}H NMR indicates product is not sufficiently pure for submission for testing. Purification by prep. HPLC (basic, late focussed gradient) followed by evaporation of solvent from the appropriate fractions and drying under vacuum afforded product, 15 mg (39%) of N-[3-[1H-imidazol-5-ylmethyl(methyl)amino]phenyl]-N-isobutyl-butanamide. 1H NMR (400 MHz, DMSO-d6) d 11.84 (br. s., 1H), 7.53 (s, 1H), 7.17 (m, 1H), 6.87 (s, 1H), 6.73 (d, J = 7.78 Hz, 1H), 6.60 (br. s., 1H), 6.43 (d, J = 7.53 Hz, 1H), 4.38 (s, 2H), 3.43 (d, J = 7.28 Hz, 2H), 2.99 (s, 3H), 1.95 (m, 2H), 1.51 - 1.68 (m, 1H), 1.42 (m, 2H), 0.81 (d, J = 6.53 Hz, 6H), 0.73 (t, J = 7.28 Hz, 3H). Restricted rotation observed at 298K, consistent with previously synthesised compounds in series so no VT NMR performed.
LCMS long method, rt = 1.66 min, m/z [M+H]+ 329.2.

Procedure 18 (ESC1002236)

tert-butyl 5-[[7-(isobutylamino)-3,4-dihydro-2H-quinolin-1-yl]methyl]imidazole-1-carboxylate (50%, 79 mg, 0.1 mmol) was dissolved in DCM (1ml) and DIEA (0.18 ml, 1.03 mmol) was added. The solution was cooled to 0°C and 2-fluorobenzoyl chloride (0.02 ml, 0.15 mmol) was added. The reaction was allowed to warm to room temperature and stirred for 3 hours (LCMS). The reaction mixture was diluted with DCM (5ml) and washed with water (2 mL). Organics were filtered through a hydrophobic frit and solvent was evaporated to afford crude intermediate. Purification by prep. HPLC (basic) followed by evaporation of solvent from the appropriate fractions afforded product. ^{1}H NMR / LCMS indicates product is not sufficiently

pure for submission for testing. Purification by flash chromatography (silica column, DCM 0% to 75% gradient of a solution of DCM:MeOH:NH_4OH 90:10:1) followed by evaporation of solvent from the appropriate fractions and drying under vacuum afforded product, 10 mg (23.9%) of 2-fluoro-N-[1-(1H-imidazol-5-ylmethyl)-3,4-dihydro-2H-quinolin-7-yl]-N-isobutyl-benzamide. 1H NMR (400 MHz, DMSO-d6) d 11.90 (br. s., 1H), 7.57 (s, 1H), 7.24 (d, J = 7.28 Hz, 1H), 7.11 (s, 1H), 6.96 - 7.07 (m, 2H), 6.76 (s, 1H), 6.64 (d, J = 7.78 Hz, 1H), 6.49 (s, 1H), 6.17 (d, J = 7.53 Hz, 1H), 4.20 (s, 2H), 3.59 (d, J = 7.28 Hz, 2H), 1.71 - 1.85 (m, 2H), 1.64 (br. s., 1H), 0.86 (d, J = 6.78 Hz, 6H).
LCMS long method, rt = 1.83 min, m/z [M+H]+ 407.2.

Procedure 19 (ESC1002266)
2-fluoro-N-(2-methoxyethyl)-N-[3-(methylamino)phenyl]benzamide (200 mg, 0.66 mmol) and 1H-imidazole-5-carbaldehyde (76.27 mg, 0.79 mmol) were suspended in DCM (2 mL) and acetic acid (0.06 ml, 0.99 mmol) was added. The mixture was stirred for 20 minutes then sodium triacetoxyborohydride (280.39 mg, 1.32 mmol) was added and stirring continued at room temperature for 18 hours (LCMS). The reaction mixture was diluted with DCM (30 mL) and washed with NaOH (1M aq., 10 mL). The aqueous wash was extracted with DCM (10 mL) then organics were combined, washed with brine, dried over sodium sulphate, filtered and solvent was evaporated under reduced pressure to afford crude product, 362 mg. Purification by flash chromatography (silica column, DCM 0% to 100% gradient of a solution of DCM:MeOH:NH4OH 100:10:1) followed by evaporation of solvent from the appropriate fractions afforded product 0500A as a colourless gum. Not sufficiently pure for submission for testing. Purification by prep. HPLC (basic) followed by evaporation of solvent from the appropriate fractions afforded product 0500B. Analysis confirms product is pure for testing but contains residual MeOH. Further drying under vacuum afforded product as a colourless gum, 180 mg (71.2%) of 2-fluoro-N-[3-[1H-imidazol-4-ylmethyl(methyl)amino]phenyl]-N-(2-methoxyethyl)benzamide. 1H NMR (400 MHz, DMSO-d6) d 11.88 (br. s., 1H), 7.55 (d, J = 0.75 Hz, 1H), 7.14 - 7.32 (m, 2H), 6.91 - 7.07 (m, 3H), 6.66 (s, 1H), 6.51 - 6.60 (m, 2H), 6.38 (d, J = 7.53 Hz, 1H), 4.28 (s, 2H), 3.93 (t, J = 5.77 Hz, 2H), 3.47 (t, J = 5.77 Hz, 2H), 3.23 (s, 3H), 2.82 (s, 3H). 19F / COSY NMR (analysis of EXP-16-FJ0500B prior to drying) consistent with desired product. LCMS long method, rt = 1.55 min, m/z [M+H]+ 383.2.

ESC1002267
Procedure 19 with 2-fluoro-N-(2-isopropoxyethyl)-N-[3-(methylamino)phenyl]benzamide (151 mg, 0.46 mmol), 1H-imidazole-5-carbaldehyde (52.7 mg, 0.55 mmol), acetic acid (0.04 ml, 0.69 mmol) and sodium triacetoxyborohydride (193.72 mg, 0.91 mmol). 1H NMR (400 MHz, DMSO-d6) d 11.61 - 12.22 (br. s, 1H), 7.56 (d, J = 0.75 Hz, 1H), 7.14 - 7.29 (m, 2H), 7.01 (t,

J = 8.16 Hz, 2H), 6.94 (s, 1H), 6.66 (s, 1H), 6.59 (br. s., 1H), 6.55 (d, J = 8.28 Hz, 1H), 6.39 (d, J = 7.53 Hz, 1H), 4.28 (s, 2H), 3.89 (t, J = 5.77 Hz, 2H), 3.46 - 3.58 (m, 3H), 2.82 (s, 3H), 1.06 (d, J = 6.02 Hz, 6H). 19F / COSY / HSQC NMR (analysis performed on sample 0501B) consistent with desired product. LCMS long method, rt = 1.69 min, m/z [M+H]+ 411.2.

Procedure 20 (ESC1002314 to ESC1002327, ESC1002337 to ESC1002341 and ESC1002422 to ESC1002424)

Suzuki Coupling: To a microwave vial was added the aryl bromide, the desired boronic acid the solvents (dioxane+water) and the mixture was bubbled with argon for ~5 min. Then the cat mix first choice was added to the reaction mixture and the vessel was sealed before heating it to 80°C overnight . The vial was let to cool to rt then, plugged on silica gel eluting it with EtOAc. The solvents were removed via a Genevac then the mixture was purified by HPLC (acidic). The desired fraction were collected and re-purified if necessary. The mixtures were further dried overnight in a Vac oven (4 mbar, @50°C)

ESC1002422, 1H NMR (373K, 400 MHz, DMSO-d6): 7.4 (1H, Ar-H, d, J= 5.2Hz); 7.37-7.31 (1H, Ar-H, m); 7.29-7.21 (2H, Ar-H, m); 7.16-7.11 (1H, Ar-H, m); 7.09-7.03 (1H, Ar-H, m); 6.83 (1H, Ar-H, d, J= 4.9Hz); 5.09 (1H, CH, dd, J= 7.9, 5.2Hz); 3.82-3.74 (1H, CH2, m); 3.69-3.60 (1H, CH2, m); 2.18-2.10 (1H, CH2, m); 2.09 (3H, CH3, s); 2.03-1.90 (2H, CH2, m); 1.86-1.76 (1H, CH2, m); 1.75-1.66 (1H, CH2, m); 1.02-0.96 (2H, CH2, m); 0.75-0.70 (2H, CH2, m); LCMS long method, rt = 2.90 min, m/z [M+H]+ 388.2.

ESC1002423, 1H NMR (373K, 400 MHz, DMSO-d6): 7.43-7.39 (1H, Ar-H, m); 7.37 (1H, Ar-H, dd, J= 7.5, 1.6Hz); 7.32-7.23 (2H, Ar-H, m); 7.21 (2H, Ar-H, d, J= 7.2Hz); 7.09 (1H, Ar-H, dd, J= 7.5, 1.4Hz); 6.84 (1H, Ar-H, d, J= 4.9Hz); 5.03 (1H, CH, dd, J= 8.3, 5.2Hz); 3.84-3.76 (1H, CH2, m); 3.69-3.62 (1H, CH2, m); 2.21-2.11 (1H, CH2, m); 2.09 (3H, CH3, s); 2.03-1.91 (1H, CH2, m); 1.88-1.68 (2H, CH2, m). LCMS long method, rt = 2.71 min, m/z [M+H]+ 366.2.

ESC1002424, 1H NMR (373K, 400 MHz, DMSO-d6): 7.63 (1H, Ar-H, s); 7.41 (1H, Ar-H, s); 7.38 (1H, Ar-H, d, J= 5.0Hz); 7.30-7.22 (2H, Ar-H, m); 7.21-7.17 (2H, Ar-H, m); 6.82 (1H, Ar-H, d, J= 5.1Hz); 5.32 (1H, CH, dd, J= 8.0, 4.7Hz); 3.91(3H, CH3, s); 3.85-3.78 (1H, CH2, m); 3.72-3.64 (1H, CH2, m); 2.38-2.28 (1H, CH2, m); 2.13 (3H, CH3, s); 2.04-1.84 (2H, CH2, m); 1.79-1.69 (1H, CH2, m); LCMS long method, rt = 2.20 min, m/z [M+H]+ 352.2.

ESC1002420 and ESC1002421 were obtained by SFC chiral separation of ESC1002338.

1H NMR (373K, 400 MHz, DMSO-d6): 7.41-7.22 (5H, Ar-H, m); 7.09 (1H, Ar-H, dd, J= 7.6, 1.4Hz); 6.94 (1H, Ar-H, ddd, J= 8.3, 2.6, 1.1Hz);, 6.83 (1H, Ar-H, d, J= 5.1Hz); 6.79-6.71 (2H,

Ar-H, m); 5.0 (1H, CH, dd, J= 7.7, 4.9Hz); 3.82-3.75 (1H, CH2, m); 3.81 (3H, CH3, s); 3.69-3.61 (1H, CH2, m); 2.19-2.10 (1H, CH2, m); 2.10 (3H, CH3, s); 2.02-1.90 (1H, CH2, m); 1.87-1.69 (2H, CH2, m). LCMS long method, rt = 2.83 min, m/z [M+H]+ 378.2.

ESC1002766 and ESC1002767 were obtained by SFC chiral separation of ESC1002324.

ESC1002768 and ESC1002769 were obtained by SFC chiral separation of ESC1002321.

Procedure 21 (ESC1002334)

N-[2-(dimethylamino)ethyl]-2-fluoro-N-[3-(methylamino)phenyl]benzamide (35 mg, 0.11 mmol) and 1H-imidazole-5-carbaldehyde (12.8 mg, 0.13 mmol) were combined in DCM (2 mL) and acetic acid (0.01 mL, 0.17 mmol) was added. The mixture was stirred at room temperature for 20 minutes then sodium triacetoxyborohydride (47.04 mg, 0.22 mmol) was added and stirring was continued for 16 hours (LCMS). Additional 1H-imidazole-5-carbaldehyde (5.33 mg, 0.06 mmol) was added and stirring continued for 24 hours (LCMS). The reaction mixture was diluted with DCM (15 mL) and washed with NaOH (1M aq., 10 mL). The aqueous wash was extracted with DCM (10 mL) then organics were combined, washed with brine, dried over sodium sulphate, filtered and solvent was evaporated under reduced pressure to afford crude product, 42 mg. Purification by prep. HPLC (basic, late focussed gradient) followed by evaporation of solvent from the appropriate fractions and drying under vacuum afforded product as a colourless gum, 20 mg (45.6%) of N-[2-(dimethylamino)ethyl]-2-fluoro-N-[3-[1H-imidazol-4-ylmethyl(methyl)amino]phenyl]benzamide. 1H NMR (400 MHz, DMSO-d6) d 11.69 - 11.97 (m, 1H), 7.54 (s, 1H), 7.19 (m, 2H), 7.00 (m, 3H), 6.65 (br. s., 1H), 6.51 - 6.60 (m, 2H), 6.37 (d, J = 7.28 Hz, 1H), 4.28 (s, 2H), 3.87 (t, J = 6.78 Hz, 2H), 2.83 (s, 3H), 2.40 (t, J = 6.65 Hz, 2H), 2.16 (s, 6H). COSY NMR consistent with product. LCMS long method, rt = 1.13 min, m/z [M+H]+ 394.2.

Procedure 22 (ESC1002344)

N-(cyclopropylmethyl)-2-fluoro-N-[3-[methyl-[[1-(2-trimethylsilylethoxymethyl)imidazol-4-yl]methyl]amino] phenyl]benzamide (20 mg, 0.04 mmol) was dissolved in MeOH (0.25 mL) and THF (0.25 mL) and HCl (5M aq., 0.2 mL) was added. The reaction was heated to reflux for 24 hours. The reaction mixture was concentrated under reduced pressure and the resulting residue dissolved in DCM and washed with saturated sodium bicarbonate solution. Organics were filtered through a hydrophobic frit and solvent was evaporated under reduced pressure to afford crude product. Purification by prep. HPLC (basic, late focussed gradient method) afforded two major products (largest peak due to boronate complex impurity brought through from previous reaction, structure as shown). Evaporation of solvent from the appropriate

fractions and drying afforded desired product, 3.2 mg (21.5%) of N-(cyclopropylmethyl)-2-fluoro-N-[3-[1H-imidazol-4-ylmethyl(methyl)amino]phenyl]benzamide. 1H NMR (373K, 400 MHz, CDCl3): 7.87-7.84 (2H, Ar-H, m); 7.76-7.71 (2H, Ar-H, m); 7.33-7.27 (1H, Ar-H, m); 7.23-7.15 (2H, Ar-H, m); 7.07-6.99 (2H, Ar-H, m); 6.92-6.76 (2H, Ar-H, m); 3.80-3.73 (2H, CH2, m); 3.66 (2H, CH2, t, J= 6.5Hz); 3.33-3.24 (2H, CH2, m); 2.88 (3H, CH3, s); 1.96-1.78 (3H, CH+CH2, m); 1.25 (1H, NH+, s); 0.96 (6H, (CH3)2, d, J= 6.3Hz). LCMS long method, rt = 2.93 min, m/z [M+H]+ 488.2.

Procedure 23 (ESC1002344)

To a flask were added 2-fluoro-N-isobutyl-N-[3-(methylamino)phenyl]benzamide (100 mg, 0.33 mmol), 2-(3-bromopropyl)isoindoline-1,3-dione (100 mg, 0.37 mmol), K_2CO_3 (138.03 mg, 1 mmol) and diluted in acetonitrile (15 mL). The mixture was refluxed for 5 days. A portion of NaH in mineral oil was added and the mixture was stirred at rt for 6h. The mixture was quenched with water and extracted with EtOAc (3 x 25mL). The organic layers were dried over Na_2SO_4 and reduced before being charged on a 10 g silica cartridge and eluted with Hep:EtOAc (0-50%). Although separation occurred it wasn't clean therefore the mixture was purified on HPLC (acidic). The desired fractions were collected and reduced to obtain 10g of the desired compound. Due to the greasy character, it was decided to salt the compound by diluting it in 2 mL of Ether and adding 0.1 mL of 3M HCl in dioxane. The mixture was reduced to form a clear white solid. 1H NMR (373K, 400 MHz, CDCl3): 7.87-7.84 (2H, Ar-H, m); 7.76-7.71 (2H, Ar-H, m); 7.33-7.27 (1H, Ar-H, m); 7.23-7.15 (2H, Ar-H, m); 7.07-6.99 (2H, Ar-H, m); 6.92-6.76 (2H, Ar-H, m); 3.80-3.73 (2H, CH2, m); 3.66 (2H, CH2, t, J= 6.5Hz); 3.33-3.24 (2H, CH2, m); 2.88 (3H, CH3, s); 1.96-1.78 (3H, CH+CH2, m); 1.25 (1H, NH+, s); 0.96 (6H, (CH3)2, d, J= 6.3Hz). LCMS long method, rt = 2.93 min, m/z [M+H]+ 488.2.

Procedure 24 (ESC1002399)

N-(cyclopropylmethyl)-4-methyl-N-[3-[methyl-[[1-(2-trimethylsilylethoxymethyl)imidazol-4-yl]methyl]amin o]phenyl]benzamide (27 mg, 0.05 mmol) was dissolved in MeOH (1 mL) and THF (1 mL) and 5M HCl (1 mL) was added. The reaction was heated in a microwave to 150°C for 30 minutes. The reaction mixture was concentrated under reduced pressure and the resulting brown oil partitioned between DCM and saturated sodium bicarbonate solution. Organics were filtered through a hydrophobic frit and solvent was evaporated under reduced pressure to afford crude product (18mg). Purification by prep. HPLC (basic, late focussed gradient) followed by evaporation of solvent from the appropriate fractions afforded product as a pale yellow gum, 2.6 mg (13%) of N-(cyclopropylmethyl)-N-[3-[1H-imidazol-4-ylmethyl(methyl)amino]phenyl]-4-methyl-benzamide. 1H NMR (400 MHz, DMSO-d6) d 12.66 - 13.13 (br. s, 1H), 7.94 (s, 1H), 7.14 (d, J = 8.03 Hz, 2H), 6.94 - 7.02 (m, 3H), 6.86 (s, 1H),

6.59 (d, J = 2.01 Hz, 2H), 6.24 - 6.39 (m, 1H), 4.37 (s, 2H), 3.65 (d, J = 7.03 Hz, 2H), 2.87 (s, 3H), 2.22 (s, 3H), 0.90 - 1.05 (m, 1H), 0.36 (dd, J = 1.51, 8.03 Hz, 2H), 0.09 (d, J = 4.77 Hz, 2H). COSY NMR consistent with product. LCMS long method, rt = 1.76 min, m/z [M+H]+ 375.2.

Procedure 25 (ESC1002125 to ESC1002437, ESC1002624 to ESC1002627, ESC1002661 to ESC1002666)

Amide coupling: to a vial were added 2-[2-(3-methoxyphenyl)phenyl]pyrrolidine (50.67 mg, 0.2 mmol), the acid, dichloromethane (4 mL), DIEA (0.09 ml, 0.5 mmol) and O-(7-Azabenzotriazol-1-yl)-N,N,N',N'-tetramethyluronium hexafluorophosphate (114.07 mg, 0.3 mmol) then the mixture was stirred at rt overnight. The solvents were removed (Genevac) then purified on HPLC (prep acidic). The desired fractions were collected and dried (Genevac). The products were transferred to a vial then dried over Vac oven (50°C, 3 mbar) for 72h. NB: Most compounds present a very blurry ^{1}H NMR spectra due to rotameric effects. A high temp NMR was therefore carried out. Some compounds presented clear spectra at rt as a mixture of diastereoisomers, but upon heating became blurry.

ESC1002754 and ESC1002755 were obtained by SFC chiral separation of ESC1002666.

ESC1002756 and ESC1002757 were obtained by SFC chiral separation of ESC1002626.
1H NMR (373K, 400 MHz, DMSO-d6): 13.02 (1H, NH, br); 7.74 (1H, Ar-H, br); 7.39-6.82 (8H, Ar-H, m); 5.37-5.25 (1H, CH, m); 3.90-3.78 (1H, CH2, m); 3.82 (3H, CH3, s); 3.72-3.64 (1H, CH2, m); 2.12-2.02 (1H, CH2, m); 1.96-1.85 (1H, CH2, m); 1.84-1.74 (1H, CH2, m); 1.72-1.63 (1H, CH2, m); LCMS long method, rt = 2.33 min, m/z [M+H]+ 382 .2.

Procedure 26 (ESC1002454)

2,4-difluoro-N-isobutyl-N-[3-[methyl-[[1-(2-trimethylsilylethoxymethyl)imidazol-4-yl]methyl]amino]phenyl] benzamide (37 mg, 0.07 mmol) was dissolved in MeOH (1 m\L) and 5M HCl (0.5 mL) added. The reaction was heated to reflux for 6 hours then stirred at room temperature for 16 hours, overnight. Heating was continued for 5 hours (LCMS) then the reaction mixture was concentrated under reduced pressure. The crude HCl salt was dissolved in DCM (2 mL) and washed with saturated sodium bicarbonate solution (2ml). The aqueous wash was re-extracted with DCM (2 mL) then organics were combined, filtered through a hydrophobic frit and solvent was evaporated under reduced pressure to afford crude product, 27mg. Purification by prep. HPLC (basic, late focussed gradient method) followed by evaporation of solvent from the appropriate fractions and drying afforded product, 15 mg (53.8%) of 2,4-difluoro-N-[3-[1H-imidazol-4-ylmethyl(methyl)amino]phenyl]-N-isobutyl-

benzamide. 1H NMR (400 MHz, DMSO-d6) d 11.89 (br. s., 1H), 7.55 (s, 1H), 7.25 (m, 1H), 7.07 (m, 1H), 6.93 - 7.01 (m, 1H), 6.84 - 6.93 (m, 1H), 6.72 (s, 1H), 6.50 - 6.61 (m, 2H), 6.34 (d, J = 7.53 Hz, 1H), 4.29 (s, 2H), 3.65 (d, J = 7.28 Hz, 2H), 2.86 (s, 3H), 1.63 - 1.80 (m, 1H), 0.89 (d, J = 6.53 Hz, 6H). COSY / 19F NMR consistent with product. LCMS long method, rt = 1.74 min, m/z [M+H]+ 399.2.

<u>ESC1002455</u>

Procedure 26 with 2-fluoro-N-isobutyl-N-[3-[methyl-[[1-(2-trimethylsilylethoxymethyl)imidazol-4-yl]methyl]amino]phenyl]-4-(t rifluoromethyl)benzamide (73%, 43 mg, 0.05 mmol) in place of 2,4-difluoro-N-isobutyl-N-[3-[methyl-[[1-(2-trimethylsilylethoxymethyl)imidazol-4-yl]methyl]amino]phenyl] benzamide. The product was obtained as 13 mg (53.4%) of 2-fluoro-N-[3-[1H-imidazol-4-ylmethyl(methyl)amino]phenyl]-N-isobutyl-4-(trifluoromethyl)benzamide. 1H NMR (400 MHz, DMSO-d6) d 11.89 (br. s., 1H), 7.52 - 7.59 (m, 2H), 7.38 - 7.49 (m, 2H), 6.97 (t, J = 8.03 Hz, 1H), 6.74 (s, 1H), 6.52 - 6.63 (m, 2H), 6.36 (d, J = 7.53 Hz, 1H), 4.29 (s, 2H), 3.68 (d, J = 7.28 Hz, 2H), 2.86 (s, 3H), 1.72 (td, J = 6.87, 13.61 Hz, 1H), 0.91 (d, J = 6.53 Hz, 6H). 19F / COSY NMR consistent with product. LCMS long method, rt = 1.92 min, m/z [M+H]+ 449.2.

<u>ESC1002456</u>

Procedure 26 with 2,6-difluoro-N-isobutyl-N-[3-[methyl-[[1-(2-trimethylsilylethoxymethyl)imidazol-4-yl]methyl]amino]phenyl] benzamide (25 mg, 0.05 mmol) in place of 2,4-difluoro-N-isobutyl-N-[3-[methyl-[[1-(2-trimethylsilylethoxymethyl)imidazol-4-yl]methyl]amino]phenyl] benzamide. The product was obtained as 8 mg (42.5%) of 2,6-difluoro-N-[3-[1H-imidazol-4-ylmethyl(methyl)amino]phenyl]-N-isobutyl-benzamide. 1H NMR (400 MHz, DMSO-d6) d 11.86 (br. s., 1H), 7.54 (s, 1H), 7.29 (m, 1H), 7.00 (t, 1H), 6.89 - 6.97 (m, 2H), 6.58 - 6.67 (m, 2H), 6.53 (s, 1H), 6.40 (d, 1H), 4.29 (s, 2H), 3.65 (d, J = 7.28 Hz, 2H), 2.86 (s, 3H), 1.70 (s, 1H), 0.90 (d, J = 6.78 Hz, 6H). 19F / COSY NMR consistent with product. LCMS long method, rt = 1.80 min, m/z [M+H]+ 399.2.

<u>ESC1002457</u>

Procedure 26 with 2-chloro-N-isobutyl-N-[3-[methyl-[[1-(2-trimethylsilylethoxymethyl)imidazol-4-yl]methyl]amino]phenyl]ben zamide (86%, 33 mg, 0.05 mmol) in place of 2,4-difluoro-N-isobutyl-N-[3-[methyl-[[1-(2-trimethylsilylethoxymethyl)imidazol-4-yl]methyl]amino]phenyl] benzamide. The product was obtained as 9 mg (42.1%) of 2-chloro-N-[3-[1H-imidazol-4-ylmethyl(methyl)amino]phenyl]-N-isobutyl-benzamide. 1H NMR (400 MHz, DMSO-d6) d 11.76 - 12.01 (br. s, 1H), 7.54 (s, 1H), 7.29 (d, J = 7.78 Hz, 1H), 7.06 - 7.22 (m, 3H), 6.91 - 6.98 (m, 1H), 6.65 (s, 1H), 6.59 (s, 1H), 6.60 (d, J = 1.76 Hz, 1H), 6.49 - 6.55 (m, 1H), 6.44 (d, J = 7.53

Hz, 1H), 4.28 (s, 2H), 3.67 (br. s., 2H), 2.84 (s, 3H), 1.69 (d, J = 6.78 Hz, 1H), 0.91 (d, J = 6.78 Hz, 6H). COSY NMR consistent with product. LCMS long method, rt = 1.76 min, m/z [M+H]+ 397.4, 399.2, Cl splitting pattern.

ESC1002459

Procedure 26 with 4-ethyl-N-isobutyl-N-[3-[methyl-[[1-(2-trimethylsilylethoxymethyl)imidazol-4-yl]methyl]amino]phenyl]benzamide (83%, 34 mg, 0.05 mmol) in place of 2,4-difluoro-N-isobutyl-N-[3-[methyl-[[1-(2-trimethylsilylethoxymethyl)imidazol-4-yl]methyl]amino]phenyl] benzamide. The product was obtained as 13.5 mg (63.8%) of 4-ethyl-N-[3-[1H-imidazol-4-ylmethyl(methyl)amino]phenyl]-N-isobutyl-benzamide. 1H NMR (400 MHz, DMSO-d6) d 11.78 - 12.23 (m, 1H), 7.57 (d, J = 0.75 Hz, 1H), 7.13 (d, J = 8.28 Hz, 2H), 7.01 (d, J = 8.03 Hz, 2H), 6.95 (t, J = 8.03 Hz, 1H), 6.72 (s, 1H), 6.49 - 6.59 (m, 2H), 6.27 (d, J = 7.53 Hz, 1H), 4.29 (s, 2H), 3.66 (d, J = 7.28 Hz, 2H), 2.84 (s, 3H), 2.50 (m, 2H), 1.65 - 1.85 (m, 1H), 1.10 (t, J = 7.65 Hz, 3H), 0.86 (d, J = 6.53 Hz, 6H). COSY NMR consistent with desired product. LCMS long method, rt = 1.87 min, m/z [M+H]+ 391.2.

ESC1002460

Procedure 26 with 5-chloro-N-isobutyl-N-[3-[methyl-[[1-(2-trimethylsilylethoxymethyl)imidazol-4-yl]methyl]amino]phenyl]thio phene-2-carboxamide (82%, 35 mg, 0.05 mmol) in place of 2,4-difluoro-N-isobutyl-N-[3-[methyl-[[1-(2-trimethylsilylethoxymethyl)imidazol-4-yl]methyl]amino]phenyl] benzamide. The product was obtained as 16.2 mg (74.7%) of 5-chloro-N-[3-[1H-imidazol-4-ylmethyl(methyl)amino]phenyl]-N-isobutyl-thiophene-2-carboxamide. 1H NMR (400 MHz, DMSO-d6) d 11.79 - 11.98 (br. s, 1H), 7.52 (d, J = 1.00 Hz, 1H), 7.20 (t, J = 8.03 Hz, 1H), 6.89 (d, J = 4.27 Hz, 1H), 6.85 (dd, J = 2.13, 8.41 Hz, 1H), 6.80 (br. s., 1H), 6.75 (d, J = 1.76 Hz, 1H), 6.50 (d, J = 7.53 Hz, 1H), 6.44 (d, J = 4.02 Hz, 1H), 4.40 (s, 2H), 3.58 (d, J = 7.28 Hz, 2H), 2.97 (s, 3H), 1.63 - 1.84 (m, 1H), 0.87 (d, J = 6.53 Hz, 6H). COSY NMR consistent with product.LCMS short method, rt = 1.84 min, m/z [M+H]+ 403.2, 405.4 (chloride splitting pattern).

Procedure 27 (ESC1002462 and ESC1002698 to ESC1002702 and ESC1002704)

To a microwave vial were added [2-(2-bromophenyl)pyrrolidin-1-yl]-(3-methyl-2-thienyl)methanone (80 mg, 0.23 mmol) (or generally the desired aryl bromide), phenylboronic acid (55.7 mg, 0.46 mmol) (or generally the desired boronic acid) the solvents (dioxane+water) then the mixture was bubbled with argon for ~5 min. Catkit first choice was added to the reaction mixture and the vessel was sealed before heating it to 80°C overnight. Once completed the mixture was diluted with EtOAc and water and extracted with EtOAc (3 x 15 mL). The org. layers are dried over Na_2SO_4, and reduced before charging the mixture on a 10

g silica cartridge. The mixture was purified on an Isolera and the desired fractions were collected. The solids obtained were further dried in a vac. oven (@50°C overnight).The compounds was analysed by ^{1}H NMR at 373K to resolve rotameric effects. ESC1002462, 1H NMR (373K, 400 MHz, DMSO-d6): 7.45-7.34 (5H, Ar-H, m); 7.31-7.24 (2H, Ar-H, m); 7.20-7.15 (2H, Ar-H, m); 7.09 (1H, Ar-H, dd, J= 7.5, 1.8Hz); 6.84 (1H, Ar-H, d, J= 5.0Hz); 5.08 (1H, CH, dd, J= 7.6, 5.0Hz), 3.83-3.75 (1H, CH2, m); 3.69-3.61 (1H, CH2, m); 2.18-2.10 (1H, CH2, m); 2.08 (3H, CH3, s); 2.02-1.91 (1H, CH2, m); 1.87-1.68 (2H, CH2, m); LCMS long method, rt = 2.68 min, m/z [M+H]+ 348.2.

Procedure 28 (ESC1002463)

To a microwave vial were added (3-methyl-2-thienyl)-[2-[2-(1H-pyrazol-4-yl)phenyl]pyrrolidin-1-yl]methanone (32 mg, 0.09 mmol), 2-bromoethanol (8.07 µL, 0.11 mmol), K_2CO_3 (19.66 mg, 0.14 mmol) and acetonitrile (2 ml). The vessel was sealed and the subjected to microwave radiation for 20 min @ 150 °C. 0.1 mL of 2-bromoethanol was added and the mixture was subjected to 150°C for 30 min. The mixture was subjected to 45 min then 3h @ 150°C. The mixture was filtered reduced and purified by HPLC (basic) the desired fraction were collected and reduced. The compound was further dried over Genevac (high bp) and vac oven (3mbar @52°C overnight). The compounds was analysed by ^{1}H NMR at 373K to resolve rotameric effects. 1H NMR (373K, 400 MHz, DMSO-d6): 7.67 (1H, Ar-H, s); 7.43 (1H, Ar-H, s); 7.37 (1H, Ar-H, d, J= 5.1Hz); 7.30-7.17 (4H, Ar-H, m); 6.81 (1H, Ar-H, d, J= 4.9Hz); 5.32 (1H, CH, dd, J= 8.0, 4.3Hz), 4.57 (1H, OH, t, J= 5.4Hz); 4.22 (2H, CH2, t, J= 5.8Hz); 3.87-3.78 (3H, CH2, m); 3.73-3.65 (1H, CH2, m); 2.38-2.29 (1H, CH2, m); 2.12 (3H, CH3, s); 2.03-1.85 (2H, CH2, m); 1.79-1.70 (1H, CH2, m) LCMS long method, rt = 2.04 min, m/z [M+H]+ 382.2

Procedure 29 (ESC1002164 to ESC1002466)

Suzuki cross coupling method. To a vial microwave vial were added the aryl bromide, the desired boronic acid the solvents (dioxane+water) and then the mixture was bubbled with argon for ~5 min. Cat mix first choice was added to the reaction mixture and the vessel was sealed before heating it to 80°C for 16h. The mixture was allowed to cool to rt then diluted with EtOAc and Water and extracted with EtOAc (3 x 15 mL). The org. layers were filtered through celite, dried over Na_2SO_4, reduced. To the crude intermediate was added TFA (17.1 mg, 0.15 mmol) and dichloromethane (2 mL) and the mixture was stirred at rt for 3h. The mixtures were reduced (Genevac) then purified by HPLC prep. acidic (except reaction 4). the desired fractions were collected and reduced before flushing them through a 10 g silica cartridge on an Isolera using Hep:EtOAc (0-100%). the desired fractions were collected, reduced and dried over Geneva and vac oven (3 mbar @ 52°C).

ESC1002789 and ESC1002790 were obtained by SFC chiral separation of ESC1002320.

ESC1002491

Procedure 26 with N-isobutyl-2-methyl-N-[3-[methyl-[[1-(2-trimethylsilylethoxymethyl)imidazol-4-yl]methyl]amino]phenyl]benzamide (19 mg, 0.04 mmol) in place of 2,4-difluoro-N-isobutyl-N-[3-[methyl-[[1-(2-trimethylsilylethoxymethyl)imidazol-4-yl]methyl]amino]phenyl] benzamide. The product was obtained as 6.1 mg (43.2%) of N-[3-[1H-imidazol-4-ylmethyl(methyl)amino]phenyl]-N-isobutyl-2-methyl-benzamide. 1H NMR @ 298K (400 MHz, DMSO-d6) d 11.90 - 12.21 (br. s, 1H), 7.60 (s, 1H), 7.05 (br. s., 2H), 6.93 (br. s., 3H), 6.66 (s, 1H), 6.44 - 6.51 (m, 2H), 6.36 (d, J = 7.53 Hz, 1H), 4.25 (s, 2H), 3.70 (d, J = 7.03 Hz, 2H), 2.82 (s, 3H), 2.24 (s, 3H), 1.59 - 1.77 (m, 1H), 0.89 (d, J = 6.27 Hz, 6H). COSY NMR consistent with product. LCMS long method, rt = 1.66 min, m/z [M+H]+ 377.2.

ESC1002492

Procedure 26 with N-isobutyl-2,4-dimethyl-N-[3-[methyl-[[1-(2-trimethylsilylethoxymethyl)imidazol-4-yl]methyl]amino]phenyl] benzamide (13 mg, 0.02 mmol) in place of 2,4-difluoro-N-isobutyl-N-[3-[methyl-[[1-(2-trimethylsilylethoxymethyl)imidazol-4-yl]methyl]amino]phenyl] benzamide. The product was obtained as 3.9 mg (40%) of N-[3-[1H-imidazol-4-ylmethyl(methyl)amino]phenyl]-N-isobutyl-2,4-dimethyl-benzamide. 1H NMR 298K (400 MHz, DMSO-d6) d 11.92 - 12.31 (br. s, 1H), 7.62 (s, 1H), 6.78 - 7.00 (m, 3H), 6.71 (br. s., 2H), 6.48 (br. s., 2H), 6.33 (d, J = 6.53 Hz, 1H), 4.27 (br. s., 2H), 3.68 (d, J = 5.77 Hz, 2H), 2.84 (s, 3H), 2.08 - 2.25 (m, 6H), 1.68 (br. s., 1H), 0.88 (d, J = 5.77 Hz, 6H). COSY NMR consistent with product. LCMS long method, rt = 1.74 min, m/z [M+H]+ 391.4.

ESC1002493

Procedure 26 with N-isobutyl-N-[3-[methyl-[[1-(2-trimethylsilylethoxymethyl)imidazol-4-yl]methyl]amino]phenyl]-2-(trifluorom ethyl)benzamide (24 mg, 0.04 mmol) in place of 2,4-difluoro-N-isobutyl-N-[3-[methyl-[[1-(2-trimethylsilylethoxymethyl)imidazol-4-yl]methyl]amino]phenyl] benzamide. The product was obtained as 4.6 mg (25%) of N-[3-[1H-imidazol 4-ylmethyl(methyl)amino]phenyl]-N-isobutyl-2-(trifluoromethyl)benzamide. 1H NMR 298K (400 MHz, DMSO-d6) d 11.87 - 12.15 (br. s, 1H), 7.56 - 7.65 (m, 2H), 7.39 (t, J = 5.77 Hz, 2H), 7.20 (d, J = 6.78 Hz, 1H), 6.96 (t, J = 8.03 Hz, 1H), 6.66 (s, 1H), 6.48 - 6.58 (m, 2H), 6.37 (d, J = 7.78 Hz, 1H), 4.28 (s, 2H), 3.53 - 3.88 (m, 2H), 2.83 (s, 3H), 1.62 - 1.77 (m, 1H), 0.88 (d, J = 6.78 Hz, 6H). COSY / F19 NMR consistent with product. LCMS long method, rt = 1.73 min, m/z [M+H]+ 431.2.

Procedure 30 (ESC1002504 to ESC1002506 and ESC1002597)

Amine and the desired halogen was added to a flask, and the mixture was diluted in tetrahydrofuran (2 ml) and TEA (0.07 mL, 0.5 mmol) and the mixture was heated to 70°C overnight. LCMS revealed >50% conversion. The mixture was diluted in EtOAc and sat $NaHCO_3$ solution then extracted with EtOAc (3 x 3mL). The org. phases were dried over Na_2SO_4 and reduced before being charged on a 10g silica cartridge and purified on an Isolera (Hep:EtOAc 0-50%). The purification was repeated. The compound was further purified on HPLC and the desired fractions were dried. The paste was slated using 0.1 mL of 4M HCl in Dioxane, and the compound was dried before submission (Genevac and vac oven 3 mbar @ 52°C).

ESC1002597, 1H NMR (400 MHz, DMSO-d6) d 12.12 (br. s., 1H), 7.63 (s, 1H), 7.34 (d, J = 4.77 Hz, 1H), 7.03 (t, J = 8.03 Hz, 1H), 6.68 - 6.74 (m, 2H), 6.61 (dd, J = 2.13, 8.41 Hz, 1H), 6.47 - 6.52 (m, 1H), 6.35 - 6.41 (m, 1H), 4.30 (s, 2H), 3.65 (d, J = 7.53 Hz, 2H), 2.86 (s, 3H), 2.11 (s, 3H), 1.64 - 1.80 (m, 1H), 0.86 (d, J = 6.78 Hz, 6H). COSY NMR consistent with product. LCMS long method, rt = 1.77 min, m/z [M+H]+ 383.2.

Procedure 31 (ESC1002507)

N-[3-[3-aminopropyl(methyl)amino]phenyl]-2-fluoro-N-isobutyl-benzamide (40 mg, 0.11 mmol) was added to a flask, Acetic anhydride (0.02 mL, 0.25 mmol) and Pyridine (2 mL) as solvent. The mixture was stirred at rt for 3h. The mixture was reduced and purified by a prep. HPLC, under basic conditions. The obtained compound was salted using HCl in dioxane (0.1 mL) and further reduced.

ESC1002508

Procedure 26 with N-butyl-2-fluoro-N-[3-[methyl-[[1-(2-trimethylsilylethoxymethyl)imidazol-4-yl]methyl]amino]phenyl]benzamide (64 mg, 0.13 mmol) in place of 2,4-difluoro-N-isobutyl-N-[3-[methyl-[[1-(2-trimethylsilylethoxymethyl)imidazol-4-yl]methyl]amino]phenyl] benzamide. The product was obtained as 18 mg (37.8%) of N-butyl-2-fluoro-N-[3-[1H-imidazol-4-ylmethyl(methyl)amino]phenyl]benzamide. 1H NMR (400 MHz, DMSO-d6) d 11.88 - 12.14 (br. s, 1H), 7.59 (s, 1H), 7.21 (td, J = 6.62, 13.62 Hz, 2H), 6.90 - 7.06 (m, 3H), 6.68 (s, 1H), 6.47 - 6.59 (m, 2H), 6.35 (d, J = 7.53 Hz, 1H), 4.28 (s, 2H), 3.78 (t, J = 7.15 Hz, 2H), 2.83 (s, 3H), 1.39 - 1.53 (m, 2H), 1.22 - 1.38 (m, 2H), 0.88 (t, J = 7.28 Hz, 3H). COSY NMR consistent with product. LCMS long method, rt = 1.76 min, m/z [M+H]+ 381.2.

ESC1002516

Procedure 26 with 2-fluoro-N-[(5-methylisoxazol-3-yl)methyl]-N-[3-[methyl-[[1-(2-trimethylsilylethoxymethyl)imidazol-4-yl]m ethyl]amino]phenyl]benzamide (20 mg, 0.04 mmol)

in place of 2,4-difluoro-N-isobutyl-N-[3-[methyl-[[1-(2-trimethylsilylethoxymethyl)imidazol-4-yl]methyl]amino]phenyl] benzamide. Purification by prep. HPLC (acidic method) and SCX, product eluted with 2M methanolic ammonia followed by evaporation of solvent and drying afforded product as 5 mg (32.8%) of 2-fluoro-N-[3-[1H-imidazol-4-ylmethyl(methyl)amino]phenyl]-N-[(5-methylisoxazol-3-yl)methyl]benzamide. Trace MeOH impurity, but sufficiently pure for submission for testing. 1H NMR (400 MHz, DMSO-d6) d 11.88 - 12.19 (br. s, 1H), 7.59 (s, 1H), 7.29 (br. s., 2H), 7.00 - 7.10 (m, 2H), 6.92 (s, 1H), 6.66 (s, 1H), 6.49 - 6.57 (m, 2H), 6.31 (d, J = 7.53 Hz, 1H), 6.20 (s, 1H), 5.00 (s, 2H), 4.24 (s, 2H), 2.79 (s, 3H), 2.39 (s, 3H). COSY NMR consistent with product. LCMS long method, rt = 1.54 min, m/z [M+H]+ 420.2.

<u>ESC1002517</u>

Procedure 26 with 2 N-(2,2-dimethylpropyl)-2-fluoro-N-[3-[methyl-[[1-(2-trimethylsilylethoxymethyl)imidazol-4-yl]methyl]amino]phenyl]benzamide (26 mg, 0.05 mmol) in place of 2,4-difluoro-N-isobutyl-N-[3-[methyl-[[1-(2-trimethylsilylethoxymethyl)imidazol-4-yl]methyl]amino]phenyl] benzamide. Product obtained as 11.4 mg (58.3%) of N-(2,2-dimethylpropyl)-2-fluoro-N-[3-[1H-imidazol-4-ylmethyl(methyl)amino]phenyl]benzamide. 1H NMR (400 MHz, DMSO-d6) d 11.82 (br. s., 1H), 7.53 (s, 1H), 7.18 - 7.29 (m, 1H), 7.11 (m, 1H), 7.00 (m, 2H), 6.90 (m, 1H), 6.63 (s, 1H), 6.56 (br. s., 1H), 6.33 - 6.54 (m, 2H), 4.25 (s, 2H), 3.79 (s, 2H), 2.71 - 2.91 (m, 3H), 0.82 (s, 9H). Restricted rotation at 298K confirmed by high T NMR at 373K. COSY NMR consistent with product. LCMS long method, rt = 1.83 min, m/z [M+H]+ 395.2.

<u>Procedure 32 (ESC1002575)</u>

2-fluoro-N-isobutyl-4-methyl-N-[3-[methyl-[[1-(2-trimethylsilylethoxymethyl)imidazol-4-yl]methyl]amino]phenyl]benzamide (39 mg, 0.07 mmol) was dissolved in MeOH (1 mL) and 5M HCl (0.5 mL) added. The solution was heated to 70°C for 18 hours. The reaction mixture was basified with addition of NaOH (2M aq.) and extracted with DCM (2 x 25 mL). Organics were combined, dried over sodium sulphate, filtered and solvent was evaporated under reduced pressure to afford crude product, 30mg. LCMS indicated recovered starting material (suggests HCl also evaporated during reaction). The recovered material was dissolved in MeOH (1 mL) and HCl (5M, 0.5 mL) added. The reaction was heated to 70°C for 18 hours (LCMS) then concentrated in a Genevac. The resulting residue was dissolved in DCM (2 mL) and washed with saturated sodium bicarbonate solution (2 mL). The wash was re-extracted with DCM (2 mL) then organics were combined, filtered through a hydrophobic frit and solvent was evaporated in a Genevac. The crude residue was purified by prep. HPLC (basic, late focussed gradient) followed by evaporation of solvent and drying to afford product, 11 mg

(37.5%) of 2-fluoro-N-[3-[1H-imidazol-4-ylmethyl(methyl)amino]phenyl]-N-isobutyl-4-methyl-benzam. 1H NMR (400 MHz, DMSO-d6) d 11.88 (br. s., 1H), 7.55 (d, J = 0.75 Hz, 1H), 7.04 (m, 1H), 6.95 (m, 1H), 6.77 - 6.88 (m, 2H), 6.69 (br. s., 1H), 6.53 (br. s., 2H), 6.33 (d, J = 7.28 Hz, 1H), 4.28 (s, 2H), 3.64 (d, J = 7.03 Hz, 2H), 2.85 (s, 3H), 2.20 (s, 3H), 1.60 - 1.81 (m, 1H), 0.88 (d, J = 6.53 Hz, 6H). COSY NMR consistent with desired product.LCMS long method, rt = 1.81 min, m/z [M+H]+ 395.2.

<u>ESC1002614</u>
Procedure 26 with N-isobutyl-N-[3-[methyl-[[1-(2-trimethylsilylethoxymethyl)imidazol-4-yl]methyl]amino]phenyl]-2-oxo-1H-p yridine-3-carboxamide (7 mg, 0.01 mmol) in place of 2,4-difluoro-N-isobutyl-N-[3-[methyl-[[1-(2-trimethylsilylethoxymethyl)imidazol-4-yl]methyl]amino]phenyl] benzamide. Product obtained as 1.2 mg (23%) of N-[3-[1H-imidazol-4-ylmethyl(methyl)amino]phenyl]-N-isobutyl-2-oxo-1H-pyridine-3-carboxamide. 1H NMR (400 MHz, DMSO-d6) d 11.57 - 11.91 (m, 2H), 7.54 (s, 1H), 7.19 (d, J = 5.02 Hz, 1H), 7.11 (br. s., 1H), 6.97 (t, J = 7.78 Hz, 1H), 6.65 - 6.82 (m, 2H), 6.55 (d, J = 7.78 Hz, 1H), 6.44 (d, J = 7.53 Hz, 1H), 5.96 (br. s., 1H), 4.30 (br. s., 2H), 3.58 (d, J = 6.02 Hz, 2H), 2.86 (br. s., 3H), 1.66 (m, 1H), 0.87 (d, J = 6.53 Hz, 6H). COSY NMR consistent with product. LCMS long method, rt = 1.51 min, m/z [M+H]+ 380.0.

<u>ESC1002624</u>
Procedure 26 with N-isobutyl-2-methylsulfonyl-N-[3-[methyl-[[1-(2-trimethylsilylethoxymethyl)imidazol-4-yl]methyl]amino]ph enyl]benzamide (60 mg, 0.11 mmol) in place of 2,4-difluoro-N-isobutyl-N-[3-[methyl-[[1-(2-trimethylsilylethoxymethyl)imidazol-4-yl]methyl]amino]phenyl] benzamide. Product obtained as 29 mg (62.6%) of N-[3-[1H-imidazol-4 ylmethyl(methyl)amino]phenyl]-N-isobutyl-2-methylsulfonyl-benzamide. 1H NMR (373K, 400 MHz, DMSO-d6): 7.81 (1H, Ar-H, d, J= 5.1Hz); 7.40-7.31 (4H, Ar-H, m); 7.30-7.24 (1H, Ar-H, m); 7.12 (1H, Ar-H, d, J= 7.6Hz); 6.95 (1H, Ar-H, dd, J= 8.3, 2.7Hz); 6.80 (2H, Ar-H, br); 5.20-5.13 (1H, CH, m); 3.95-3.86 (1H, CH2, m); 3.82 (3H, CH3, s); 3.78-3.68 (1H, CH2, m); 2.27-2.15 (1H, CH2, m); 2.08-1.96 (1H, CH2, m); 1.93-1.76 (2H, CH2, m); LCMS long method, rt = 2.51 min, m/z [M+H]+ 389.2.

<u>Procedure 33 (ESC1002656)</u>
N-isobutyl-N-[3-[methyl-[[1-(2-trimethylsilylethoxymethyl)imidazol-4-yl]methyl]amino]phenyl]pyrazine-2-carboxamide (54 mg, 0.11 mmol) was dissolved in TBAF (1 M in THF, 2.5 mL) and the reaction heated to 120°C in a microwave for 60 minutes (LCMS). The reaction mixture was concentrated under reduced pressure and the afforded residue partitioned between DCM (10 mL) and 10% citric acid solution (10 mL). The acid solution was

separated (organics discarded) and made basic with addition of NaOH (2M aq.) then extracted with DCM (2 x 10 mL). Organics were combined, filtered through a hydrophobic frit and solvent was evaporated under reduced pressure to afford crude product, 95 mg. Purification by prep. HPLC afforded the desired product. The product was purified by flash chromatography (silica column, elution with isocratic 90:10:1 DCM:MeOH:NH_4OH). Evaporation of solvent from the appropriate fractions and drying afforded desired product as a pale yellow solid, 18 mg (45.2%) of N-[3-[1H-imidazol-4-ylmethyl(methyl)amino]phenyl]-N-isobutyl-pyrazine-2-carboxamide. 1H NMR (400 MHz, DMSO-d6) d 11.85 (br. s., 1H), 8.55 (br. s., 1H), 8.44 (d, J = 9.29 Hz, 2H), 7.53 (s, 1H), 6.95 (m, 1H), 6.70 (br. s., 1H), 6.46 - 6.59 (m, 2H), 6.33 (d, J = 6.27 Hz, 1H), 4.27 (br. s., 2H), 3.72 (d, J = 7.03 Hz, 2H), 2.83 (br. s., 3H), 1.65 - 1.85 (m, 1H), 0.75 - 1.01 (m, 6H). COSY NMR consistent with product. LCMS long method, rt = 1.54 min, m/z [M+H]+ 365.2.

Procedure 34 (ESC1002696 and ESC1002697)

To a solution of Reactant 0 (37 mg, 0.1 mmol) in 2-MeTHF was added 1 M $(CH_3)_2S \cdot BH_3$ in 2-MeTHF (0.29 mL) and the mixture was heated to reflux. After 16h the mixture was analysed by LCMS which indicated ~60% conversion. More 1 M $(CH_3)_2S \cdot BH_3$ in 2-MeTHF (0.5 mL) was added and the mixture was refluxed for a further 5h. The solution was allowed to cool to rt and quenched with 25 mL of 1M HCl solution. The crude was then extracted with EtOAc (3 x 20 mL), dried over Na_2SO_4 and reduced. The crude was first purified on prep HPCL (acidic conditions). With the desired fraction dried over a Genevac, the compound was charged on a 10 g silica cartridge and purified on an Isolera, using DCM:MeOH (0-10%) as eluant. The compound was then intensively dried on a Genevac then a Vac Oven (50°C, 3 mbar, >16h). ESC1002697, 1H NMR (373K, 400 MHz, DMSO-d6): 11.68 (1H, OH, br); 7.64 (1H, Ar-H, d, J= 4.8Hz); 7.45-7.31 (3H, Ar-H, m); 7.28 (1H, Ar-H, td, J= 7.4, 1.9Hz); 7.10 (1H, Ar-H, d, J= 8.1Hz); 6.97 (1H, Ar-H, d, J= 4.9Hz); 6.09 (1H, Ar-H, br); 6.00 (1H, Ar-H, br); 5.20-5.12 (1H, CH, m); 3.88-3.81 (1H, CH2, m); 3.67-3.60 (1H, CH2, m); 2.34-2.22 (1H, CH2, m); 2.06-1.95 (1H, CH2, m); 1.93-1.83 (1H, CH2, m); 1.82-1.72 (1H, CH2, m); LCMS long method, rt = 2.10 min, m/z [M+H]+ 385.2.

Procedure 35 (ESC1002696)

Reactant 1 (73 mg, 0.21 mmol), Reactant 2 (43.64 mg, 0.27 mmol), dichloromethane (5 ml), DIEA (0.09 mL, 0.52 mmol) and O-(7-Azabenzotriazol-1-yl)-N,N,N',N'-tetramethyluronium hexafluorophosphate (117.75 mg, 0.31 mmol) were added to a vial then the mixture was stirred at rt overnight. The mixture was partitioned with water and DCM and further extracted with DCM. The org phase was dried over a phase separator and reduced. The mixture was charged on a 10 g silica cartridge and purified on an Isolera (Hep:EtOAc 0-40%). The desired

fractions were collected dried and set to the following step. Product 1 (68 mg, 0.14 mmol) was diluted with Acetonitrile (9.5 mL) and water (0.5 mL), and the mixture was homogenised at rt. Then DBU (0.02 mL, 0.14 mmol) and the mixture was stirred at rt for 1h. DBU (0.1 mL) was added and the mixture was stirred for a further 2h. The mixture was partitioned between water and EtOAc and further extracted with EtOAc. The organic layers were dried over Na_2SO_4 then the mixture was reduced and purified on a prep HPLC (Acidic). The desired fractions were dried on a Genevac and charged on a 10 g silica cartridge and further purified on an Isolera using Hep:EtOAc (0-100%) as eluant. The desired fractions were collected and extensively dried over Genevac and Vac Oven (50°C, 3mbar, >16h). 1H NMR (373K, 400 MHz, DMSO-d6): 9.06 (1H, OH , br); 7.62 (1H, Ar-H, d, J= 4.9Hz); 7.34-7.18 (3H, Ar-H, m); 7.05 (1H, Ar-H, d, J= 7.2Hz); 7.00-6.86 (3H, Ar-H, m); 6.82 (1H, Ar-H, d, J= 8.5Hz); 5.16-5.09 (1H, CH, m); 3.86-3.78 (1H, CH2, m); 3.69-3.58 (1H, CH2, m); 2.22-2.12 (1H, CH2, m); 2.03-1.93 (1H, CH2, m); 1.87-1.80 (1H, CH2, m); 1.79-1.69 (1H, CH2, m); LCMS long method, rt = 2.15 min, m/z [M+H]+ 384.2.

Procedure 36 (ESC1002762)

To a solution of the appropriate N-Methylaniline analogue in toluene is added Cl-CH_2CN followed by K_2CO_3.The mixture is heated at reflux overnight. After cooling to RT The mixture is diluted with DCM and filtered. The filter residue is washed with DCM and the combined filtrates are evap. i.vac. The residual material is pre-purified on a 5 g Si-SPE column: Rf = 0.2 in heptane/$MeCO_2Et$ = 2/1 (unreacted starting material elutes slightly before with an Rf = 0.3). Final purification is carried out on basic prepHPLC where the desired product elutes between 8-8.4 min.

Procedure 37 (ESC1002764)

2-fluoro-{N}-[3-(1,2,4-triazol-4-yl)phenyl]benzamide (90.0 mg, 0.319 mmol) was dissolved in DMF (1 mL). Cs_2CO_3 (0.312 g, 0.957 mmol), NaI (0.00956 g, 0.0638 mmol) and 1-bromo-2-methyl-propane (0.104 mL) 0.957 mmol) were added to a vial. The vial was sealed and heated to 80°C for 3 hours. LCMS indicated that product was forming. Further 1-bromo-2-methyl-propane (0.208 mL) was added and the reaction heated for a further 3 hours. The reaction was diluted with ethyl acetate and water. The organic was washed with water, brine, dried over Na_2SO_4 and the solvent removed at reduced pressure. The resulting residue was purified by flash chromatography (5 g Biotage zip) eluting with a gradient 0 - 5% methanol in DCM. The appropriate fractions were combined, and the solvent removed at reduced pressure to afford product which was found to be impure. Product was further purified by acidic prep HPLC. The appropriate fractions were combined and the solvent removed in a Genevac to afford recovered starting amide and desired product.

Procedure 38 (ESC1002765)

2-fluoro-N-(3-imidazol-1-ylphenyl)benzamide (60 mg, 0.21 mmol) was stirred in DMF (1 mL). NaH disp. in mineral oil (60%, 12.8 mg, 0.32 mmol) was added. The reaction was placed under an argon atmosphere and stirred for 10 minutes. 1-bromo-2-methyl-propane (46.39 µL, 0.43 mmol) was added and the reaction heated to 60°C for 1 hour. Further 1-bromo-2-methyl-propane (46.39 µL, 0.43 mmol) was added and the reaction heated for a further 30 minutes. The reaction was diluted with ethyl acetate and quenched with water. The organic layer washed with water (x3), brine, dried over Na_2SO_4 and concentrated at reduced pressure. The resulting residue was purified by flash chromatography (5 g Biotage SiO_2 zip) elutiing with a gradient 0 - 95:5:05 DCM:MeOH:NH_4OH. The appropriate fractions were combined and the solvent removed at reduced pressure to afford product. 1H NMR (400 MHz, DMSO-d6) d 8.01 (br, 1H), 7.49 (d, 1H), 7.32 - 7.27 (m, 6H), 7.14 (m, 3H), 7.02 (t, 1H), 3.79 (d, 2H), 1.28 (m, 1H), 0.96 (d, 6H), LCMS: RT = 1.688, m/z 338.2 [M+H]+

Procedure 39 (ESC1002775)

Suzuki: (2-(2-bromophenyl)pyrrolidin-1-yl)(3-chlorothiophen-2-yl)methanone (0.1 g, 0.27 mmol) and (4-fluoro-3-hydroxyphenyl)boronic acid (63.09 mg, 0.4 mmol) were suspended in MeTHF (1 mL). First choice catkit ($Pd_2(dba)_3$, PCy_3, K_3PO_4 (135.96 mg, 0.27 mmol) was added to the reaction mixture which was heated to 150 °C for 20 min. The reaction mixture was filtered through celite which was washed with EtOAc. The EtOAc was washed with water, dried (Na_2SO_4), filtered and concentrated in vacuo. The resulting material was purified by HPLC (acidic prep.). The material was further purified by HPLC (base prep.). The relevant fractions were collected and concentrated in vacuo and dried in vacuo for 18 hours at 50 °C giving the desired compound as a white solid (28 mg, 26%). NMR experiments indicated that the material contains conformational isomers. 1H NMR (400 MHz, DMSO-d6, 100 °C): 9.48 (br. s., 1H), 7.58 - 7.68 (m, 1H), 7.21 - 7.40 (m, 3H), 7.02 - 7.18 (m, 2H), 6.97 (br. s., 1H), 6.80 (br. s., 1H), 6.57 (br. s., 1H), 4.93 - 5.24 (m, 1H), 3.82 (td, J = 6.96, 11.17 Hz, 1H), 3.64 (br. s., 1H), 2.84 - 3.08 (m, 11H), 2.14 - 2.32 (m, 1H), 1.99 (tt, J = 6.40, 12.67 Hz, 1H), 1.61 - 1.93 (m, 2H).

ESC1002838 and ESC1002839 were obtained by SFC chiral separation of ESC1002775.

ESC1002776

Procedure 39 with (3-fluoro-5-hydroxyphenyl)boronic acid in place of (4-fluoro-3-hydroxyphenyl)boronic acid. The reaction mixture was irradiated to 150 °C for 20 min. The reaction mixture was filtered through celite which was washed with EtOAc. The EtOAc was

washed with water, dried (Na_2SO_4), filtered and concentrated in vacuo. The resulting material was purified by HPLC (acidic prep). The material was further purified by HPLC (base prep.). The relevant fractions were collected and concentrate in vacuo, and dried for 18 h at 50 °C in vacuo giving the desired compound (11.7 mg, 11%). NMR studies indicated that the product existed as a mixture of rotamers. High temperature NMR studies were conducted to confirm this. 1H NMR (400 MHz, DMSO-d6, 100 °C) δ 7.63 (d, J = 4.27 Hz, 1H), 7.29 - 7.43 (m, 2H), 7.20 - 7.29 (m, 1H), 7.08 (d, J = 7.03 Hz, 1H), 6.96 (br. s., 1H), 6.22 - 6.64 (m, 3H), 5.11 (br. s., 1H), 3.83 (td, J = 7.09, 11.17 Hz, 1H), 3.65 (br. s., 1H), 2.13 - 2.30 (m, 1H), 1.93 - 2.07 (m, 1H), 1.66 - 1.93 (m, 2H)

ESC1002800 and ESC102801

Procedure 27. Once heating was completed, the mixture was plugged on silica (~1 g), and eluted with EtOAc. Then the organic phases were reduced and charged on a 10 g silica cartridge and purified on an Isolera using Hep:EtOAc (0-50%) as eluant. The desired fractions were collected and reduced to afford the product in 72% yield. 30mg was kept and the remaining 48mg were separated. The compound was separated via chiral SFC. On an Analytical Ad-H column 4.6 x 250 mm: flow rate 5 mL/min - injection volume 20 μL - Co solvent Methanol 14%. 1H NMR (373K, 400 MHz, DMSO-d6): 7.63 (d, J = 4.27 Hz, 1H), 7.29 - 7.43 (m, 2H), 7.20 - 7.29 (m, 1H), 7.08 (d, J = 7.03 Hz, 1H), 6.96 (br. s., 1H), 6.22 - 6.64 (m, 3H), 5.11 (br. s., 1H), 3.83 (td, J = 7.09, 11.17 Hz, 1H), 3.65 (br. s., 1H), 2.13 - 2.30 (m, 1H), 1.93 - 2.07 (m, 1H), 1.66 - 1.93 (m, 2H) . LCMS long method, rt = 2.52 min, m/z [M+H]+ 402.2

Procedure 40 (ESC1002802)

2-fluoro-N-isobutyl-N-(5-(methylamino)pyridin-3-yl)benzamide (5 mg, 0.02 mmol) and 4-(chloromethyl)-4H-imidazole (0.01 g, 0.03 mmol) were dissolved in MeTHF. DIPEA (0.02 ml, 0.15 mmol) was added and the reaction mixture was allowed to stir at room temperature for 1 hour. The reaction mixture was heated to 70 °C and allowed to stir for three hours. MeCN (2 mL) was added to the reaction mixture which was allowed to stir at room temp for 2 hours. The reaction mixture was partitioned between water and EtOAc and the phases were separated. The aqueous layer was extracted with EtOAc and the combined organic layers were dried (Na_2SO_4), filtered and concentrated in vacuo giving 30 mg of residue. The mixture was purified by HPLC (acidic prep, rT = 5.22 min), the relevant fractions were collected and concentrated in vacuo giving <5 mg of product. The aqueous layer (pH 6-7) was basified to pH 9 with 2M NaOH (aq.) and extracted with EtOAc. The aqueous layer was further basified to pH 14 and extracted with EtOAc. The aqueous layer was concentrated in vacuo giving a solid residue. This was extracted with MeOH, filtered and concentrated in vacuo. The resulting

residue was purified by HPLC (acidic prep, rT = 5.22 min), and the relevant fractions were concentrated in vacuo. This material was combined with the material from earlier HPLC runs, the combined material was dried at 50 °C for 18 h in vacuo giving a white solid (17 mg, 21%). NMR experiments indicated that this material exists as rotamers. High temperature NMR experiments confirmed that the structure was correct. 1H NMR (400 MHz, 100 °C, DMSO-d6) δ 8.71 (br. s., 1H), 7.96 - 8.03 (m, 1H), 7.71 (s, 1H), 7.23 - 7.36 (m, 2H), 7.19 (s, 1H), 6.85 - 7.12 (m, 4H), 4.54 (s, 2H), 3.70 (d, J = 7.28 Hz, 2H), 2.89 - 3.00 (m, 3H), 1.71 - 1.91 (m, 1H), 0.88 - 0.94 (m, 5H), 0.84 - 1.00 (m, 1H).

Procedure 41 (ESC1002804)

(R)-2-(3'-methoxy-[1,1'-biphenyl]-2-yl)pyrrolidine (330 mg, 1.3 mmol) and 3-chlorothiophene-2-carboxylic acid (0.42 g, 2.61 mmol) were dissolved in MeTHF (5 mL). DIPEA (0.53 ml, 3.91 mmol) and HATU (0.49 g, 1.3 mmol) were added to the reaction mixture which was allowed to stir at room temperature for 18h. The reaction mixture was diluted with water and EtOAc and the phases were separated. The aqueous phase was extracted with EtOAc (x2) and the combined organic layer was dried (Na_2SO_4), filtered and concentrated in vacuo onto silica gel. The material was purified by flash column chromatography (0-40% EtOAc in n-heptanes). Giving material with minor impurities (270 mg, 52%). An alliquot of material was purified further by HPLC (basic prep, RT = 9.33 min), the relevant fractions were collected and concentrated in vacuo. The material was dried at 50 °C in vacuo for 18 h giving the desired compound as a white solid. 1H NMR (400 MHz, DMSO-d6) δ 7.63 (d, J = 5.27 Hz, 1H), 7.21 - 7.53 (m, 4H), 7.09 (d, J = 7.78 Hz, 1H), 6.89 - 7.04 (m, 2H), 6.75 (br. s., 1H), 5.12 (br. s., 1H), 3.63 (br. s., 1H), 2.88 - 3.02 (m, 1H), 2.07 - 2.25 (m, 1H), 1.91 - 2.06 (m, 1H), 1.67 - 1.90 (m, 2H)

ESC1002817

Procedure 37 with N-isobutyl-4-methyl-N-[4-(methylamino)pyrimidin-2-yl]benzamide (25 mg, 0.08 mmol), 4-(chloromethyl)-1H-imidazole;hydrochloride (25.64 mg, 0.17 mmol), Cs_2CO_3 (40.95 mg, 0.13 mmol) and NaI (0.31 mg, 0 mmol). Heating was continued over the weekend. An additional 1.5eq of base was added and heating continued. Additional base (3eq) and chloride (2eq) were added and heating continued overnight. The crude mix was filtered and purified on basic HPLC and the fraction corresponding to the product (by LCMS) was collected and concentrated in the genevac. NMR and LCMS suggest material is consistent with desired product and of sufficient purity for testing (though small baseline impurities are visible in the NMR). ~ 5 mg obtained. 1H NMR (400 MHz, MeOD) ppm 0.93 (d, 6H, J = 6.72Hz), 2.05 (m, 1H), 2.29 (s, 3H), 2.87 (bs, 3H), 3.91 (d, 2H, J = 7.4Hz), 4.40 (s, 2H), 6.35 - 6.41 (m, 1H), 6.75

(s, 1H), 7.05 (d, 2H, J = 7.95Hz), 7.21 (d, 2H, J = 7.95Hz), 7.60 (s, 1H), 7.91 (d, 1H, J = 6.24Hz) LCMS (long method): TR = 1.598 mins, m/z = 379.2 [M+H]+

<u>ESC1002840 and ESC1002841</u>

Procedure 39 with 2-fluoro-3-hydroxyphenyl)boronic acid (46 mg, 0.29mmol) in place of (4-fluoro-3-hydroxyphenyl)boronic acid. (1 mL). The resulting material was purified by flash column chromatography (0-30% EtOAc in n-heptanes) giving the desired compound as a mixture of enantiomers (35 mg, 32%). The material was resolved by SFC (OD-H, 10mm x 250 mm) giving two enantiomers (peak 1, RT = 4.52 min, ESC1002840-01) (peak 2, RT = 5.36 min, ESC1002841-01). NMR indicates that the compounds have rotational isomers. High temperature NMRs indicate that the compounds are correct.

ESC1002840, 1H NMR (400 MHz, DMSO-d6) 9.38 (br. s., 1H), 7.58 - 7.71 (m, 1H), 7.18 - 7.47 (m, 3H), 7.10 (d, J = 7.28 Hz, 1H), 6.88 - 7.05 (m, 2H), 6.50 (br. s., 1H), 4.86 - 5.07 (m, 1H), 3.79 (td, J = 6.84, 11.17 Hz, 1H), 3.47 - 3.66 (m, 1H), 2.10 - 2.19 (m, 1H), 1.89 - 2.01 (m, 1H), 1.64 - 1.87 (m, 2H)

ESC1002841, 1H NMR (400 MHz, DMSO-d6) δ 9.41 (s, 2H), 7.64 (d, J = 4.77 Hz, 1H), 7.20 - 7.47 (m, 3H), 7.10 (d, J = 7.78 Hz, 1H), 6.87 - 7.06 (m, 3H), 6.50 (br. s., 1H), 4.94 - 5.07 (m, 1H), 3.79 (td, J = 6.74, 11.36 Hz, 1H), 3.50 - 3.68 (m, 1H), 2.09 - 2.22 (m, 1H), 1.88 - 2.03 (m, 1H), 1.60 - 1.87 (m, 2H)

Analytical Methods

Liquid-chromatography-mass spectrometry (LC-MS)

Analytical Method A

LC-MS was performed using an Agilent 6140 Series Quadrupole Mass Spectrometer with a multimode source. Analysis was performed using either a Phenomenex Luna® C18 (2)-HST column (2.5 µm, 50 x 2.0 mm) or a Waters X-select® CSH ™ C18 column (2.5 µm, 50 x 2.1 mm). Mobile phase A contained 0.1% formic acid in 18 MΩ water and mobile phase B contained 0.1% formic acid in HPLC grade acetonitrile. A flow rate of 1.00 mL min^{-1} was used over a 3.75 min gradient starting with 99% mobile phase A gradually increasing to 100% mobile phase B. The samples were monitored at 254 nm.

Analytical Method B

LC-MS was performed using an Agilent 6140 Series Quadrupole Mass Spectrometer with a multimode source. Analysis was performed using either a Phenomenex Luna® C18 (2)-HST column (2.5 µm, 50 x 2.0 mm) or a Waters X-select® CSH ™ C18 column (2.5 µm, 50 x 2.1 mm). Mobile phase A contained 0.1% formic acid in 18 MΩ water and mobile phase B

contained 0.1% formic acid in HPLC grade acetonitrile. A flow rate of 1.00 mL min^{-1} was used over a 5.5 min gradient starting with 99% mobile phase A gradually increasing to 100% mobile phase B. The samples were monitored at 254 nm.

Analytical Method C

LC-MS was performed using an Agilent 6140 Series Quadrupole Mass Spectrometer with a multimode source. Analysis was performed using either a Phenomenex Luna® C18 (2)-HST column (2.5 µm, 50 x 2.0 mm) or a Waters X-select® CSH ™ C18 column (2.5 µm, 50 x 2.1 mm). Mobile phase A contained 0.01M NH_4OH in 18 MΩ water and mobile phase B contained 0.01M NH_4OH in HPLC grade MeOH. A flow rate of 1.00 mL min^{-1} was used over a 3.75 min gradient starting with 99% mobile phase A gradually increasing to 100 % mobile phase B. The samples were monitored at 254 nm.

Supplementary B: Chiral synthetic route and determination

A chiral synthetic route or intermediate was not readily available to prepare enantiomerically pure material; therefore, to obtain and test the two constituent enantiomers a semi-preparative chiral SFC method was developed that allowed efficient separation of the enantiomers. The enantiomers were tested in the 17β-HSD10 dose response assay.

Table S1: ESC1002082 enantiomers and their pIC50 values

Identifier	Enantiomer	17β-HSD10 pIC50
**ESC1002082**	Racemic	6.1
**ESC1002161**	Enantiomer 1	<4.7
**ESC1002162**	Enantiomer 2	6.31

Importantly, it was found that all the 17β-HSD10 inhibitory activity resided in a single enantiomer, this not only provided excellent chemical confirmation that the compound was a true inhibitor of 17β-HSD10 activity, but also meant that access to a closely related inactive analogue (which are useful in more complex assays) should be readily available. We subsequently identified that the active enantiomer has an *S*- configuration.

Determining the absolute conformation of the enantiomer

In order to determine the absolute configuration of the active enantiomer, access to the single enantiomer was achieved *via* chiral separation of the racemate by SFC so the absolute configuration of the single enantiomer was unknown. To determine the configuration, a homochiral intermediate was purchased (Sigma Aldrich) and reacted to give ESC1002804 with a known configuration. This compound which has *R* stereochemistry was then tested in the 17β-HSD10 inhibition assay and found to be inactive, pIC_{50} <4.7, by corollary the active enantiomer of the series is therefore *S* configuration.

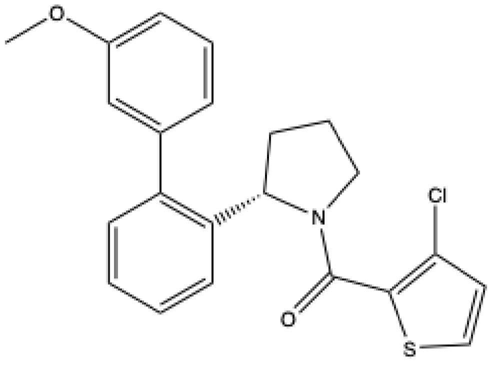

Figure S1: Confirmation of the active enantiomer

Supplementary C:

Mechanism of Action

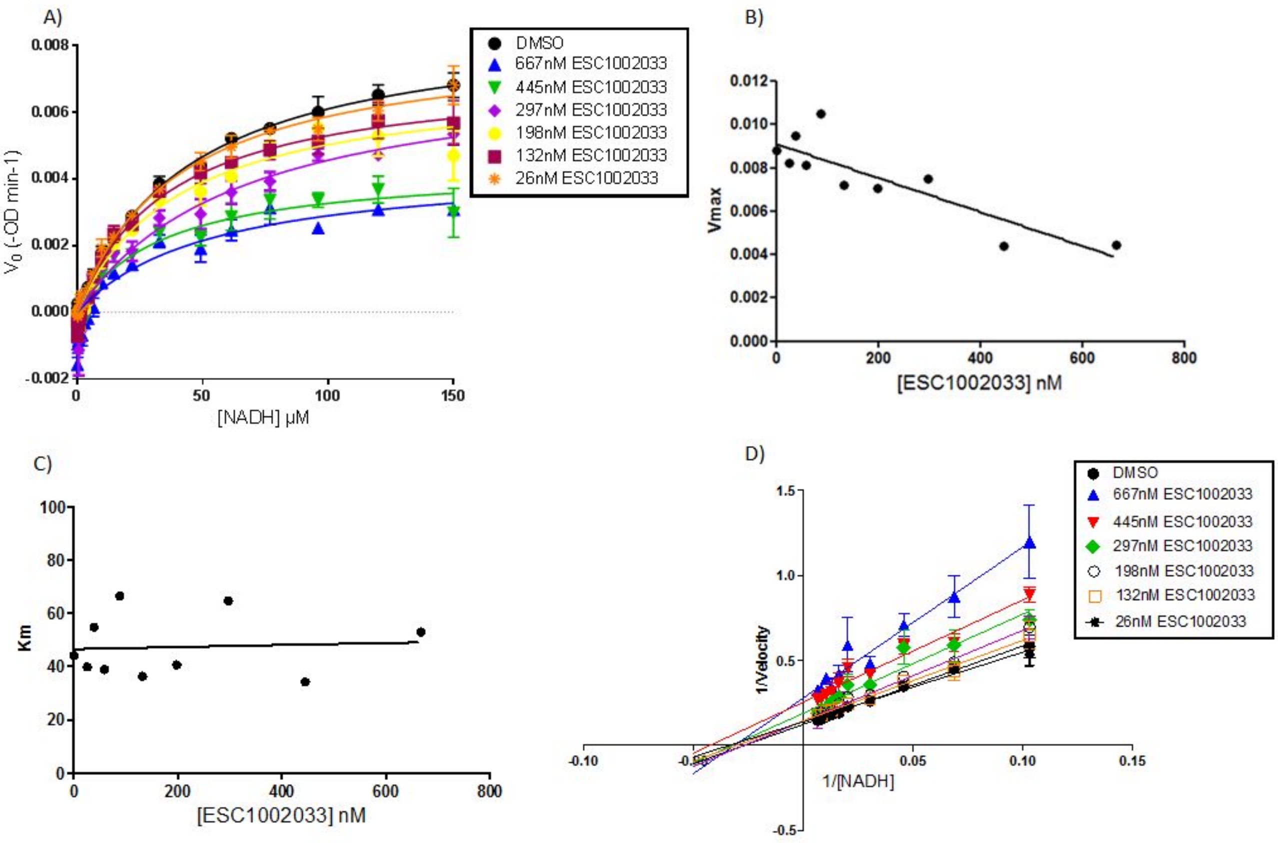

Figure S2: NADH titration of steady state velocity for 17β-HSD10 in the presence of ESC1002033 at varying concentrations. **A)** Untransformed data, **B)** The effect on Vmax with increasing concentrations of ESC1002033, **C)** The effect on Km with increasing concentrations of ESC1002033, **D)** Data as in (**A**) in double reciprocal plot form (Lineweaver-Burke Plot).

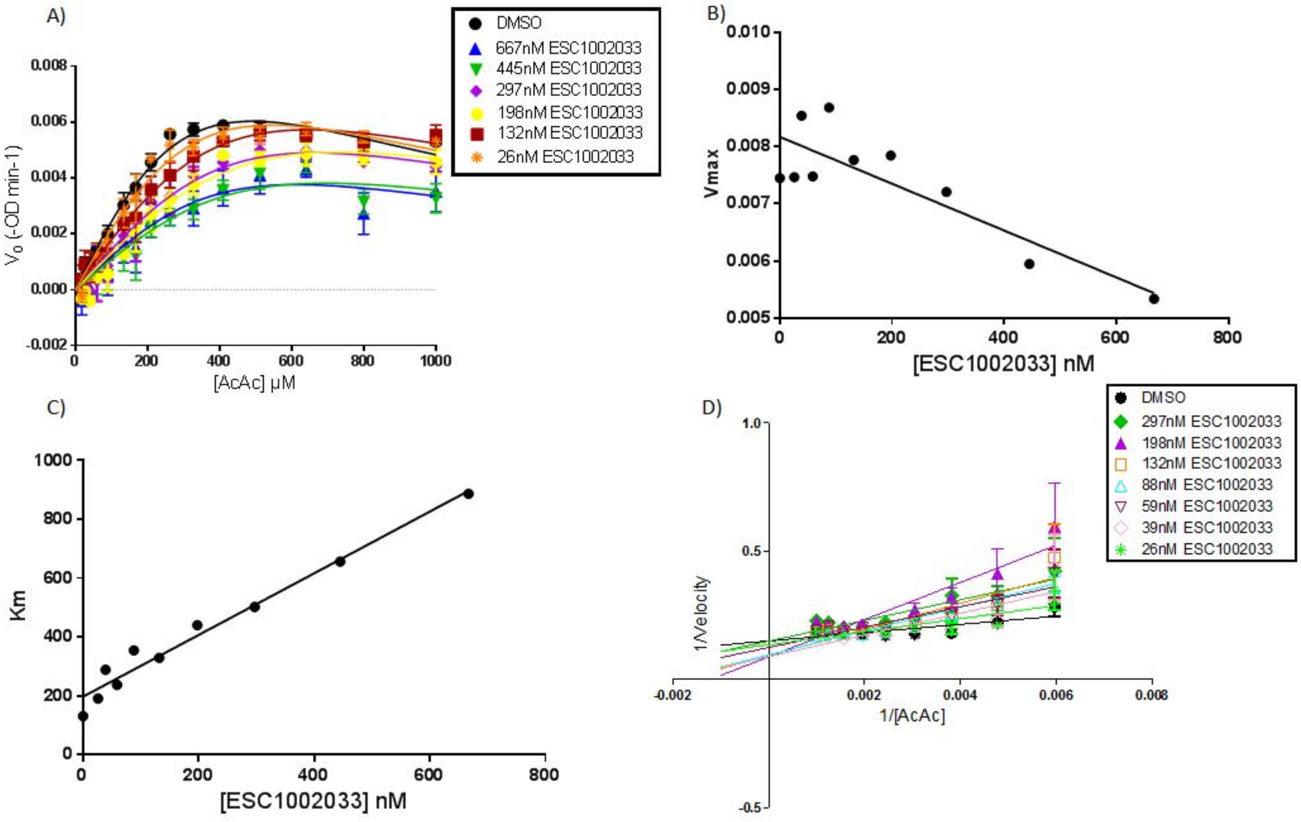

Figure S3: Acetoacetyl CoEnzymeA (AcAc) titration of steady state velocity for 17β-HSD10 in the presence of ESC1002033 at varying concentrations. **A)** Untransformed data, **B)** The effect on Vmax with increasing concentrations of ESC1002033, **C)** The effect on Km with increasing concentrations of ESC1002033, **D)** Data as in (**A**) in double reciprocal plot form (Lineweaver-Burke Plot).

Supplementary D:

Crystallography and Molecular Modelling

Crystal structures of representatives from both analogue series were obtained. The X-ray data for all complexes was of high quality with well-defined electron density for the ligands (see Table D2a, b, c). Molecular replacement was carried out using Phaser and model building/refinement were carries out with CCP4/Coot/Phenix. Refinement statistics for the models are shown below.

Tables S2-S4**:** X-ray crystallography data resolved for three ligand-compound structures (a, b, c).

**ESC1002033**	
Wavelength (Å)	0.9795
High resolution limit	2.73 (2.78 - 2.73)
Low resolution limit	46.58 (2.73 - 2.78)
Completeness	100
Multiplicity	10.24
CC-half	0.994
I/sigma	9.37
Rmerge(I)	0.2643
Anomalous completeness	98.85
Anomalous multiplicity	5.23
Unit cell dimensions: a (Å)	131.06
b (Å)	131.06
c (Å)	132.46
α (°)	90
β (°)	90
γ (°)	120
Space group	P 61
Twinning score	1.93

**ESC1002421**	
Wavelength (Å)	0.9795
High resolution limit	2.18 (2.22 - 2.18)
Low resolution limit	59.02 (2.18 - 2.22)
Completeness	100
Multiplicity	9.8
CC-half	0.9986
I/sigma	12.83
Rmerge(I)	0.1167
Anomalous completeness	98.18
Anomalous multiplicity	5.01
Unit cell dimensions: a (Å)	131.77

b (Å)	131.77
c (Å)	132.84
α (°)	90
β (°)	90
γ (°)	120
Space group	P 61
Twinning score	2

**ESC1002332**	
Wavelength (Å)	0.9795
High resolution limit	2.16 (2.20 - 2.16)
Low resolution limit	52.26 (2.16 - 2.20)
Completeness	100
Multiplicity	10.29
CC-half	0.998
I/sigma	11.9
Rmerge(I)	0.1598
Anomalous completeness	99.74
Anomalous multiplicity	5.22
Unit cell dimensions: a (Å)	131.33
b (Å)	131.33
c (Å)	132.6
α (°)	90
β (°)	90
γ (°)	120
Space group	P 61
Twinning score	1.96
